# Understanding and targeting resistance mechanisms in cancer

**DOI:** 10.1002/mco2.265

**Published:** 2023-05-22

**Authors:** Zi‐Ning Lei, Qin Tian, Qiu‐Xu Teng, John N. D. Wurpel, Leli Zeng, Yihang Pan, Zhe‐Sheng Chen

**Affiliations:** ^1^ Precision Medicine Center Scientific Research Center The Seventh Affiliated Hospital Sun Yat‐Sen University Shenzhen P. R. China; ^2^ Department of Pharmaceutical Sciences College of Pharmacy and Health Sciences St. John's University Queens New York USA

**Keywords:** cancer therapy, combination therapy, drug resistance, resensitization

## Abstract

Resistance to cancer therapies has been a commonly observed phenomenon in clinical practice, which is one of the major causes of treatment failure and poor patient survival. The reduced responsiveness of cancer cells is a multifaceted phenomenon that can arise from genetic, epigenetic, and microenvironmental factors. Various mechanisms have been discovered and extensively studied, including drug inactivation, reduced intracellular drug accumulation by reduced uptake or increased efflux, drug target alteration, activation of compensatory pathways for cell survival, regulation of DNA repair and cell death, tumor plasticity, and the regulation from tumor microenvironments (TMEs). To overcome cancer resistance, a variety of strategies have been proposed, which are designed to enhance the effectiveness of cancer treatment or reduce drug resistance. These include identifying biomarkers that can predict drug response and resistance, identifying new targets, developing new targeted drugs, combination therapies targeting multiple signaling pathways, and modulating the TME. The present article focuses on the different mechanisms of drug resistance in cancer and the corresponding tackling approaches with recent updates. Perspectives on polytherapy targeting multiple resistance mechanisms, novel nanoparticle delivery systems, and advanced drug design tools for overcoming resistance are also reviewed.

## Graphical abstract

The reduced responsiveness of cancer cells can be associated with various mechanisms, including drug inactivation, reduced intracellular drug accumulation by reduced uptake or increased efflux, drug target alteration, activation of compensatory pathways for cell survival, regulation of DNA repair and cell death, tumor plasticity, and the regulation from tumor microenvironments. Various targeting strategies against different resistance mechanisms have been developed. The purpose of overcoming the drug resistance of cancer cells is to optimize the sensitivity of the therapy. This can be achieved by polytherapy using the combination of at least two drugs; immunotherapy using checkpoint inhibitors or monoclonal antibodies; antibody‐drug conjugates improving the selectivity of cancer treatment; gene technology modifying the epigenetic sequence; targeted therapy targeting the overexpression of drug efflux transporter or vital proteins for the cancer cell apoptosis; and nanoparticle delivery system improving the efficacy of the drug and reducing the side effect.

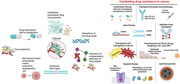

## 1 INTRODUCTION

Cancer remains a global health burden, ranking first or second in the leading causes of death before the age of 70 years in more than 60% of the countries worldwide.^1^ In 2020, there were an estimated 19.3 million newly diagnosed cases and 10 million cancer deaths globally.^2^ Currently, the major therapies for cancer include surgery, radiation therapy, chemotherapy, hormone therapy, targeted therapy, and immunotherapy, either as monotherapy or in combination.^3^, ^4^, ^5^, ^6^ Despite the great development and improvement of cancer treatment in recent decades, resistance to cancer therapies has been a commonly observed phenomenon in clinical practice.^7^, ^8^ Moreover, cancer cells with resistant characteristics often exhibited cross‐resistance to a variety of anticancer drugs that can be structurally irrelevant, namely, the multidrug resistance (MDR) phenomenon.^9^ MDR has been a major obstacle impeding therapeutic success and a dominating cause of cancer relapse and cancer‐related death.

**FIGURE 1:**
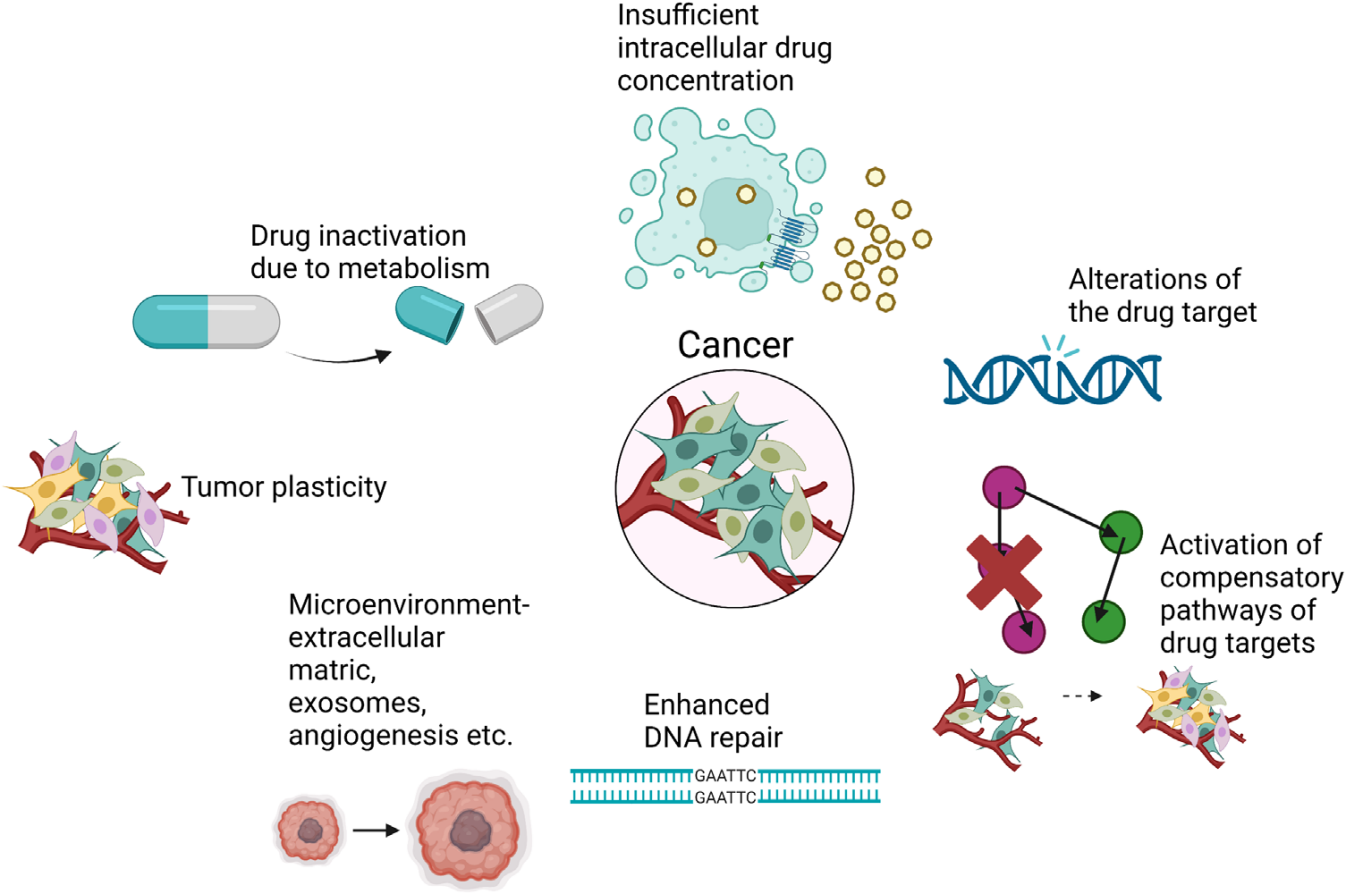
Cancer resistance mechanisms, including drug inactivation, insufficient intracellular drug concentration, drug target alterations, compensatory pathways activation, DNA repair enhancement, and tumor plasticity. *Source*: This figure was created with Biorender.com.

Cancer therapeutic resistance can be categorized into intrinsic and acquired resistance based on the timeline of resistance occurrence. The intrinsic resistance, also known as primary resistance, is mediated by the endogenous factors that are present in tumor cells or tissues before therapeutic applications, which provide cancer cells with survival advantages and adaptability to primary therapeutic stress.^10^, ^11^, ^12^ However, acquired drug resistance is developed after receiving cancer treatment, which is generally mediated by the adaptive alterations against the given therapy in initially sensitive tumors, resulting in compromised treatment effectiveness.^13^, ^14^, ^15^ The reduced responsiveness of cancer cells can be associated with various mechanisms, which usually involve the coactions of genetic factors and nongenetic contributors. Genetic factors in tumor cells have been considered critical contributors to therapeutic resistance, such as genetic diversity, acquired mutations of drug targets, amplification of oncogenes in compensatory or bypass pathways, and epigenetic modifications, which can further affect intratumor heterogeneity, tumor cell plasticity, DNA repair, and the susceptibility of tumor cells to cell death pathways, leading to multifactor‐mediated resistance.^16^, ^17^, ^18^ However, resistant cases in the absence of genetic alteration have been extensively recognized in different types of cancers.^19^ Phenotype changes may be independent of genotype alteration in resistance mediated by metabolic inactivation of drugs,^20^, ^21^ reduced intracellular drug concentration by transporters,^22^, ^23^, ^24^ drug compartmentation,^25^ and drug‐induced reversible transcriptional or posttranslational regulations on adaptive pathways. In addition to the factors within the tumor cells, the tumor microenvironment (TME) is also considered to be involved in the development of resistance in some cancers^26^ (Figure 1).

Notably, the resistance mechanisms are not mutually exclusive and can act jointly to induce therapeutic irresponsiveness in cancer. Therefore, to develop effective strategies to obviate cancer resistance, it is an urgent need to gain a better understanding of the mechanisms collectively. In this review, we will discuss the discoveries of the diverse mechanisms of cancer resistance, the approaches to improve anticancer efficacy via targeting specific mechanisms, and the progress in developing polytherapy fighting against multiple resistance mechanisms.

## 2 RESISTANCE MECHANISMS IN CANCER AND COMBATING STRATEGIES

Drug resistance in cancer can arise from genetic, epigenetic, and microenvironmental factors. A better understanding of the molecular mechanisms underlying cancer resistance is necessary to develop effective strategies to overcome it. In the following context, the identified resistance mechanisms will be discussed with reviews of recent research updates, followed by the development of corresponding combating approaches, such as novel drug designs, combination therapies with specific targets, tackling alternative signaling pathways, and the modulation of the TME.

### 2.1 Metabolism‐associated drug inactivation

The activation and deactivation processes of many chemotherapeutic agents are regulated by drug‐metabolizing enzymes (DMEs).^27^ Dysregulation of DMEs and metabolic signaling pathways, which can lead to the detoxification of drugs or failure in the conversion of drugs into active metabolites, is one of the major mechanisms for chemoresistance in cancer.^28^

Some anticancer drugs require activation by metabolic enzymes. For example, the biotransformation of irinotecan into the active metabolite SN‐38 is catalyzed by carboxylesterase,^29^ and the thymidine phosphorylase is responsible for the activating metabolism of 5‐fluorouracil (5‐FU) into fluorodeoxyuridine monophosphate.^30^ Cytarabine (AraC), a nucleoside drug used in patients with acute myeloid leukemia (AML) and non‐Hodgkin's lymphoma, relies on the phosphorylation catalyzed by deoxycytidine kinase (DCK) to become the cytotoxic form cytarabine triphosphate,^31^ and the deficiency of DCK has been considered to be associated with AraC resistance in AML.^32^ Wu et al. reported that DCK mutations were found in 4 of 10 patients with AML relapse after complete remission and high‐dose AraC post‐remission treatment.^33^ In vitro studies using drug‐selected AraC‐resistant AML and lymphoma cell models indicated that mutation or deficient expression and function of DCK could be acquired after receiving AraC therapy, resulting in the reduced cellular response to AraC and possible cross‐resistance to other nucleoside drugs like gemcitabine.^33^, ^34^, ^35^ A potential strategy to overcome DCK deficiency‐mediated nucleoside drug resistance is using nucleoside analog phosphate prodrugs with anticancer efficacy without the demand for DCK phosphorylation,^36^ among which NUC‐1031, a gemcitabine phosphoramidate prodrug, has entered clinical trials for the treatment of gemcitabine‐resistant cancers.^37^, ^38^ Other reported attempts in preclinical evaluations include enhancing DCK activity by etoposide in combination with AraC for the treatment of AML,^39^ and bypassing the drug resistance mechanism associated with downregulated DCK expression using second‐generation deoxyadenosine analog clofarabine in acute lymphoblastic leukemia cells.^40^

The other aspect of metabolism‐associated anticancer drug inactivation is the detoxification of drugs by metabolizing enzymes. For instance, certain isoforms of aldehyde dehydrogenases (ALDH), including ALDH1A1 and ALDH3A1, have been reported to specifically cause resistance against chemotherapeutic drugs of nitrogen mustard type, such as cyclophosphamide, mafosfamide, and ifosfamide.^27^ In contrast, cytochrome P450 (CYP450) in phase I metabolism, and glutathione‐*S*‐transferase (GST) as well as uridine diphosphoglucuronosyltransferase (UGT) in phase II conjugating biotransformation, are involved in the inactivation of a broader spectrum of anticancer drugs.^41^, ^42^

The CYP450 enzyme superfamily consists of 57 members that are responsible for the phase I oxidation of most clinically used drugs.^43^ Of the CYP450 enzymes, CYP1B1, 2C8, 3A4, and 3A5 have been reported to be correlated to cancer resistance,^44^, ^45^ with CYP1B1 being mostly studied, which is found to be exclusively overexpressed in various types of cancers but has relatively low expression in normal tissues.^46^ Intratumoral CYP1B1 overexpression may contribute to the diminished effectiveness of a diversity of chemotherapeutic drugs, such as paclitaxel and docetaxel, mitoxantrone, flutamide, and gemcitabine.^47^, ^48^, ^49^, ^50^ However, whether the resistance against these drugs is contributed by CYP1B1‐catalyzed drug inactivation remains controversial. McFadyen et al. suggested that docetaxel was metabolized by CYP1B1, and the docetaxel resistance in CYP1B1 overexpressing Chinese hamster ovary cancer cells could be reversed by a CYP1 inhibitor.^51^, ^52^ However, Martinez et al. later demonstrated that CYP1B1 did not directly inactivate docetaxel because the cytotoxicity of docetaxel in MCF‐7 breast cancer cells was not affected by silencing CYP1B1 or adding recombinant CYP1B1.^53^ Although it is possible that CYP1B1 may play different roles in regulating drug resistance in different cancer types, the inhibition of CYP1B1 activity has been considered to be a therapeutic target for improving chemotherapy.^54^ Although lacking high selectivity, phytochemicals are the most common source of CYP1B1 inhibitors, including stilbene, flavonoids, coumarins, anthraquinones, and alkaloids.^55^, ^56^, ^57^ By modifying the structures of phytochemicals, highly potent and selective inhibitors of CYP1B1 have been developed and under preclinical evaluation, such as TMS ((*E*)‐2,3′,4,5′‐tetramethoxystilbene)^58^ and α‐naphthoflavone derivatives.^59^

GSTs are involved in drug detoxification in phase II metabolism and catalyze the glutathione (GSH) conjugation to drugs.^60^ Overexpression of GSTs in cancer cells may enhance the detoxification of anticancer drugs.^61^ Moreover, after GSH conjugation, the conjugated drugs may become substrates of ATP‐binding cassette (ABC) transporters, particularly the MDR‐associated proteins (MRPs, belongs to ABCC subfamily), and got actively pumped out of the cancer cells.^62^, ^63^, ^64^ Among the GST superfamily, GST alpha 1 (GSTA1), GST Mu 2 (GSTM2), and GST Pi 1 (GSTP1) have been found to be associated with cisplatin resistance in ovarian, lung, and gastric cancers.^65^ Notably, the overexpression of GSTP1 has been considered a factor impairing the efficacy of platinum drugs like cisplatin, carboplatin, and oxaliplatin by promoting the formation of platinum‐GSH conjugates.^66^, ^67^ The I150V polymorphism of GSTP1 with a phenotype of reduced enzymatic capacity has been correlated with better therapeutic outcomes in gastric cancer patients receiving oxaliplatin‐based chemotherapy.^68^, ^69^ Besides platinum drugs, the sensitivity to doxorubicin and various alkylating agents in cancer may also be affected by the detoxification process mediated by GSTA subclass, GSTP1, and GSTM1 enzymes.^70^, ^71^ However, additional mechanisms other than GSH conjugation may be involved in GST‐related chemoresistance to doxorubicin and alkylators, such as free radical scavenging and apoptosis suppression via the inhibition of the mitogen‐activated protein kinase (MAPK) pathway.^72^, ^73^, ^74^ Thus, there has been an increasing focus on targeting the GSTs to overcome chemoresistance. Inhibitors of GSTs have been shown effective in treating resistant cancer cells. Ethacrynic acid and analogs are among the earliest investigated GST inhibitors that exhibited resensitizing effects on tumor cells to alkylating agents via covalent binding to GSTs and reducing the enzyme activity.^75^, ^76^ NBDHEX (6‐(7‐nitro‐2,1,3‐benzoxadiazol‐4‐ylthio)hexanol) is another potent inhibitor against GSTP1, GSTM2, and other GST isoenzymes,^77^ which has been found beneficial in combating cisplatin‐resistant osteosarcoma when used in combination with cisplatin.^78^ Apart from inhibiting GST activity, NBDHEX can also induce the dissociation of GSTP1 from its complex with c‐Jun N‐terminal kinase or tumor necrosis factor receptor‐associated factor 2 thereby promoting the activation of apoptosis pathways in cancer cells.^79^ However, one of the significant roadblocks that GST inhibitors encounter in clinical trials is their insufficient specificity, prompting the development of novel NBDHEX analogs with improved selectivity, among which MC3181 was screened out as a specific GSTP1‐1 inhibitor and tested highly efficient in inhibiting vemurafenib‐resistant melanoma in vitro and in vivo.^80^, ^81^ Although the GST inhibitors showed promising effects on inducing cell apoptosis via inhibiting GST activities, investigations on combinations with chemotherapeutic drugs are lacking; thus, whether the inhibitors can reduce drug inactivation remains to be determined. On the other hand, utilizing prodrugs like canfosfamide (TLK286) and brostallicin, which are activated by GSTs to become cytotoxic, has entered clinical evaluations as a practical strategy to circumvent the GST‐mediated resistance mechanism.^82^, ^83^

Similar to GSTs, the UGT enzymes are involved in phase II metabolism, and they catalyze the glucuronidation process. Among the three subfamilies (UGT1A, 2A, and 2B) of the UGTs, UGT1A enzymes, particularly UGT1A1, have been shown to overexpress in tumor tissues and play a role in resistance to anticancer drugs.^20^ UGT1A1 is physiologically responsible for facilitating bilirubin elimination by catalyzing the glucuronic acid conjugation of bilirubin. Elevated tumoral UGT1A1 levels can increase the glucuronidation of SN‐38, the active metabolite of anticancer drug irinotecan, resulting in the inactive SN‐38 glucuronide and reduced therapeutic efficacy.^84^ UGT1A enzymes have also been reported to be associated with primary resistance to some targeted drugs, such as heat shock protein HSP90 inhibitors ganetespib and luminespib^85^ and epidermal growth factor receptor (EGFR) inhibitor erlotinib.^86^ Drug‐induced increase of UGT1A expression has been reported in AML patients treated with ribavirin, which resulted in acquired resistance.^87^ Apart from substrate drugs, the efficacy of monoclonal antibody anticancer drugs may be affected by UGT1As. For instance, UGT1A6 has been found to be correlated with resistance to programmed cell death 1 (PD‐1) antibody nivolumab in patients with advanced renal clear‐cell cancer.^88^ As UGTs are not known to conjugate proteins like antibodies, UGT‐mediated resistance to nivolumab may not be related to directed drug inactivation, but to other mechanisms, such as indirect metabolism regulations by UGTs and other resistance‐correlated signaling regulations involving UGTs.^9^ Because of their association with hampered drug response, the expression of UGT1As might be a useful indicator for patient stratifying so as to avoid the application of substrate drugs to patients who are likely to have low response due to high UGT1A levels. Meanwhile, UGTs can be potential pharmacological targets to overcome drug resistance in cancer. However, the use of UGT inhibitors has been limited by toxic side effects, and selectivity remains the major challenge in developing novel inhibitors of UGTs.^89^, ^90^

Notably, direct detoxification is not the only mechanism of DME‐associated cancer resistance, other involving factors like drug–drug interactions and signaling molecules inherent in cancer cells should be taken into consideration in developing strategies targeting metabolic enzymes in cancer. Regulating the expression and activity of DMEs through regulatory signaling transductions can be beneficial to overcoming cancer resistance not only by means of retaining drug bioavailability but also in other aspects like inhibiting cancer cell proliferation or invasion.

### 2.2 Reduced drug uptake

Drugs can cross the cell membrane through diffusion, endocytosis, or transporters, which can be affected by the permeability and lipid composition of the plasma membrane, and the functions or expression levels of membrane transporters. Alteration of cell membrane structure can impair drug diffusion across the plasma membrane and the endocytosis process. The plasma membranes from drug‐resistant cancer cells have a different lipid composition compared to those from the parental drug‐sensitive cells: The cholesterol and phospholipid levels are elevated, and the protein/lipid ratio is increased up to 60% in MDR cells compared to sensitive cells.^91^ Besides, the slight alkalic pH of the cytoplasm in MDR cancer cells could attenuate the repulsions between the polar groups from membrane lipids by shielding the negative charges, thereby increasing lipid packing and membrane rigidity.^92^ Therefore, MDR cells have plasma membranes with a relatively lower fluidity and reduced permeability leading to decreased drug absorption.^92^ Chemotherapeutic drugs, including vinblastine, doxorubicin, and cisplatin, are vulnerable to this resistance mechanism.^93^, ^94^, ^95^ Additionally, using model membranes by molecular dynamic simulations, Rivel et al. recently demonstrated that, cancer cell membranes are common with the loss of lipid asymmetry compared to normal cell membranes, which may be a contributor to the slower diffusion of cisplatin into cancer cells.^96^ These findings have suggested that lipid composition assessment may be useful for cancer prognosis, and modulating cell membranes can be a potential strategy to ameliorate drug resistance in cancer.

It has been shown that treating cancer cells with short‐chain ceramides can increase cell membrane permeability and fluidity, resulting in an increased uptake of amphiphilic anticancer drugs such as doxorubicin, either in free form or encapsulated form with lipid‐based nanoparticles.^97^, ^98^ The other way to modulate the lipid composition of the cell membrane is by enhancing sphingomyelinase activity using an agonist, such as daunorubicin, etoposide, and ara‐C, thereby decreasing sphingomyelin levels and increasing ceramide levels, leading to increased membrane fluidity.^99^, ^100^ Moreover, biomimetic cell membrane‐coated nanoparticle delivery systems are gaining increased recognition. Cancer cell membrane‐based nanoparticles contain surface proteins from cancer cells; therefore, they can reduce side effects by specifically targeting cancer cells through homotypic binding.^101^ Fang et al. reported that the uptake of membrane‐coated nanoparticles in human breast cancer MDA‐MB‐435 cells was increased by 20‐fold compared to naked nanoparticles.^102^ Many studies have demonstrated the selectivity and biosafety of cancer cell membrane‐based biomimetic nanoparticles using in vitro and in vivo MDR cancer models,^103^, ^104^, ^105^ suggesting a novel drug delivery strategy to improve therapeutic efficacy for MDR cancer.

Intracellular drug concentrations largely depend on the activity of uptake transporters. The main transporters involved in drug uptake are the drug solute carriers (SLCs). The SLC superfamily consists of more than 400 members categorized into 52 families.^106^ Chemoresistance‐relevant SLCs include the organic anion transporting proteins (OATPs), organic cation transporters, concentrative nucleoside transporters, equilibrative nucleoside transporters, and copper transporters.^107^, ^108^ Reduced uptake of chemotherapeutic drugs can be caused by either genetic variants resulting in truncated uptake transporter proteins with reduced or absent function or acquired expression changes in uptake transporters mediated by pharmacological selection pressures. Among them, OATPs (SLCO family) are mostly studied. In particular, amplified expression of OATP1A2, OATP1B1, and OATP1B3 has been found in various cancer tissues,^109^ which is considered associated with chemosensitivity because of its role in the uptake of several classes of anticancer drugs, such as taxanes, platinum‐based drugs, camptothecin analogs, methotrexate, and some tyrosine kinase inhibitors (TKIs).^110^, ^111^ Downregulation of OATP1B3 was reported in a patient‐derived docetaxel‐resistant prostate tumor xenograft model, where the intratumoral concentrations of docetaxel and cabazitaxel were both lower than chemotherapy‐naive tumors. In contrast, prostate tumors with OATP1B3 expression exhibited an increased uptake of both taxanes and higher chemotherapeutic sensitivity.^112^ Overexpression of OATP1B3 in prostate cancer may be beneficial to chemotherapy but can be detrimental to hormone therapy because OATP1B3 drives testosterone uptake in cancer cells, leading to the development of resistance to androgen deprivation therapy (ADT).^113^ Increased OATP1B3 expression in prostate cancer can be induced by ADT, which provides a clue for sequential therapy design: Upregulated tumor SLCO expression following ADT could potentially enhance the uptake of chemotherapeutic drugs like taxanes and improve treatment response.^114^ Interestingly, the genetic variant of OATP transporters may confer resistance to TKIs via the uptake activity instead of enhancing sensitivity.

Haberkorn et al. recently discovered that cancer‐type OATP1B3 protein, a splice variant of liver‐type OATP1B3, is localized in the lysosomal membrane of colorectal cancer (CRC) cells and contributes to the transport of encorafenib and vemurafenib into lysosomes, resulting in decreased drug concentrations in the cytoplasm and reduced drug efficacy.^115^ Therefore, it is necessary to identify the genotype of intratumorally expressed SLCs in the process of confirming their roles in drug resistance.

Although enhancing drug uptakes by modulating SLC transporters may improve chemosensitivity, it may not be a practical approach in cancer treatment. Due to the ubiquitous expression of SLCs in many tissues, modulating the function of SLCs could interfere with the transport activities of nutrients and xenobiotics in normal tissues, which can lead to unfavorable side effects and drug–drug interactions. On the other hand, besides the uptake of anticancer drugs, modulating SLCs may also influence the nutrient uptake in cancer cells, risking promoting cancer progression and metastasis. The best strategy to overcome the uptake transporter‐mediated resistance in cancer may be to use drugs or delivery systems that can circumvent the aberrant transporter, either with improved diffusion efficacy or the capability to utilize alternative transporters.

### 2.3 Increased drug efflux

In addition to reduced drug uptake, drug efflux mediated by the overexpression of ABC transporters is the major mechanism contributing to insufficient intracellular drug concentration. In humans, the ABC transporter superfamily consists of 49 members categorized into 7 subfamilies (ABCA–ABCG) functioning to transport various substrates, including lipids, ions, peptides, and xenobiotics.^116^ The involvement of ABC transporters in MDR was first discovered in 1976 when ABCB1, which is also known as *P*‐glycoprotein (*P*‐gp) or multidrug resisitance 1 (MDR1) protein, was discovered in mouse MDR cell lines.^117^ To date, at least 13 ABC transporters have been found to directly mediate chemoresistance via the efflux of anticancer drugs.^118^ These ABC transporters mainly distribute on the plasma membrane and are triggered upon binding to substrate drugs, resulting in an ATP hydrolysis–driven conformational change of the transporter and the extrusion of the substrate drugs.^119^ Consequently, the overexpression of these transporters has been correlated with poor chemotherapy response and unfavorable patient prognosis in many different types of cancers. Most studies have focused on the ABC transporters with wide spectrums of substrates, such as ABCB1, ABCG2 (also known as breast cancer resistance protein), and the ABCC subfamily (also known as multidrug resistance‐associated proteins, MRPs) with ABCC1 and ABCC10 as representative members. Many cytotoxic chemotherapeutic drugs can be pumped out of cancer cells by ABCB1 and ABCCs, such as taxanes, vinca alkaloids, and anthracyclines. The spectrum of anticancer drugs ABCG2 confers resistance to largely overlap with that of ABCB1 and ABCCs but with some specificities; anthracenedione (such as mitoxantrone) and camptothecins (such as topotecan and SN‐38) are especially vulnerable to the efflux activity of ABCG2, whereas taxanes and vinca alkaloids are not much affected by ABCG2.^120^ Certain clinically used kinase inhibitors, such as imatinib, nilotinib, dasatinib, palbociclib, and some newly developed targeted drugs, such as OTS964 and ARS‐1620, have also been reported as substrates of ABC transporters.^121^, ^122^, ^123^, ^124^ Moreover, ABCCs generally have high affinities to GSH‐conjugated or glucuronate‐conjugated forms of drugs; therefore, the efflux activity of ABCC transporters may be regulated by phase II metabolic enzymes.^24^ Additionally, for brain tumors and metastatic cancer in the brain, chemosensitivity is also affected by the o expression of ABCB1 and ABCG2 transporters at the luminal membrane of endothelial cells of brain microvessels, which are known to impede anticancer drug delivery across the blood–brain barrier.^125^, ^126^

Ongoing efforts have been taken to develop modulators of ABC transporters in order to overcome this resistance mechanism. Numerous small molecule compounds have been developed and tested as inhibitors against ABC transporters, particularly ABCB1. There have been three generations of ABCB1 inhibitors; however, these inhibitors have failed to enter clinical use as combination therapy with substrate anticancer drugs due to different drawbacks. The first generation of ABCB1 inhibitors, such as verapamil, erythromycin, and cyclosporine A, mostly lacks sufficient therapeutic efficacy, which results in considerable side effects by the required high dosages. Although the second‐generation inhibitors, such as dexverapamil and valspodar, have exhibited improved potency, they failed to proceed to clinical application because of their unwanted interactions with the CYP450 enzymes leading to unfavorable pharmacokinetic profiles. The third generation, exampled by tariquidar and zosuquidar, is able to overcome the selectivity problem; however, the performance in clinical settings turns out to be unsuccessful due to interpatient variability.^127^ In the recent decade, some repurposing targeted anticancer drugs, such as selonsertib, tepotinib, and poziotinib, have been discovered as dual inhibitors for ABCB1 and ABCG2.^128^, ^129^, ^130^ Inhibitors that can antagonize both ABCB1‐ and ABCC1‐mediated resistance have also been reported, exampled by cediranib and CBT‐1 (tetrandrine).^131^, ^132^ Targeting multiple ABC transporters may be more prospective given that co‐expression of different ABC transporters is common in tumor tissues and multitargeting drugs may be beneficial to avoid compensatory upregulation of another transporter induced by selective inhibition of a single transporter.^133^

Silencing the efflux transporters using gene editing technologies such as siRNA and CRISPR/Cas9 system has been increasingly investigated as a novel approach to reducing drug resistance, which can potentially benefit genetic variants with intrinsic ABC transporter‐related resistance.^134^ However, many studies conducted gene editing in cell line‐based settings before the step of tumor xenograft model establishment, which is unlikely to represent gene therapy for patients. Cancer‐targeted delivery remains a major challenge in developing gene therapies against ABC transporters. Another trending strategy against ABC transporter‐dependent MDR is to target the regulatory factors of the ABC transporter expression, including transcriptional regulation and epigenetic modifications on the gene, and posttranslational modifications on the protein. Inhibiting the cancer‐specific regulators of ABC transporters may render an advantage to targeting tumor tissues selectively and increasing safety to normal tissues.^127^ Dysregulated noncoding RNAs, including microRNAs (miRNAs) and long noncoding RNAs (lncRNAs), have been found to be associated with the overexpression of ABCB1, ABCG2, and ABCCs in chemoresistant cancers.^135^ The effect on suppressing ABC transporters and reversing cancer MDR by nanoparticle‐delivered ncRNAs mimics or inhibitors has been verified from in vitro and in vivo studies.^136^, ^137^ Another epigenetic factor‐linked overexpression of ABC transporters in cancer is the hypomethylation of the transporter gene promoter, and evidence has shown that agonists of DNA methyl transferases can induce the hypermethylation of the *ABCG2* promoter resulting in the lower ABCG2 expression and increased intracellular concentration of substrate drugs.^138^, ^139^ Alternatively, the expression of ABC transporters can be modulated by targeting the upstream regulatory pathways. For instance, in breast cancer cells, inhibiting the overexpressed receptor tyrosine kinase‐like orphan receptor 1 (ROR1), which is an upstream regulator of ABCB1 transcription via MAPK/extracellular signal‐regulated kinase (ERK) and p53 pathways, can reduce ABCB1‐mediated drug efflux and resensitize breast cancer cells to doxorubicin.^140^ Another recent study showed that suppressing mitochondrial respiration using methylation‐controlled J protein mimetics can decrease ATP production thereby decreasing the energy for ABCB1 and ABCG2 efflux activity and overcoming chemoresistance in vitro and in vivo.^23^

Indeed, the direct approach to circumvent this resistance mechanism is to avoid using substrate drugs or develop novel anticancer drugs that can bypass the efflux transporters. This may rely on advanced computer‐aided drug designs to modify drug structures of current known and actively reported substrates of ABC transporters.

### 2.4 Alterations of the drug targets and activation of compensatory pathways

**FIGURE 2:**
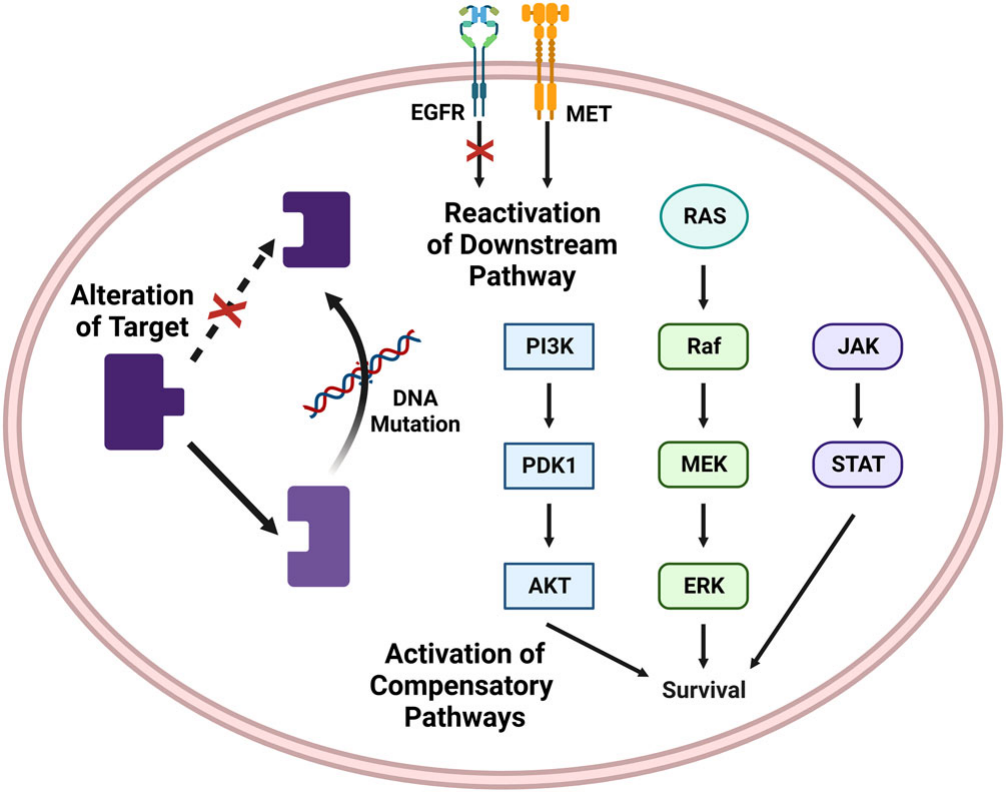
Alteration of drug target and activation of compensating pathways. Cancer resistance associated with alterations in the drug target site or modifications in the structure of the target. The reactivation of the downstream pathway bypasses the other unblocked pathway enabling drug resistance. Activation of compensatory signaling pathways to resist cell death leading to drug resistance. *Source*: This figure was created with Biorender.com.

The leading limitation of targeted cancer therapies is that cancer cells can be intrinsically irresponsive or acquire resistance after a period of treatment because of either the mutation of target molecules (“on‐target” mechanism) or tumor cells gaining survival advantages from a new mechanism independent of the target (“off‐target” mechanism), such as the activation of downstream signaling pathways or compensatory pathways^141^, ^142^ (Figure 2).

The primary approach to overcome “on‐target” mechanism is to develop new generations of targeted drugs against drug‐resistant tumors. For instance, the first‐ and second‐generation EGFR–TKIs, such as gefitinib, erlotinib, and dacomitinib, are less effective in treating patients with additional EGFR mutation T790M.^143^ This has led to structural modification and the development of the third‐generation EGFR–TKIs therapy. Osimertinib (AZD9291), a third‐generation EGFR–TKI, presented a superior clinical response and outcome in EGFR‐mutated non‐small cell lung cancer (NSCLC). However, the rapidly acquired resistance to osimertinib conferred by EGFR C797S mutation has been observed.^144^ Besides, some patients who exhibit primary resistance remain unresponsive to third‐generation and other newly developed EGFR–TKIs. Complete remission is rare, and all patients eventually develop resistance, suggesting that both primary and acquired resistance mechanisms reduce the efficacy of the drug.^145^, ^146^ To reverse these types of resistance, more recently, the fourth‐generation EGFR inhibitors, which can inhibit both T790M and C797S signaling, have been introduced into clinical evaluation.^143^ So far, EAI045 is the first allosteric TKI developed for this purpose. The C797S mutation is unlikely to impair the efficacy of EAI045 because its allosteric binding pocket is not affected by this cysteine residue. EGFR receptor dimerization invalidates drug‐mediated inhibition alone. The activity against T790M and C797S can be restored by a combination regimen with cetuximab, an antibody against EGFR dimerization.^147^ Other fourth‐generation EGFR‐TKIs, such as JND3229 and JBJ‐04‐125‐02, were recently found to be active in EGFR C797S‐T790M‐L858R signal transduction in vitro and in vivo.^148^, ^149^

For the first‐generation tropomyosin receptor kinase (TRK) kinase inhibitors, such as entrectinib and larotrectinib, the secondary mutations occurring at the ATP binding pocket of the TRK kinase domain, including G667C and G595R mutations in *NTRK1* gene, and G696A and G623R mutations in *NTRK3* gene, are common acquired‐resistance mechanisms.^150^, ^151^ In addition, second‐generation TRK inhibitors like selitrectinib have been developed to overcome this acquired resistance.

The multitargeted TKI crizotinib was approved by the Food and Drug Administration (FDA) in 2011 to treat patients with advanced NSCLC harboring ALK rearrangements.^152^ However, despite a high response rate of 60% in ALK‐rearranged NSCLC, most patients develop resistance to crizotinib, typically within 1–2 years. Studies of ALK‐rearranged lung cancers with acquired resistance to crizotinib have identified ALK fusion gene amplification and secondary ALK tyrosine kinase domain mutations in about one third of cases.^153^, ^154^ To date, seven different acquired resistance mutations have been identified among crizotinib‐resistant patients. The most frequently identified secondary mutations are L1196M and G1269A. In addition to these mutations, the 1151T‐ins, L1152R, C1156Y, G1202R, and S1206Y mutations have also been detected in crizotinib‐resistant cancers.^155^, ^156^, ^157^ In approximately one third of crizotinib‐resistant tumors, there is evidence of activation of bypass signaling tracts such as EGFR.^157^ In the remaining one third of crizotinib‐resistant tumors, the resistance mechanisms remain to be identified. Next‐generation ALK inhibitors with improved potency and selectivity compared with crizotinib have been developed to overcome crizotinib resistance in the clinic. The ability of several ALK TKIs (TAE684, AP26113, ASP3026, and CH5424802) to inhibit ALK activity was evaluated in models harboring different ALK secondary mutations.^153^, ^158^ These studies revealed variable sensitivity to these ALK inhibitors depending on the specific resistance mutation present. For example, the gatekeeper L1196M mutation was sensitive to TAE684, AP26113, and ASP3026, whereas 1151T‐ins conferred resistance to all next‐generation ALK TKIs. Ceritinib, an ATP‐competitive, potent, and selective next‐generation ALK inhibitor, has shown favorable selectivity to ALK.^159^ In clinical studies involving ALK‐positive NSCLC patients, ceritinib has exhibited marked antitumor activity has been observed in both crizotinib‐relapsed and crizotinib‐naive patients.^160^, ^161^ On the basis of this impressive clinical activity, ceritinib received FDA approval on April 29, 2014.

Despite increasing successes in efforts to target oncogenic driver amplifications or mutations, several of the most formidable oncogenes and tumor suppressor genes remain undruggable, including RAS (KRAS, NRAS, and HRAS) and RAF (ARAF, BRAF, and CRAF). Effective targeting of KRAS signaling has been tough to realize in patients.^162^, ^163^ Blocking the localization of KRAS at the plasma membrane, a vital element for its activation, has been ineffective because multiple compensatory pathways modulate this process. Similarly, targeting effector signaling downstream of KRAS has not achieved remarkable clinical benefits on the account of paradoxical signaling activation generated by the inhibitor or because of on‐target toxicity limiting the maximum tolerated dose in patients.^164^, ^165^ Tumor cells with KRAS or BRAF^V600E^ mutations are addicted to a downstream ERK signaling cascade for their growth, viability, and other malignant properties.^166^ The adaption of selective inhibitor target KRAS^G12C^ only briefly inhibits KRAS within 1–3 days of treatment, and initial signaling suppression is accompanied by the re‐storage of active KRAS and reactivation of ERK1/2 signaling pathway.^167^, ^168^ Accumulation of active KRAS illustrates that compensatory activation of upstream receptor tyrosine kinases (RTKs), including EGFR, is to a large extent responsible for the adaptive alterations noted during KRAS^G12C^ inhibitor treatment. In RAS^Mut^ tumors, RAF inhibitors (RAFis) are ineffective because they drive paradoxical ERK1/2 pathway activation and adventitious tumor progression.^169^ However, MAPK kinase (MEK) inhibitors (MEKis) do not present the same limitation as RAFis, but the relief of feedback inhibition and pathway reactivation restricts MEKi monotherapy in RAS^Mut^‐driven tumors.^166^, ^170^ By the amplification of KRAS^G13D^ or BRAF^V600E^, CRC cells acquire resistance to the MEKi.^171^ Furthermore, MEKi resistance driven by BRAF^V600E^ amplification is thoroughly reversible upon prolonged drug withdrawal as BRAF^V600E^ amplification brings about a selective disadvantage in the absence of MEKi, and MEKi withdrawal drives ERK1/2 activation beyond a key point that is optimal for cell proliferation and viability.^172^ Remarkably, MEKi resistance driven by KRAS^G13D^ amplification is not reversible. Upon MEKi withdrawal, CRC cells experience an ERK1/2‐dependent epithelial‐to‐mesenchymal transition (EMT) and emerge resistance to frequently used chemotherapeutics instead of exhibiting growth defects.^172^ Consequently, the appearance of MEKi resistance, drug‐addiction, and the possible options of intermittent dosing schedules, may depend on the nature of the amplified oncogenes (e.g., KRAS^G13D^ BRAF^V600E^), further highlighting the hardships of targeting RAS and RAF mutant tumors. Mutations of tumor suppressor can also result in resistance to targeted therapies, for example, mutations in phosphatase and tensin homolog deleted on chromosome 10 (PTEN) can activate phosphatidylinositol‐3‐kinase (PI3K) β signaling in response to PI3Kα inhibitors,^173^ as well as reverse mutations in breast cancer 1 (BRCA1) or BRCA2 in response to poly(ADP‐ribose) polymerase (PARP) inhibitors.^174^

In NSCLC, the majority of all EGFR mutations are EGFR^L858R^ in exon 21 and activating EGFR exon 19 deletions, and tumor cells harboring the specific activating mutations exhibit high sensitivity to EGFR–TKIs.^175^, ^176^ These constitutively activate mutant EGFR oncoproteins signaling by regulating MAPK and PI3K/AKT/mTOR signaling cascades to promote tumorigenesis.^177^ In a series of EGFR–TKI‐resistant tumor samples, 1% of patients exhibited resistance to early‐generation EGFR–TKIs via the acquisition of BRAF^V600E^ or BRAF^G469A^.^178^ Increased mTOR level was related to EGFR–TKI resistance in clinical samples,^179^ and in mouse models, the application of rapamycin (mTOR inhibitor) presented the positive progression of EGFR^mut^ lung tumors.^180^ EGFR–TKIs in combination with PI3K/AKT/mTOR signaling pathway inhibitors have proved improved tolerability and efficacy. Another pattern of acquired resistance to kinase inhibition is the amplification of upstream genes, which can exemplify by mesenchymal–epithelial transition (MET) amplification leading to resistance to EGFR–TKIs.^181^

The clinical value of proto‐oncogene 1 (ROS1)‐directed TKIs was first prospectively explored in NSCLC patients, and in 2016, the FDA and EMA approved crizotinib for the treatment of advanced‐stage ROS1‐rearranged NSCLC.^182^ Afterward, new ROS1–TKIs got into clinical trial, leading to the FDA and Ministry of Health, Labor and Welfare of Japan approvals of entrectinib for the treatment of ROS1‐aberrant NSCLC. So far, all developed ROS1–TKIs are multikinase inhibitors, which can also target MET (e.g., crizotinib and cabozantinib), ALK (e.g., ceritinib and crizotinib), TRK (e.g., entrectinib and repotrectinib), and several other kinases (e.g., SRC, EGFR, JAK2, and FLT3) with equivalent or lower potency.^183^, ^184^, ^185^ Various compensatory pathways involved in TKI therapy of ROS1‐rearranged tumors progression, such as KRAS, NRAS, BRAF, HER2, EGFR, MEK, and MET. For example, mutations of KRAS^G12D^ and BRAF^V600E^ have emerged with crizotinib treatment in the clinical testing,^186^ and NRAS^Q61K^ has occurred while entrectinib treatment.^187^ Upregulated HER2 phosphorylation has been identified in patient‐derived, crizotinib‐resistant CD74–ROS1‐positive CUTO23 (NSCLC cell line), and afatinib combined applied recovered crizotinib sensitivity in CUTO23.^188^ Usually, ROS1 fusions de novo barely co‐occur with EGFR mutations in clinic,^189^ but ROS1 fusions come into sight as resistance mechanisms to EGFR–TKI therapy in EGFR‐mutant NSCLC,^190^ suggesting feedback or potential signaling reciprocity among ROS1 and EGFR. In patients treated with ROS1–TKIs, activating PIK3CA mutations have also been reported.^188^, ^191^ In ROS1 fusion‐positive NSCLCs patients treated with ROS1–TKIs, 11% were found to have concurrent MEK/MAPK alterations.^192^ Subsequently, it was proved that the deletion of MEK1 or MEKK1 in cells conferred resistance to ROS1–TKIs and combined ROS1 and MEKis inhibited cell growth.^192^ Until 2020, MET amplification was found to act as an important participator of ROS1–TKI (e.g., lorlatinib) resistance.^141^ ROS1 kinase domain mutations that trigger the resistance of ROS1 fusion‐positive tumors to ROS1–TKIs have been identified by various preclinical and clinical studies.^188^ Some of these mutations are paralogous to ALK resistance mutations that emerge with targeted therapy for ALK fusions,^193^ among which ROS1^G2032R^ is the most common resistance substitution observed in patients treated with crizotinib.^194^ Considering its clinical benefits but its lack of activity against ROS1^G2032R^, lorlatinib can rebuild enduring disease control in ROS1‐rearranged tumors with the limited spectrum of resistance mutations.^195^ However, FDA has conferred repotrectinib as the Fast Track Designation agent in patients who previously experienced one ROS1–TKI and platinum‐doublet chemotherapy. Up to now, next‐generation ROS1–TKIs have not been approved for the treatment of ROS1 rearrangement‐positive tumors.

### 2.5 Enhanced DNA repair

The DNA damage response (DDR) is a mechanism for repairing drug‐induced DNA damage. Mutation or dysregulation of certain genes and DDR mechanisms are common in many types of cancer and can influence the sensitivity to DNA‐damaging chemotherapy drugs, such as cisplatin and 5‐FU.^196^, ^197^ The nucleotide excision repair (NER) responsible for repairing DNA damage repairing and mismatch repair (MMR) responsible for maintaining genomic integrity are known to be related to 5‐FU resistance. It is demonstrated by Liu et al. that the acquired upregulation of the excision repair cross‐complementing 1 (ERCC1), a component of the NER system, was induced by 5‐FU treatment in gastric cancer cells, which can subsequently weaken the anticancer activity of 5‐FU. Further mechanism studies revealed that 5‐FU‐induced ERCC1 overexpression may be regulated by ERK 1/2 and p38 signaling‐mediated activation of the transcription factor c‐jun/activator protein‐1, which provides a clue to overcome this resistance mechanism using ERK inhibitors or p38 kinase inhibitors.^198^ In contrast, the role of MMR in 5‐FU resistance remains uncertain. It was recently reported by Oliver et al. that both MMR‐proficient and MMR‐deficient CRC cells exhibited 5‐FU resistance after a period of treatment, suggesting that 5‐FU chemoresistance in CRC cells may be independent of MMR status. Nevertheless, MMR genes human homolog 1 (hMLH1), a component of MMR, was upregulated in an MMR‐deficient cell line, which indicated a potential involvement in 5‐FU sensitivity.^199^

MMR deficient–associated microsatellite instability has been linked to cisplatin resistance in clinical germ cell tumor sample,^200^ whereas in ovarian cancer, cisplatin resistance is generally related to homologous recombination repair (HRR) for DNA damage. The *BRCA1* and *BRCA2* tumor suppressor genes are critical for the HRR of DNA double‐strand breaks by the HRR pathway; therefore, BRCA1/BRCA2‐mutated ovarian cancers are usually more sensitive to cisplatin.^201^ However, acquired secondary mutations of BRCA1/2 induced by long‐term cisplatin exposure can reconstruct the function of BRCA1/2 and enhance DNA repair, resulting in resistance to cisplatin^200^ as well as inhibitors of DNA repair poly (ADP‐ribose) polymerase (PARP).^202^ Recently, another DNA repair–related protein, actin‐like protein ACTL6A, has been reported to promote DNA lesion induced by cisplatin damage through the SWI/SNF chromatin remodeling complex in several types of cancers, suggesting a novel cisplatin‐resistance mechanism. As ACTL6A is also a component of NuA4/TIP60 histone acetylase, treatment with a histone deacetylase inhibitor can be useful to attenuate this resistance mechanism and resensitize tumor cells to cisplatin.^203^

In addition to gene mutations that activate DNA repair genes, epigenetic regulations, including DNA methylation, histone modifications, and miRNAs, can all affect the expression of DNA repair genes, contributing to drug resistance.

DNA methylation can suppress gene expression. In the context of DNA repair–associated drug resistance, DNA methylation has been implicated in the regulation of key DNA repair genes, such as *O* ^6^‐methylguanine‐DNA methyltransferase (*MGMT*), *BRCA1/2*, and *MLH1*. *MGMT* promoter methylation is associated with a loss of MGMT protein expression and activity in the tumor and has been shown to correlate with a better outcome of therapy in several studies.^204^, ^205^ Consequently, the methylation status of the *MGMT* promoter has emerged as a useful biomarker for predicting the treatment response of glioblastoma (GBM) patients to methylating chemotherapeutic agents like temozolomide.^206^ In breast cancer, the hypermethylation of the *BRCA1* promoter has been associated with decreased BRCA1 expression and sensitivity to DNA‐damaging drugs and PARP inhibitors.^207^ In contrast, the hypermethylation of the *MLH1* promoter has been associated with decreased MLH1 expression and DNA repair capacity in cancer cells, which can increase the accumulation of somatic mutation in cancer cells and induce drug resistance.^208^ These findings highlight the complex interplay among DNA methylation, DNA repair, and cancer resistance and suggest that targeting DNA methylation may be a promising strategy for overcoming drug resistance in cancer.

Histone modifications also play an important role in DNA repair and cancer resistance. A recent study demonstrated that, in high‐grade serous ovarian carcinoma cell lines and patient‐derived xenograft models, the upregulation of histone methyltransferases EHMT1 and EHMT2 are responsible for the trimethylation of histone H3 lysine 9 (H3K9me3) and contribute to PARP inhibitor resistance through inhibiting the expression of the tumor suppressor gene *CDKN1A*, which is involved in the regulation of the cell cycle and DNA repair.^209^ However, another study showed that the loss of the histone methyltransferase, enhancer of zeste homolog 2 (EZH2), led to decrease in global H3K27 methylation, resulting in the upregulation of genes associated with cisplatin resistance and the downregulation of genes involved in DDR. This study also showed that treatment with EZH2 inhibitors could reverse chemotherapy resistance in TGCTs, indicating the potential for epigenetic therapies in treating drug‐resistant tumors.^210^

MiRNAs and LncRNAs have been implicated in the regulation of multiple pathways, including DNA repair. For example, miR‐15a and miR‐16 are found to downregulate the expression of B‐lymphoma Moloney murine leukemia virus insertion region‐1 protein (BMI1), a protein involved in the ubiquitin‐mediated DNA repair pathway, thereby decreasing DNA repair capacity and increased sensitivity of breast cancer cells to doxorubicin treatment.^211^ Upregulation of LncRNA HOTAIR has been observed in breast cancer cells following radiation therapy, which promotes DNA repair and radioresistance by interacting with the EZH2 protein.^212^ These findings have implicated that the specific miRNAs and LncRNAs could serve as biomarkers for predicting therapeutic response in cancer patients and could be potential target to overcome drug resistance.

### 2.6 Inhibition of cell death

Cancerous cells are known to highly regulate the apoptotic pathways, and apoptosis plays different roles in tumor: eliminates infected cells from the human body, assists the functioning of the immune system, as well as contributes to the maintenance of homeostasis.^213^ Generally, those signals induce the activation of effector caspases that mediate intracellular signaling, resulting in DNA fragmentation. Caspase mutations occurring in tumor therapy chemoresistance have been widely reported. For example, caspase‐8 mutations are mainly detected in gastric cancers, and the procaspase‐8^Q482H^ mutation abrogates apoptosis by leading to the dimerization attenuation of the procaspase‐8 protein monomers, resulting in resistance to chemotherapy.^214^ The intrinsic apoptosis pathway by mitochondrial damage is activated through numerous exogenous or endogenous stimuli that induce DNA damage.^215^ The activation of this pathway involves the activation or inhibition of proteins from the B‐cell lymphoma 2 (Bcl‐2) family, and multiple drugs are known to induce apoptosis of cancerous cells through this mechanism. For example, small molecule RAF kinase inhibitor sorafenib induces apoptosis of AML through the downregulation of Mcl‐1 (an anti‐apoptotic protein) and the upregulation of BIM (an activator of the intrinsic pathway).^216^ Previous studies (in vitro and in vivo) have suggested that upregulating of Bcl‐2 in breast cancer cells promotes metastasis to the lung in mice by the EMT process and triggers resistance to chemotherapy (PD168393, a specific inhibitor of EGFR; AG490, an inhibitor of JAK2).^217^, ^218^ In the treatment of hematological malignancies, the aim to integrate the application of Bcl‐2 inhibitors has led to further research and development of Bcl‐2 selective and dual Bcl‐2 and Bcl‐xL inhibitors. Similar to ABT‐199 (the first‐ever Bcl‐2 inhibitor known as Venetoclax), Navitoclax (ABT‐263) is one of the most studied dual inhibitors; it acts as a BH3‐mimetic, permitting the release of BH3‐only proteins and further MOMP by Bax/Bak activation.^219^ One clinical trial detected the effect of ABT‐263 in 118 chronic lymphocytic leukemia (CLL) patients and reported partial responses in 34.6% of patients in phase I trials.^220^ Rituximab, a monoclonal antibody targeting the protein CD‐20 was combined with ABT‐263 and showed a statistically significant overall response rate of 70% in untreated patients with CLL compared to rituximab alone.^221^ However, because of the character of Bcl‐xL in platelet survival, a main concern with ABT‐263 is the negative effect: inducing thrombocytopenia in a dose‐dependent manner. Furthermore, the additional concern is regarding the emergence of tumor cell resistance to this agent, through the upregulation of Mcl‐1 phosphorylation and further sequestration of the proapoptotic protein Bim.^222^ Overall, the clinical use of ABT‐263 is under restriction, and further studies are necessary before its approval.

Autophagy is a conserved and tightly regulated catabolic self‐degrading mechanism whereby cells keep homeostasis and respond to stress through recycling damaged cellular organelles, proteins, and other cellular components.^223^ In tumors, autophagy is a double‐edged sword, and its anti‐ or pro‐tumorigenic character relies on the oncogenic context and stage of tumorigenesis.^224^ In the early stages of tumorigenesis, autophagy exhibits tumor suppression function by removing aggregated and misfolded proteins, damaged organelles, limiting cell growth, necrosis, and chronic inflammation.^225^ Upon advanced periods of tumor progression, autophagy helps to keep tumor cell survival and increases cellular resistance by conferring stress tolerance by providing the recycling substrate to promote cancer cell survival and contributes to cancer progression and drug resistance.^226^ For example, in ovarian cancer,^227^ leukemia,^228^ hepatocellular carcinoma (HCC),^229^ and CRC,^230^ the increased levels of basal autophagy contribute to tumor cell growth and increase aggressiveness. Another study showed that autophagy was activated in mice with Myc‐induced lymphomas when treated by chemotherapy; autophagy inhibition sensitizes tumor cells to cell death induced by chemotherapeutic agents, suggesting that autophagy induction leads to a self‐defense effect in this case.^231^ Several barriers result from TKI resistances that emerge in a later period of tumor attribute to the mutation in the kinase domain or function of basal autophagy as housekeeping as well as the regulator of RTK activity, which challenges effective treatment against cancer targeted therapy. Therefore, the combination of autophagy modulators with TKIs has been considered in cancer therapy. For example, in NSCLC, erlotinib and gefitinib can induce a high level of autophagy as a drug resistance and cytoprotective mechanism that was accompanied by the suppression of the PI3K/AKT/mTOR signaling cascade. In addition, autophagy inhibition by the pharmacological inhibitor, chloroquine, and siRNAs targeting autophagy‐related gene 5 (ATG5) and ATG7, enhances the erlotinib and gefitinib cytotoxicity.^232^ Gastrointestinal stromal tumors (GISTs), in which the activation of CD117, stem cell growth factor receptor, or platelet‐derived growth factor receptor‐α mutations comes up, rarely answer to only‐imatinib mesylate (IM) treatment. IM triggers autophagy as a survival mechanism in quiescent GIST cells that result in acquired resistance and ineffective treatment. Nevertheless, the combination therapy by which IM was used with siRNAs targeting ATGs potentiates GIST cytotoxicity.^233^ Another study revealed that IM induces autophagy in chronic myeloid leukemia by induction of ER stress in a different way from apoptosis induction and the combination therapy with autophagy inhibition using either siRNA targeting ATGs or pharmacological inhibitors promotes cell death. Moreover, other TKIs like dasatinib or nilotinib, combined with autophagy suppression treatment, exhibited similar effects.^234^

Ferroptosis is an intracellular iron‐dependent mechanism of cell death that is distinct from apoptosis, necrosis, and autophagy.^235^ Extensive preclinical and clinical studies have concentrated on conquering drug resistance, and inducing ferroptosis has been proven to reverse drug resistance. According to the theoretical framework for initiating the process of ferroptosis, there are three diverse pathways to reverse chemotherapy resistance: the canonical glutathione peroxidase 4 (GPX4)‐regulated pathway, iron metabolism pathway, and lipid metabolism pathway.^236^ Genetic inhibition of GPX4 can induce tumor cell ferroptosis and suppress tumor growth in vivo.^237^ In addition, the inactivation of GPX4 results in the accumulation of phospholipid hydroperoxides to induce cell membrane damage and ferroptotic death.^238^ In GBM, androgen receptor ubiquitination induced by the curcumin analog was found to inhibit GPX4, generating ferroptosis and reversing temozolomide resistance.^239^ In CRC, elevated kinesin family member 20A (KIF20A) expression was associated with oxaliplatin resistance, cellular ferroptosis may be induced by disturbing the KIF20A/NUAK1/GPX4 signaling pathway to reverse the resistance of CRC to oxaliplatin.^240^ Cystine/glutamate antiporter (xCT) system, as an essential component of the GPX4‐regulated pathway, has also been revealed to be capable of triggering ferroptosis. A study reported that erastin and sulfasalazine (xCT system inhibitors) could induce head‐and‐neck cancer cell ferroptosis and overcome cisplatin resistance.^241^ Another study showed that in gastric cancer, inducing ferroptosis by restraining Nrf2/Keap1/xCT signaling, can sensitize cisplatin‐resistant cells to cisplatin treatment.^242^ Ferroptosis is defined as an iron‐catalyzed form of regulated necrosis.^243^ Upregulation of cellular labile iron pool (LIP) leads to increase vulnerability to ferroptosis. Du et al. reported that tumor cell ferroptosis contributed to catastrophic LIP accumulation after dihydroartemisinin treatment in pancreatic ductal adenocarcinoma, and dihydroartemisinin treatment could overcome cisplatin resistance by triggering ferroptosis.^243^ Divalent metal transporter 1 (DMT1) and secreted glycoprotein lipocalin‐2 are both key proteins in regulating iron homeostasis. Turcu et al. found that elevated cellular LIP caused by DMT1 inhibition induced ferroptosis, thereby wiping out breast cancer stem cells (CSCs) and reversing MDR.^244^ A recent report by Chaudhary et al. indicated that targeting lipocalin‐2 overcame 5‐FU resistance in CRC by elevating intracellular iron levels, which in turn resulted in tumor cell ferroptosis.^245^ The lipid metabolism pathway is also tightly associated with cell vulnerability to ferroptosis. Acyl–CoA synthetase long‐chain family member 4 (ACSL4), an important participant in ferroptosis execution, is involved in producing phospholipid hydroperoxides in an enzymatic manner.^246^ In pancreatic cancer, the activation of ACSL4 conquered gemcitabine resistance by inducing ferroptosis.^247^

Recently, Tsvetkov et al. revealed a previously uncharacterized cell death mechanism termed cuproptosis.^248^ Cuproptosis a mechanism distinct from all other known manners of regulated cell death, such as apoptosis, pyroptosis, necroptosis, and ferroptosis. Cuproptosis is regulated by protein lipoylation in the citric acid (TCA) cycle, where lipoylation is necessary for enzymatic function.^249^, ^250^ Their research illuminates the connection between the sensitivity to copper‐mediated cell death and mitochondrial metabolism: respiring. TCA‐cycle active cells have upregulated levels of lipoylated TCA enzymes (particularly, the pyruvate dehydrogenase complex), and the lipoyl moiety acts as a direct copper binder, inducing the aggregation of lipoylated proteins and destabilization of Fe–S cluster proteins, ultimately leading to proteotoxic stress and cell death.^248^ Genetic variation in copper homeostasis leads to serious disease, and copper chelators and copper ionophores have been proposed as anticancer agents.^251^, ^252^, ^253^, ^254^ For genetic disorders of copper homeostasis like Menke's disease and Wilson's disease, copper chelation is an effective treatment.^255^ However, in tumors, copper ionophores like elesclomol have been tested in clinical trials, but neither the benefit of a biomarker of the appropriate patient population nor an understanding of the drug's mechanism of action is considered in such testing. For example, a phase III combination clinical trial of elesclomol in melanoma patients indicated the absence of efficacy in this unselected population, yet a post hoc analysis of samples with low plasma lactate dehydrogenase (LDH) levels displayed evidence of antitumor activity.^256^ Low LDH represents a higher cellular dependency on mitochondrial metabolism, in accordance with Tsvetkov's finding that cells more reliant on mitochondrial respiration are nearly 1000‐fold more sensitive to copper ionophores than cells going through glycolysis. Considering the distinct mechanism of cuproptosis from other forms of cell death, deeply understanding how cuproptosis is initiated, progressed, and ultimately executed may exhibit great significance for specific therapeutic interventions and workable combination therapies.^257^

### 2.7 Regulation from tumor microenvironment

**FIGURE 3:**
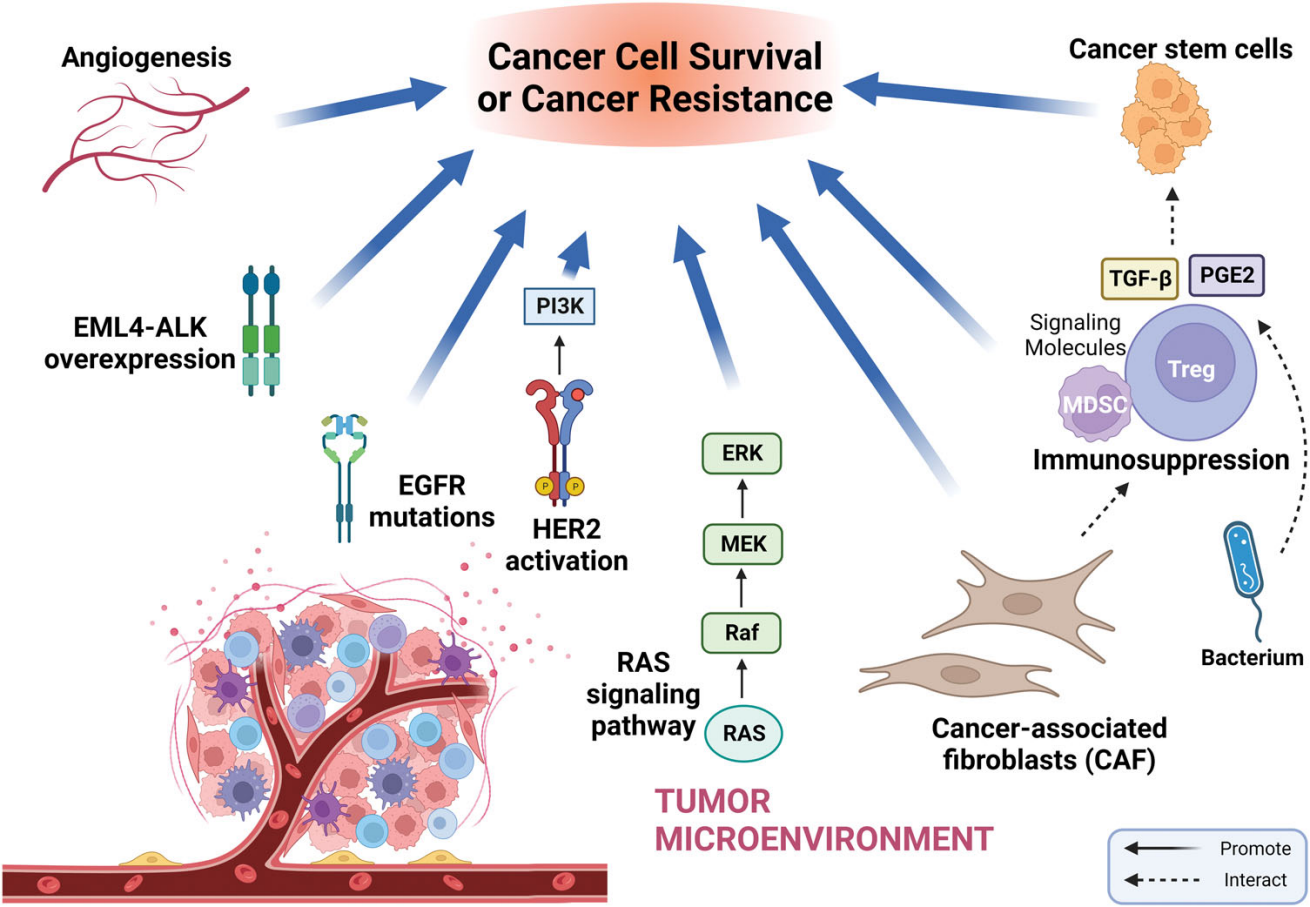
Adaptive mechanisms of cancer cell survival and cancer resistance driven by tumor microenvironment (TME). The TME is important for cancer resistance; the cancer cells within the TME can undergo a series of adaptive changes, such as various cellular components can complement the growth signal of cancer cells, combining with angiogenesis to promote cell survival and resistance. The immunosuppression caused by the TME prevents immune cells from killing cancer cells. The induction of the TGF‐β signaling and the release of prostaglandin E2 (PGE2) resulting in further augmentation of self‐renewal and plasticity of cancer stem cells (CSCs). *Source*: This figure was created with Biorender.com.

How tumor cells react to therapy not merely relies on the genomic aberrations they harbor but also is mediated by the TME.^258^ TME is a complicated element of tumors and is quite heterogeneous.^259^ The significant character of the TME in changing tumor activity has been stated by plentiful studies uncovering how the TME can influence the malignant behavior and therapeutic resistance of the tumor cells^260^ (Figure 3). The TME contains a multitudinous cellular and acellular milieu; diverse stromal and immune cells are recruited to develop and maintain such self‐sustained circumstances.^261^ The extracellular matrix (ECM), including proteins like laminins, fibronectin, proteoglycans, vitronectin, tenascin‐C, and collagen, devotes to the majority component of the TME and is vital for the maintenance of TME and the induction of cellular adhesion, migration, invasion, and metastasis.^262^ Moreover, the composition and organization of ECM can also influence the sensitivity to drug therapy. For example, it has been reported that ECM proteins (laminin, fibronectin, and vitronectin) mediated cell adhesion‐mediated drug resistance (cilengitide, an integrin inhibitor, and/or carmustine, an alkylating chemotherapy) in glioblastoma (GBM). Enrichment of the above proteins in the TME facilitates GBM cell proliferation via integrin α_v_‐mediated FAK/paxillin/AKT signaling cascade and suppresses p53‐involved tumor apoptosis.^263^ In breast cancer, cancer‐associated fibroblasts (CAFs) promote drug resistance by increasing hyaluronan production.^264^ Another study showed that the inhibition of the β1 integrin activity by monoclonal antibody AIIB2 markedly promotes radiotherapy efficacy and elevates sensitivity to HER2‐targeting agents of breast cancer cells.^265^, ^266^ In melanoma, CAFs promote cell metastasis and drug resistance by upregulating the level of matrix metalloproteinase 1 (MMP1) and MMP2.^267^ As a significant aspect of the TME, the cell‐to‐ECM interaction and cellular crosstalk induce the release of soluble factors (like, angiogenic factors and chemokines) in charge of ECM remodeling and immune evasion, which further bring about therapy resistance. For example, the interaction between α4β1 integrin on cancer cells and fibronectin induce drug resistance in AML and CLL through the PI3K/AKT/BCL2 signaling pathway.^268^, ^269^ Stromal cell–derived factor 1 (SDF1) can interplay with CXC motif chemokine receptor type 4 (CXCR4) and activate AKT and ERK1/2 signaling pathways, resulting in antiapoptotic effects and contributing to tumor cell survival in CLL cells.^270^ In AML cells, CXCR4 activation induced by SDF1‐mediated leads to resistance to cytarabine through reducing miRNA let‐7a and facilitating transcriptional activation of MYC and Bcl‐xl.^271^ In CRC, CXCR4 is upregulated in chemoresistant tumor cells, and lymph node–derived stromal cells enhance resistance to oxaliplatin and 5‐FU via an SDF1/CXCR4 dependent manner.^272^

Exosomes, intraluminal vesicles of multivesicular bodies with a diameter of 30–100 nm, are ubiquitously present in most body fluids like blood, cerebrospinal fluids, urine, saliva, and lymphatic fluid.^273^, ^274^ Exosome contents not only reflect the composition of the donor cell but also mirror a regulated sorting mechanism.^275^ A complicated of multifarious proteins, including ECM proteins, enzymes, transcription factors, receptors, lipids, and nucleic acids (DNA, mRNA, and miRNA) inside and on the surface of the exosomes constitute their content.^276^, ^277^ Extensive evidence has shown that exosome‐mediated factors can promote tumorigenesis, metastasis, and therapeutic resistance of cancer cells via intercellular communication within TME.^273^, ^278^, ^279^ Tumor‐derived exosomes (TDEs) involve in the initiation, development, and progression of diverse tumor courses, including angiogenesis, TME remodeling, metastasis, and therapy resistance.^273^, ^279^, ^280^ TDEs induce the differentiation of various kinds of TME cells to CAFs that are the main cell population of TME in the majority of tumors; thus, exosomes play a vital role in ECM remodeling and TME reprogramming.^281^, ^282^ Exosomes derived from CAFs contain different molecules such as miRNAs and growth factors that have distinct influences on the target cells of TME. For example, the gemcitabine therapy of CAFs in pancreatic cancer stimulates the expression of miR‐146a and snail and prolonged exosome secretion, thereby promoting epithelial cell proliferation.^283^ Drug‐resistant tumor cells can pack the chemotherapeutic drugs in exosomes and shuttle therapeutic agents out of cancer cells.^283^ Besides, the delivery of exosomal cargo containing proteins, mRNA, and miRNA to cancerous cells is related to therapeutic resistance.^284^, ^285^ For instance, GBM cell–derived exosomes, which comprise MET‐protein and tyrosine phosphatase receptor type Z (PTPRZ1) fusion proteins, obtain temozolomide resistance via EMT.^286^ TDEs from gemcitabine‐resistant pancreatic cancer cells induce chemoresistance by trapping MRP5 and *P*‐gp or letting gemcitabine to flow back to TME.^287^ Exosomes derived from MCF7^WT^ breast cancer cells contain *P*‐gp and ubiquitin C‐terminal hydrolase‐L1 proteins that are able to induce doxorubicin resistance by elevating the level of *P*‐gp.^288^ TDEs promote platinum resistance by upregulating EMT markers and changing TGF‐β/SMAD signaling pathway in ovarian cancer cells, and exosomes from epithelial ovarian cancer A2780 platinum‐resistant cells attain resistance via promoting EMT.^289^ Exosome‐transmitted miR‐567 reverses trastuzumab resistance in breast tumor cells by inhibiting ATG5.^290^ In MDA‐MB‐231 and MCF‐7 breast cancer cells, exosome‐mediated miR‐155 induces chemoresistance via upregulating EMT markers and targeting CCAAT/enhancer‐binding protein β (C/EBP‐β), TGF‐β, and forkhead box O 3α (FOXO‐3α) mRNA.^291^ In HER2‐positive breast cancer, lncRNA‐SNHG14 induces exosome‐mediated trastuzumab resistance by targeting the apoptosis regulator Bcl‐2/BAX signaling.^292^ In NSCLC, exosome‐mediated miR‐21 delivery induces the upregulation of p‐AKT level, thereby resulting in increased gefitinib resistance.^293^ Exosomes containing miR‐32‐5p generate MDR in HCC cells by inhibiting PTEN, activating PI3K/AKT signaling cascade and promoting angiogenesis and EMT process.^294^ HCC‐derived exosomes containing miR‐221 induce sorafenib resistance through modulating apoptosis inhibition and caspase‐3 activity.^295^ Considering that exosomes are involved in various pathophysiological conditions, understanding the molecular mechanisms underlying exosome biogenesis and chemoresistance will benefit in developing novel therapeutics targeting exosome‐mediated tumorigenesis, progression, and chemoresistance.

One of the important functions of TDEs is to induce tumor vascular development. Angiogenesis, a multistep course by which cancers form new vasculature, is essential for tumor progression.^296^ Since Judah Folkman revealed the significant character of vascular networks for the proliferation and progression of solid tumors, establishing angiogenesis as a therapeutic target has become a focal aim.^297^ Up to now, antiangiogenic therapy has emerged as significant targeted therapeutic and diverse antiangiogenic agents are currently used in combination with other chemotherapeutic drugs for the treatment of various malignant tumors. Nowadays, over 11 antiangiogenic drugs have been approved by the U.S. FDA, including bevacizumab, aflibercept, sorafenib, ramucirumab, sunitinib, pazopanib, axitinib, vandetanib, Lenvatinib, and regorafenib.^298^ However, the treatment is in doubt on the grounds of sustainable efficacy, side effects, off‐target toxicities, and therapy resistance.^299^, ^300^ Clinical and experimental studies have illustrated that cancers employ compensatory/alternative/bypass angiogenic pathways and other adaptive mechanisms for their sustained growth, proliferation, and metastasis, after undergoing a therapy episode(s) with antiangiogenic drugs. That is, various compensatory signaling pathways driving tumor growth and metastasis invariably become the underlying cause of tumor refractoriness.^301^ Several preclinical and clinical studies have linked poor performance and resistance to antiangiogenic agents to the activation of a range of compensatory angiogenic signaling pathways and various angiogenic factors that support the angiogenic bypass mechanism.^302^ The vascular endothelial growth factor (VEGF)/VEGF receptor signaling pathway is the most promising angiogenic target due to its pivotal role in angiogenesis and tumor growth.^303^, ^304^ Previous studies have suggested that revascularization appears even after blocking VEGF signaling pathways because of the activation of redundant angiogenic pathways.

Serial evidence accumulated in recent years linked the function of various compensatory/alternative/bypass angiogenic canonical mechanisms sustaining the progression of cancers while exposed to antiangiogenic drugs.^299^, ^300^, ^305^ The current experimental, clinical, and epidemiological data has definitely outlined at least four potential different mechanisms, which can be considered for an explanation of evasive resistance to antiangiogenic therapies. The first mechanism refers to the activation and/or upregulation of compensatory pro‐angiogenic signaling pathways within the cancer.^306^ The second mechanism is mainly driven by myeloid/stromal cells, which compensate for the requirement of the VEGF‐mediated pathway, thereby promoting tumor angiogenesis.^305^ The third manner is attributed to the dual role of pericytes; first, in establishing the increased pericyte coverage of the tumor vasculature, and second, their potential angiogenic attributes, both serving as escape mechanisms from VEGF‐mediated angiogenesis.^307^ The fourth way is associated with remodeling and accessing normal vasculature for the invasion and metastasis of tumors in lieu of obligate neovascularization.^306^

As an important component of TME, the immune system plays a critical role in regulating the response of cancer cells to therapies, particularly immunotherapies. Immune‐targeted therapies approved for cancer include monoclonal antibodies against immune checkpoints like cytotoxic T‐lymphocyte‐associated protein 4 (CTLA4), PD‐1, and programmed cell death ligand 1 (PD‐L1), which aim to modulate the antitumor T‐cell immune response. The resistance to immune‐checkpoint inhibitors (ICIs) has been attributed to genomic and nongenomic mechanisms that are influenced by the tumor–host–microenvironment relationship.^308^ One mechanism is the upregulation of alternative immune checkpoint pathways. Koyama et al. revealed that, in mouse xenograft models with lung adenocarcinoma, other immune checkpoint proteins, including T‐cell immunoglobulin mucin 3, lymphocyte activation gene 3, and CTLA‐4, were upregulated after PD‐1 blockade therapy. The increased expression of alternative immune checkpoints in PD‐1 antibody‐bound T cells can inhibit T cell activity and confer resistance to PD‐1 blockade.^309^ Other mechanisms include the presence of immunosuppressive cells, such as regulatory T cells (Tregs) or myeloid‐derived suppressor cells (MDSCs), which can inhibit T cell function.^310^ Besides, the presence of stromal cells in TME, such as CAFs, has also been found to be associated with suppressive CD8^+^ T cell infiltration and insensitivity to αPD‐L1 antibody plus αCTLA‐4 antibody dual immune checkpoint blockade.^311^

Combination treatments appear to be the promising strategy to overcome resistance to immune checkpoint inhibitors. Combinations of ICIs with radiation, chemotherapy, targeted drug, or tumor vaccines have been proposed and tested. Radiation can trigger an adaptive mechanism to increase the expression of PD‐L1 on tumor cells.^312^ Additionally, radiation may increase the depth and duration of immune responses by promoting a more diverse adaptive antitumor immune response. As reported by Victor et al., the combination of radiation with dual checkpoint blockade (anti‐CTLA‐4 and anti‐PD‐1) can enhance tumor control and survival in a preclinical model of melanoma, where radiation prime immune response by increasing T cell receptor repertoire of intratumoral T cells and expanding peripheral clones.^313^ Combination with chemotherapy aims to assist ICI treatment by releasing neoantigens or modulating the TME via depleting Tregs and MDSCs.^314^ However, it has been reported that the efficacy of this combination therapy is model (cancer type)‐dependent and regimen‐type‐dependent. Combination with chemotherapeutic drugs is not always able to tune the TME or enhanced antitumor efficacy. Even with the effect of depleting MDSCs, the combination may not be beneficial to enhance treatment responsiveness.^314^ Additional studies combining single agents with ICIs are still required to better understand potential interactions between drugs and the heterogeneity of TME. Compared to the less predictable ICI–chemotherapy combination, the combination of TME‐targeted drugs with immune checkpoint blockade is considered a relatively logical approach to enhance the stimulation of antitumor immune response. TME‐targeted therapies, such as angiogenesis inhibitors, stromal cell depletion agents, and immunomodulatory agents, can modulate the TME and enhance the immune response against the tumor. Combining these agents with ICIs can lead to synergistic effects of blocking inhibitory signals in T cells and activating the immune response, thereby improving treatment outcomes.^315^ For example, the VEGF inhibitor bevacizumab can augment intratumoral CD8^+^ T cell infiltration through vascular normalization and endothelial cell activation, thereby potentiating PD‐L1 checkpoint inhibition with PD‐L1 inhibitor atezolizumab.^316^ This combination has shown promising anticancer efficacy and has been granted FDA approval for the treatment of patients with advanced unresectable or metastatic HCC.^317^

Combining oncolytic vaccines with ICIs is an emerging approach to overcome ICI resistance. Oncolytic vaccines are a type of oncolytic virus that are engineered to express tumor‐specific antigens, which can stimulate an immune response against the cancer cells.^318^ As ICI‐resistant tumors are with a suppressed immune microenvironment, the application of an oncolytic virus can reshape TME and assist ICIs to boost the immune system leading to a stronger antitumor response.^319^ This combination therapy has shown promising results in preclinical and clinical studies in a variety of cancers, including melanoma, breast cancer, and bladder cancer.^320^, ^321^, ^322^

Recent studies have shown that bacteria can reside within tumors and play a role in controlling cancer response to therapy by modulating the TME.^323^ For example, *Pseudomonas aeruginosa* and *Escherichia coli* are two types of bacteria found to colonize tumors in various types of cancer, including lung, breast, and pancreatic cancer.^324^, ^325^, ^326^ These bacteria can metabolize chemotherapy drugs such as gemcitabine, reducing their concentration within the tumor and rendering them less effective.^327^, ^328^ Moreover, certain commensal bacteria or pathogens within the TME may interact with immune cells and alter the production of cytokines and chemokines, which are involved in the immune response to cancer.^329^, ^330^ The influence of gut microbiota on cancer resistance has also gained extensive attention. A recent study found that in mice and patients with castration‐resistant prostate cancer, an adaptive change in the gut commensal microbiota was observed after ADT: There was an expansion of commensal microbiota species that can convert androgen precursors to produce androgens, which were absorbed into the systemic circulation thus inducing castration or endocrine therapy resistance.^331^ This type of resistance could be delayed by applying antibiotics to reduce gut microbiota or could be reversed by fecal microbiota transplantation hormone‐sensitive prostate cancer patients,^331^ implicating two possible ways to overcome bacterial‐mediated resistance. Although using antibiotics can directly eradicate the bacteria within the TME, this approach may have limitations due to the risk of antibiotic resistance.^328^ Another approach is to develop drug delivery systems specifically targeting the tumor cells while avoiding bacterial‐mediated metabolism.^332^, ^333^ For example, nanotechnology‐based drug delivery systems can be engineered to release drugs only in the tumor cells, minimizing the exposure of the drug to the bacteria.^323^, ^332^ The presence of bacteria within the TME and possible influences from gut microbiota are thought to be significant factors contributing to treatment resistance in cancer. Understanding the complex interactions between bacteria and tumors is essential for developing effective cancer treatments to overcome this resistance.

### 2.8 Tumor plasticity

CSCs are a small population of cells within a tumor that possess stem cell–like properties and are thought to be responsible for tumor initiation, growth, and metastasis.^334^, ^335^ These cells have been identified in various cancers, including breast, colon, and GBM. CSCs are less sensitive to chemotherapy and radiation therapy due to their ability to activate DNA damage repair and antiapoptotic signaling pathways, which may lead to incomplete tumor eradication and recurrence after treatment. Based on these findings, the researchers put forward the “CSC theory” and found evidence of the existence of CSCs in some cancer tissues.^336^, ^337^ CSCs can resist therapy mainly because of the overexpression of MDR transporters that mediate drug efflux, the more active DNA repair capacity, and the tendency to form new microvascular for the tumor.^338^ These characteristics enable CSCs to tolerate treatment, maintain the tumor with nutrients and oxygen, and rapidly repopulate the tumor. This mechanism is similar to that of normal tissue stem cells in response to trauma, which may explain that the bladder CSCs actively contribute to the cause of chemotherapy resistance after several cycles of chemotherapy.^339^, ^340^ Targeting this trauma response of CSCs can become a new therapeutic intervention. CSCs between different treatment cycles actively regenerate and respond to chemotherapy‐induced injury or apoptosis, much as normal tissue stem cells respond to trauma‐induced damage. Dying cells release a metabolite called prostaglandin E2 (PGE2) that stimulates proliferation and causes CSC to repopulate cancers shrunk by chemotherapy.^341^, ^342^ In normal cells, this is an integral part of the wound repair process, where PGE2 induces the regeneration of tissue stem cells. Ironically, PGE2 induces more CSC regeneration between chemotherapy cycles in cancers. An essential characteristic of CSC is maintaining self‐renewal ability, and the mechanism may be one key factor in promoting cancer development and metastasis.^343^, ^344^ CSC theory helps people understand the occurrence and development of cancers. In addition, CSCs can activate signaling pathways that promote cell survival and proliferation, such as the Wnt/β‐catenin and Notch pathways.^345^, ^346^, ^347^ Therefore, understanding the mechanisms underlying CSC‐mediated resistance to therapy is essential for developing effective cancer treatments. It provides a new perspective on cancer development, metastasis, drug resistance, and recurrence. Leukemia therapy targeting LSC (leukemia stem cells) has achieved good clinical efficacy, and beneficial research has also been carried out in solid cancers.^348^

EMT is one of the essential mechanisms of cancer metastasis. The latest research suggests that cancer cells with dual characteristics of EMT and stem cells are the key to cancer metastasis and drug resistance.^349^ There is a direct link between EMT and CSCs, but the molecular mechanism is still unclear. EMT phenomenon refers to a reversible process in which relatively stable epithelial cells lose cell polarity and intercellular adhesion and transform into spindle‐shaped mesenchymal cells with migration ability. It is ubiquitous in epithelial cancer cells. Its essential feature is the loss of the expression of epithelial markers, such as E‐cadherin, β‐catenin, tight junction protein, and epithelial cell adhesion molecules on the cell membrane of cancer cells,^350^, ^351^, ^352^, ^353^ and meanwhile obtaining a mesenchymal phenotype with the increased expression of vimentin, N‐cadherin, fibronectin, and β1 and β3 integrins.^354^, ^355^, ^356^ The mechanism of EMT is mainly due to changes in the epithelial cells themselves or the surrounding microenvironment, leading to the activation of a series of signal transduction pathways, and related transcription factors in the nucleus play a regulatory role.^357^ Precise intracellular signaling mechanisms regulate different degrees of epithelial cell transformation. Various extracellular signals activate different nuclear transcription factors by binding to specific receptors on the cell surface. The common feature of these transcription factors is that they can recognize the DNA binding sequence of the E‐box motif on the target gene promoter, thereby regulating the expression of the target gene and initiating EMT.^358^ Loss of E‐cadherin expression is currently considered the most prominent feature of EMT. Decreased E‐cadherin levels can lead to decreased cell adhesion and make cells easily invade and metastasize.^351^ Cancer cells lose some of the characteristics of epithelial cells and acquire the characteristics of mesenchymal cells through EMT, so cancer cells can obtain stronger invasion and migration capabilities.^359^, ^360^

Snail is involved in triggering EMT during the progression of epithelial cancers and is a crucial point in the occurrence of EMT. Snail expression correlates with the reduced loss of E‐cadherin and acts as a direct repressor of E‐cadherin transcription.^361^ Twist is another essential transcription factor that regulates EMT. Its mechanism of inducing EMT is to directly or indirectly bind the E‐cadherin promoter through the E‐box motif, thereby inhibiting the expression of E‐cadherin.^362^ Snail/Twist1 knockout breast cancer model demonstrates that chemotherapy resistance is associated with EMT.^363^ Studies have shown that EMT may be involved in drug resistance of cancer cells, such as lung, pancreatic, and breast cancer.^364^, ^365^, ^366^ Numerous studies have shown that cancer cells develop resistance to carboplatin or paclitaxel by acquiring mesenchymal cell phenotype, indicating that EMT may be the instigator of chemotherapy resistance.^367^, ^368^ The latest research shows that cancers may have a type of partial EMT, which means the cancer cells have both epithelial and mesenchymal cell phenotypes, and these cancer cells are more aggressive than the others.^368^ EMT has been described as a significant cause of EGFRi failure in EGFR‐mutated NSCLC.^369^ Cellular transdifferentiation, a phenotypic shift from adenocarcinoma to squamous cell carcinoma or neuroendocrine carcinoma, occurs in 3%–14% of EGFR‐mutant NSCLC patients, and approximately 17% of prostate cancer failed with abiraterone/enzalutamide therapy.^370^

From the discussion above, it is evident that targeting EMT processes or cellular plasticity has great potential to circumvent drug resistance. However, only a few compounds adesigned to inhibit the EMT process are currently in clinical trials. Inhibitors targeting Notch, TGF‐β, and Wnt signaling pathways are also promising candidates for inhibiting EMT and cellular plasticity.^371^ TGF‐β heterogeneity in the cancer microenvironment creates rapidly dividing CSCs that accelerate cancer growth and others that invade surrounding healthy tissue and evade treatment.^372^, ^373^ For example, PF‐03446962 and galunisertib are antagonists designed to inhibit TGF‐β receptors, currently in phase I clinical studies in solid cancers (NCT00557856, NCT02423343).^374^ Both PF‐03446962 and galunisertib inhibit the EMT program, thereby preventing cancer development. In addition, Wnt inhibitors such as ETC‐1922159 and OMP‐54F28 have been reported to inhibit the EMT program and are currently in phase I clinical trials in cancer (NCT02521844, NCT01608867).^375^

## 3 PERSPECTIVES IN OVERCOMING CANCER RESISTANCE

Cancer chemotherapy is still a required treatment method for most cancer patients. However, cancer cells are prone to MDR, a significant problem limiting the efficacy of current chemotherapeutic drugs lacking selectivity. Many cancer patients have a remarkable curative effect in the early stage of chemotherapy. However, the drug resistance of cancer cells increases with the prolongation of treatment time, eventually leading to treatment failure. Anticancer strategies are constantly evolving, and targeted therapy drugs have encountered increasing resistance after the initial excitement, including primary and acquired resistance. Strategies to address drug resistance through combination therapy have made some progress, although considerable challenges have been faced, and the full potential of combination therapy has not yet been realized.

According to statistics, in the early 1990s, the main reasons for the failure of new drug development were concentrated in poor pharmacokinetics and limited biological activity.^376^, ^377^ With the introduction of absorption, distribution, metabolism, and excretion for predictive analysis and research applications, the clinical failure rate dropped from 40% to 10% in 2000.^378^ With the accumulation of basic research on targets and signaling pathways and the advent of the era of genome sequences, the success rate of clinical development of targeted drugs is also increasing. For example, the familiar TKIs have become one of the most popular research directions. In recent years, with a series of significant breakthroughs in technological applications, such as the application of high‐throughput genomics to discover new targets,^379^, ^380^ the identification of molecular biomarkers,^381^, ^382^ and the progress of statistical methods in biological and chemoinformatics,^383^, ^384^ the progress and the success rate of research and development have been greatly improved. However, new problems are still emerging, with lacking clinical efficacy and increased toxicity still the main directions of failure in the second research and development phase.

Nanodrugs represent a promising approach to overcoming resistance to cancer. These drugs are engineered to be nanoscale size and have unique physicochemical properties that enable them to target cancer cells while minimizing off‐target effects.^385^ Nanodrugs can be designed to overcome resistance by delivering drugs to cancer cells in more targeted and controlled manner and bypassing resistance mechanisms such as efflux pumps and DNA repair pathways.^386^, ^387^, ^388^ One key advantage of nanodrugs is their ability to improve the pharmacokinetics and pharmacodynamics of drugs.^386^ By encapsulating drugs within nanocarriers, the drug concentration within the tumor can be increased, improving drug efficacy.^332^ Additionally, nanocarriers can protect the drug from degradation and clearance, leading to a longer half‐life and a sustained drug release.^386^, ^389^ Nanodrugs can also be engineered to overcome specific resistance mechanisms. For example, nanodrugs can target CSCs by incorporating specific ligands that bind to stem cell markers.^390^ Nanodrugs can also be engineered to overcome bacterial‐mediated resistance by releasing drugs only within the TME and avoiding bacterial exposure.^328^ By improving drug delivery, targeting, and overcoming specific resistance mechanisms, nanodrugs can potentially improve the efficacy of cancer treatment and patient outcomes.^391^ However, further research is needed to optimize the design and delivery of nanodrugs and to ensure their safety and efficacy in clinical use.

Polytherapy can make cancer cells less likely to develop compensatory resistance mechanisms than a single or sequential drug regimen. There are many problems in the reversal of MDR with combination drugs, including clinical toxicity and pharmacokinetic interactions. The therapeutic range of each drug may be narrow when administered in combination, and there may be an overlap in toxicity.^392^ In some cases, in addition to the expected overlapping toxicities, combination therapy has some characteristic toxicities.^393^, ^394^ We cannot accurately predict toxicity from preclinical models, which adds to the challenge of optimizing the toxicity–potency balance of drug combinations. Meanwhile, the pharmacokinetic interaction is the question of how to combine two or more drugs.^395^, ^396^ For example, lapatinib is a substrate and moderate inhibitor of CYP450‐3A4 and substantially reduces the clearance of drugs such as other CYP450‐3A4 substrates.^397^ In the phase I clinical trial of lapatinib and pazopanib, investigators compared the historical pharmacokinetic parameters of the two drugs and concluded that the drug combination did not alter drug exposure.^398^ However, in a more detailed phase II pharmacokinetic analysis, the combination indicated a significant drug–drug interaction contributing to the reduced efficacy of lapatinib in glioma patients.^399^, ^400^ In addition, the frequent use of antiepileptic drugs in this patient population also reduced pazopanib exposure, resulting in poorer patient outcomes.^401^ This highlights the importance of detailed pharmacokinetic assessment for the combined evaluation of anticancer drugs. Combination therapies approved or in clinical trials in the recent 5 years are summarized in Table 1.

**TABLE 1:** Cancer combination therapies approved or in clinical trials in the recent 5 years.

Combination therapy	Target cancer type	Approval date/Clinical trials process
Tremelimumab + durvalumab + platinum‐based chemotherapy	Adult patients with metastatic NSCLC with no sensitizing EGFR mutation or ALK genomic cancer aberrations	November 10, 2022 (NCT03164616)
Brentuximab vedotin + doxorubicin + vincristine + etoposide + prednisone + cyclophosphamide	Pediatric patients 2 years of age and older with previously untreated high risk cHL	November 10, 2022 (NCT03755804)
Cemiplimab‐rwlc + platinum‐based chemotherapy	Adult patients with advanced NSCLC with no EGFR, ALK, or ROS1 aberrations	November 8, 2022 (NCT03409614)
Tremelimumab + durvalumab	Adult patients with uHCC	October 21, 2022 (NCT03298451)
Durvalumab + gemcitabine + cisplatin	Adult patients with locally advanced or metastatic BTC	September 2, 2022 (NCT03875235)
Darolutamide + docetaxel	Adult patients with mHSPC	August 5, 2022 (NCT02799602)
Dabrafenib + trametinib	Adult and pediatric patients ≥6 years of age with unresectable or metastatic solid cancers with BRAF V600E mutation	Accelerated Approval June 22, 2022 (NCT02034110, NCT02465060, NCT02124772, original projected completion: October 21, 2028)
Nivolumab + fluoropyrimidine‐ and platinum‐based chemotherapy	Patients with advanced or metastatic ESCC	May 27, 2022 (NCT03143153)
Nivolumab + ipilimumab	Patients with advanced or metastatic ESCC	May 27, 2022 (NCT03143153)
Ivosidenib + azacitidine (azacitidine for injection)	Newly diagnosed AML with a susceptible IDH1 mutation	May 25, 2022 (NCT03173248)
Nivolumab + relatlimab‐rmbw	Adult and pediatric patients 12 years of age or older with unresectable or metastatic melanoma	March 18, 2022 (NCT03470922)
Abatacept + a calcineurin inhibitor + methotrexate	Adults and pediatric patients 2 years of age and older undergoing HSCT	December 15, 2022 (NCT 01743131)
Rituximab + chemotherapy	Pediatric patients (≥6 months to <18 years) with previously untreated, advanced stage, CD20‐positive DLBCL, BL, BLL, or mature B‐AL	December 2, 2022 (NCT01516580)
Pembrolizumab + chemotherapy, with or without bevacizumab	Patients with persistent, recurrent, or metastatic cervical cancer whose cancers express PD‐L1	October 13, 2021 (NCT03635567)
Lenvatinib + pembrolizumab	Adult patients with advanced RCC	August 10, 2021 (NCT02811861)
Pembrolizumab + chemotherapy as neoadjuvant treatment, and then continued as a single agent as adjuvant treatment after surgery	High‐risk, early‐stage, TNBC	July 26, 2021 (NCT03036488)
Pembrolizumab + lenvatinib	Patients with advanced endometrial carcinoma	July 21, 2021 (NCT03517449)
Daratumumab + hyaluronidase‐fihj + pomalidomide + dexamethasone	Adult patients with multiple myeloma	July 9, 2022 (NCT03180736)
Pembrolizumab + trastuzumab + fluoropyrimidine‐ and platinum‐containing chemotherapy	Patients with locally advanced unresectable or metastatic HER2‐positive gastric or GEJ adenocarcinoma	Accelerated Approval May 5, 2021 (NCT03615326, original projected completion: September 30, 2024)
Nivolumab + fluoropyrimidine‐ and platinum‐containing chemotherapy	Advanced or metastatic gastric cancer, GEJ, and esophageal adenocarcinoma	April 16, 2021 (NCT02872116)
Isatuximab‐irfc + carfilzomib + dexamethasone	Adult patients with relapsed or refractory multiple myeloma	March 31, 2021 (NCT03275285)
Pembrolizumab + platinum and fluoropyrimidine‐based chemotherapy	Patients with metastatic or locally advanced GEJ carcinoma	March 22, 2021 (NCT03189719)
Melphalan flufenamide + dexamethasone	Adult patients with relapsed or refractory multiple myeloma	Accelerated Approval February 26, 2021 (NCT02963493, original projected completion: February 28, 2022)
Nivolumab + cabozantinib	Patients with advanced RCC	January 22, 2021 (NCT03141177)
Selinexor + bortezomib + dexamethasone	Adult patients with multiple myeloma	December 18, 2020 (NCT03110562)
Margetuximab‐cmkb + chemotherapy	Adult patients with metastatic HER2‐positive breast cancer	December 16, 2020 (NCT02492711)
Naxitamab + granulocyte‐macrophage colony‐stimulating factor	Pediatric patients 1 year of age and older and adult patients with relapsed or refractory high‐risk neuroblastoma in the bone or bone marrow	Accelerated Approval November 25, 2020 (NCT 03363373, original projected completion: September 30, 2027)
Pembrolizumab + chemotherapy	Patients with locally recurrent unresectable or metastatic TNBC	November 13, 2020 (NCT02819518)
Venetoclax + azacitidine + decitabine	Newly diagnosed AML in adults 75 years or older	October 16, 2020 (NCT02993523)
Nivolumab + ipilimumab	Adult patients with unresectable malignant pleural mesothelioma	October 2, 2020 (NCT02899299)
Carfilzomib + daratumumab + dexamethasone	Adult patients with relapsed or refractory multiple myeloma	August 20,2020 (NCT03158688)
Tafasitamab‐cxix + lenalidomide	Adult patients with relapsed or refractory DLBCL	Accelerated Approval July 31, 2020 (NCT02399085, original projected completion: December 31, 2025)
Atezolizumab + cobimetinib + vemurafenib	Patients with BRAF V600 mutation‐positive unresectable or metastatic melanoma	July 30, 2020 (NCT02908672)
Oral decitabine + cedazuridine	Adult patients with MDS	July 7, 2020 (NCT02103478)
Pertuzumab + trastuzumab + hyaluronidase–zzxf	Patients with HER2‐positive, locally advanced, inflammatory, or early stage breast cancer	June 29, 2020 (NCT03493854)
Ramucirumab + erlotinib	Metastatic NSCLC with EGFR exon 19 deletions or exon 21 mutations	May 29, 2020 (NCT02411448)
Atezolizumab + bevacizumab	Patients with unresectable or metastatic hepatocellular carcinoma	May 29, 2020 (NCT03434379)
Nivolumab + ipilimumab + 2 cycles of platinum‐doublet chemotherapy	Patients with metastatic or recurrent NSCLC	May 26, 2020 (NCT03215706)
Olaparib + bevacizumab	Adult patients with advanced epithelial ovarian, fallopian tube, or primary peritoneal cancer	May 8, 2020 (NCT03737643)
Ibrutinib + rituximab	Adult patients with CLL or SLL	April 21, 2020 (NCT02048813)
Tucatinib + trastuzumab + capecitabine	Adult patients with advanced unresectable or metastatic HER2‐positive breast cancer	April 17, 2020 (NCT02614794)
Encorafenib + cetuximab	Adult patients with metastatic CRC with a BRAF V600E mutation	April 8, 2020 (NCT02928224)
Durvalumab + etoposide + either carboplatin or cisplatin	Patients with ES‐SCLC	March 27, 2020 (NCT03043872)
Nivolumab + ipilimumab	Patients with HCC	Accelerated Approval March 10, 2020 (NCT01658878, original projected completion: July 31, 2024)
Isatuximab‐irfc + pomalidomide + dexamethasone	Adult patients with multiple myeloma	March 2, 2020 (NCT02990338)
Neratinib + capecitabine	Adult patients with advanced or metastatic HER2‐positive breast cancer	February 25, 2020 (NCT01808573)
Atezolizumab + paclitaxel protein‐bound + carboplatin	Adult patients with metastatic NSCLC	December 3, 2019 (NCT02367781)
Daratumumab + bortezomib + thalidomide + dexamethasone	Adult patients with multiple myeloma in newly diagnosed	September 26, 2019 (NCT02541383)
Pembrolizumab + lenvatinib	Patients with advanced endometrial carcinoma	September 17, 2019 (NCT02501096)
Selinexor + dexamethasone	Adult patients with RRMM	July 3, 2019 (NCT02336815)
Daratumumab + lenalidomide + dexamethasone	Patients with newly diagnosed multiple myeloma	June 27, 2019 (NCT02252172)
Polatuzumab vedotin‐piiq + bendamustine + a rituximab product	Adult patients with relapsed or refractory DLBCL	Accelerated Approval June 10, 2019 (NCT02257567, original projected completion: June 30, 2024)
Lenalidomide + a rituximab product	Previously treated FL and previously treated MZL	May 28, 2019 (NCT01996865)
Alpelisib + fulvestrant	Postmenopausal women, and men, with HR‐positive, HER2‐negative, PIK3CA‐mutated, advanced or metastatic breast cancer	May 24, 2019 (NCT02437318)
Avelumab + axitinib	Patients with advanced RCC	May 14, 2019 (NCT02684006)
Atezolizumab + carboplatin + etoposide	Adult patients with ES‐SCLC	March 18, 2019 (NCT02763579)
Glasdegib + low‐dose cytarabine	Newly diagnosed AML in patients who are 75 years old or older	November 21, 2018 (NCT01546038)
Brentuximab vedotin + chemotherapy	Previously untreated sALCL or other PTCL	November 16, 2018 (NCT01777152)
Pembrolizumab + carboplatin + either paclitaxel or nab‐paclitaxel	Metastatic squamous NSCLC	October 30, 2018 (NCT02775435)
Pembrolizumab + pemetrexed + platinum	Patients with metastatic, non‐squamous NSCLC	August 20, 2018 (NCT02578680)
Ribociclib + an aromatase inhibitor	Pre/perimenopausal women with HR‐positive, HER2‐negative advanced or metastatic breast cancer	July 18, 2018 (NCT02278120)
Ribociclib + fulvestrant	Postmenopausal women with HR‐positive, HER2‐negative advanced or metastatic breast cancer	July 18, 2018 (NCT02278120)
Ipilimumab + nivolumab	Patients 12 years of age and older with metastatic CRC	Accelerated Approval July 10, 2018 (NCT02060188, original projected completion: July 31, 2024)
Encorafenib + binimetinib	Patients with unresectable or metastatic melanoma with a BRAF V600E or V600K mutation	June 27, 2018 (NCT01909453)
Bevacizumab + carboplatin + paclitaxel	Patients with epithelial ovarian, fallopian tube, or primary peritoneal cancer	June 13, 2018 (NCT00262847)
Dabrafenib + trametinib for the adjuvant treatment	Patients with melanoma with BRAF V600E or V600K mutations	April 30, 2018 (NCT01682083)
Nivolumab + ipilimumab	Previously untreated advanced renal cell carcinoma	April 16, 2018 (NCT02231749)
Abiraterone acetate tablets + prednisone	Metastatic high‐risk castration‐sensitive prostate cancer	February 7, 2018 (NCT01715285)
Pertuzumab + trastuzumab + chemotherapy	Patients with HER2‐positive early breast cancer	December 20, 2017 (NCT01358877)
Dabrafenib + trametinib	Patients with metastatic NSCLC with BRAF V600E mutation	June 22, 2017 (NCT01336634)

Abbreviations: ALK, anaplastic lymphoma kinase; AML, acute myeloid leukemia; B‐AL, B‐cell acute leukemia; BL, Burkitt's lymphoma; BLL, Burkitt‐like lymphoma; BRAF, B‐raf proto‐oncogene; BTC, biliary tract cancer; cHL, classic Hodgkin lymphoma; CLL, chronic lymphocytic leukemia; CRC, colorectal cancer; DLBCL, diffuse large B cell lymphoma; EGFR, epidermal growth factor receptor; ESCC, esophageal squamous cell carcinoma; ES‐SCLC, extensive‐stage small cell lung cancer; GEJ, gastroesophageal junction adenocarcinoma; HER2, human epidermal growth factor receptor 2; HSCT, hematopoietic stem cell transplantation; IDH1, isocitrate dehydrogenase 1; MDS, myelodysplastic syndromes; mHSPC, metastatic hormone‐sensitive prostate cancer; MZL, marginal zone lymphoma; NSCLC, non‐small cell lung cancer; PD‐L1, programmed cell death ligand 1; PIK3CA, phosphatidylinositol‐4,5‐bisphosphate 3‐kinase catalytic subunit alpha; PTCL, peripheral T‐cell lymphoma; RCC, renal cell carcinoma; ROS1, proto‐oncogene 1; RRMM, relapsed or refractory multiple myeloma; sALCL, systemic anaplastic large cell lymphoma; SLL, small lymphocytic lymphoma; TNBC, triple‐negative breast cancer; uHCC, unresectable hepatocellular carcinoma.

*Sources*: Data from ClinicalTrials.gov.

Antibodies, cytotoxic drugs, and conjugates bridge ADC (antibody–drug–conjugates) drugs. The antibody will specifically recognize and guide the drug to the lesion. The conjugate can generally be broken in the pH environment of the lesion site, thereby releasing the cytotoxin with a therapeutic effect. Cytotoxins mainly target the DNA and tubulin of cancer cells, block the proliferation of tumor cells, and induce apoptosis.^401^, ^402^ After the second new ADC drug Adcetris (brentuximab vedotin) was approved by the FDA in 2011, 10 ADC drugs have been approved so far, 7 of which have been approved in the last 3–4 years. ADC drugs can improve the selectivity of tumor treatment and can better deal with the drug resistance of targeted monoclonal antibodies.^403^, ^404^ It is considered one of the essential directions for developing monoclonal antibodies (especially in the field of tumor‐targeted therapy) in the next decade. The tissue specificity and cytotoxicity of the new generation of ADCs have been improved compared with the previous generation products, allowing them to show fantastic activity in treating refractory cancers.^405^, ^406^ The currently available evidence shows that the potency of ADCs is based on complex and delicate interactions among antibodies, conjugates, various components of the cytotoxin, and the tumor and its microenvironment.^407^ Combining ADCs with other antibody therapies, such as ADCs with the anti‐VEGF monoclonal antibody bevacizumab, has also shown activity in preclinical models.^408^ This may be due to bevacizumab's enhanced drug delivery efficiency by altering tumor vascular innervation.

Some researchers proposed to reverse chemotherapy resistance by targeting inhibitor of apoptosis (IAP) inhibitors.^409^ The IAP protein family plays an essential role in controlling programmed cell death, and the expression level of IAP in cancer cells is significantly increased. Therefore, by directly or indirectly regulating the expression of apoptotic proteins on the extrinsic apoptotic pathway (transmembrane apoptotic pathway)^410^ and the intrinsic apoptotic pathway (mitochondrial apoptotic pathway),^411^ the chemosensitivity of cell apoptosis can be improved and obtained a potential response. However, the current combination of this method and chemotherapy only targets a small number of tumor cells, and there are cases where tumor cells are resistant to IAP inhibitors.^412^

The technologies for reversing tumor MDR at the gene level mainly include antisense nucleic acid technology,^413^ ribozyme technology,^414^ and RNA interference (RNAi) technology.^415^, ^416^ Drugs that regulate miRNA expression (such as miR‐125, −20, −24) may have specific clinical application prospects in reversing tumor MDR.^417^, ^418^ Although many constructed‐cell line banks exist, the genetic characterization of these cells and human tumor samples varies enormously.^419^ Researchers are attempting to create patient‐derived cell culture models and then test these models on pharmacogenomics platforms to rapidly discover multiple effective drug combinations.^418^, ^419^ These models can better reflect the biological complexity of human drug‐resistant tumor cells than constructed cell models. However, the combination therapy results still need to be verified by randomized clinical trials.

The study of functional drug delivery systems that can reverse tumor MDR will have broad application prospects in improving the efficacy of chemotherapy drugs and reducing side effects.^395^ Due to tumors’ heterogeneity and drug resistance, it is usually challenging to achieve an excellent therapeutic effect using one drug alone. Therefore, people have been devoting themselves to designing nanocarriers that can be loaded with multiple anticancer drugs and improve the therapeutic effect through synergistic therapeutic effects.^414^, ^420^ Combining chemotherapeutics and MDR‐reversing agents using a drug delivery system has been a promising strategy for reversing MDR in recent years.^421^, ^422^, ^423^ Common nano‐drug carriers that have been reported to be used for the co‐delivery of drugs include liposomes, nanoparticles, micelles, nano‐emulsions, and nanogels.^424^ Combining MDR drug reversal agents, RNAi/DNA, targeted drugs with nanocarrier drug delivery systems, through nonspecific internalization, reduce the efflux of drugs by ABC transporters in tumor cells, increases the uptake of drugs, and through RNAi/DNA Delivery, active targeting, and increased responsiveness to physiological stimuli can reverse tumor cell MDR.^425^, ^426^, ^427^ Combination types of drugs delivered in combination include chemotherapeutics and chemotherapeutics,^428^, ^429^ chemotherapeutics and MDR reversal agents,^424^, ^430^, ^431^ chemotherapy drugs and siRNA,^432^, ^433^, ^434^ chemotherapy drugs and monoclonal antibodies.^425^, ^435^ Among them, combining chemotherapy and other drugs is the most common type of combination administration.^423^ The rapid development of nano‐drug delivery carriers provides a good carrier platform for improving the therapeutic effect of drugs and overcoming the MDR problem of tumors. Combining drug administration with nano‐drug carriers can enhance the effect of reversing MDR through various forms and achieve a substantial combined effect by co‐delivering different types of drugs. Nano‐drug carrier‐mediated co‐administration is a promising strategy for reversing tumor MDR. Currently, there is more and more research on reversing MDR of tumors by combined administration of nano‐drug carriers.^419^, ^436^ With the clinical application of nanocarrier drug delivery system, this technology shows great potential in reversing tumor MDR.

Tumor immunotherapy can improve tumor patients’ immune function, kill or inhibit tumor cells, and reverse tumor MDR with little adverse reactions. There are many studies on tumor immunohistology, enzymology, and cytokines,^437^, ^438^ but most are still experimental research. Among them, the systems biology approach involves the analysis of large amounts of data, including genetics, transcriptomics, proteomics, or factors affecting posttranslational regulation. Some systems’ biology approaches study the importance of proteins in intracellular signaling networks and explore the possibility of reversing tumor drug resistance based on the differential effects of drug targets on intracellular associations.^439^

**FIGURE 4:**
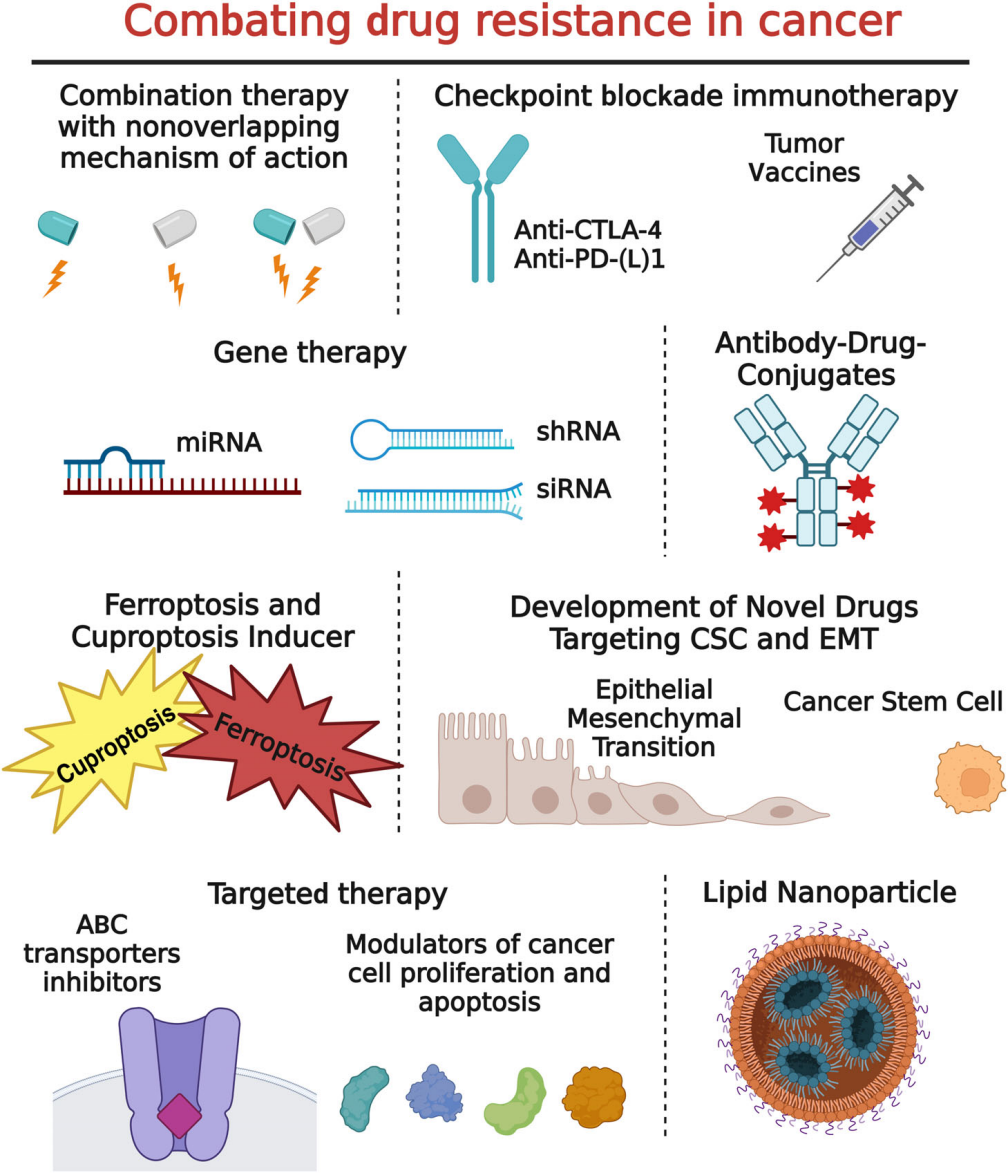
Overcoming drug resistance in cancer. The purpose of overcoming the drug resistance of cancer cells is to optimize the sensitivity of the therapy. This can be achieved by polytherapy using the combination of at least two drugs; immunotherapy using checkpoint inhibitors or monoclonal antibodies; antibody–drug–conjugates improving the selectivity of cancer treatment; gene technology modifying the epigenetic sequence; targeted therapy targeting the overexpression of drug efflux transporter or vital proteins for the cancer cell apoptosis; and nanoparticle delivery system improving the efficacy of the drug and reducing the side effect. *Source*: This figure was created with Biorender.com.

The new technologies, for example, the novel computational method, can be used for predicting polytherapy switching strategies to overcome tumor heterogeneity and evolution. In addition, the widely used gene silencing tools, including shRNA and CRISPR, could help researchers propose and predict effective drug effects both in vitro and in vivo. Finally, the clinical trial designs that reflect drug toxicity and utilize intermittent doses and adaptive trial designs that give dynamic combinations after the emergence of resistance will significantly accelerate the development of more effective therapeutic combinations to reverse the MDR in cancer cells (Figure 4).

## 4 CONCLUSION

As summarized in Table 2, MDR in cancer is a multifactorial phenomenon that results in drug inactivation, efflux, target alteration, cancer cell death inhibition, DNA damage repair, cellular heterogeneity, and more. Tumor drug resistance has become a significant problem in oncology, affecting the treatment effect and prognosis of cancer patients, and may lead to tumor progression or even recurrence. Therefore, it becomes crucial to understand the causes and underlying mechanisms of cancer drug resistance, which will facilitate the development of various therapies or combinations for treating different cancers. Combination therapy is considered the most important treatment option for personalized medicine and overcoming MDR. Simultaneous use of two or more treatment methods can effectively overcome MDR, avoid clinical toxicity, and improve patients’ survival rate and quality of life. The specificity of cancer cells is a promising research and therapeutic area, which will help to develop tumor‐targeted therapies with low toxicity to normal cells and higher tumor specificity.

**TABLE 2:** Typical resistance mechanisms in cancer and combating strategies.

Resistance mechanisms	Combating strategies	Typical example
Drug inactivation	Drug replacement or combination with enzyme enhancer	Nucleoside analog phosphate prodrugs could bypass DCK deficiency‐mediated AraC resistance in AML without the demand for DCK phosphorylation^36^ or combination with etoposide‐enhancing DCK activity^39^
Drug detoxification	Combination with metabolism enzyme inhibitor	GSTP1‐1 inhibitor MC3181 could be used to reverse the vemurafenib resistance in melanoma^80^, ^81^
Reduced drug uptake	Modulation of the cell membrane lipid composition to increase permeability and fluidity	Cancer cell membrane–based biomimetic nanoparticle delivery systems could improve therapeutic efficacy for various MDR cancers^103^, ^104^, ^105^
Increased drug efflux	Drug replacement, combination with ABC transporter inhibitor, or silence of the efflux transporters	Targeted anticancer drugs, such as tepotinib and poziotinib, could inhibit ABCB1 and ABCG2 to reverse the resistance to ABCB1 and ABCG2 substrates^128^, ^129^, ^130^
Mutation of drug targets	Development of new generation or multitarget anticancer drug	EAI045 is the first allosteric EGFR TKI designed to overcome the acquired resistance mediated by T790M and C797S mutations^147^
DNA damage repair	Combination with DNA repair inhibitor	ERK or p38 kinase inhibitors could overcome the resistance mediated by 5‐FU‐induced ERCC1 overexpression^198^
Blocked apoptosis	Combination with apoptosis inducer	Navitoclax could trigger the resistance in CLL by inhibiting Bcl‐2 to induce apoptosis^219^
Autophagy induction	Combination with autophagy modulators	Imatinib combined with siRNAs targeting ATGs could potentiate the cytotoxicity and reverse the acquired resistance in both GIST and CML^233^
Cell death inhibition	Ferroptosis and cuproptosis induction	Cellular ferroptosis and cuproptosis could sensitize chemotherapy resistance in various cancers^239^, ^240^, ^241^, ^242^
Tumor microenvironment	Combination with inhibitors targeting the specific protein function in TME	Inhibition of the β1 integrin activity by monoclonal antibody AIIB2 could promote radiotherapy efficacy and elevates sensitivity to HER2‐targeting agents of breast cancer cells^265^, ^266^
Cancer stem cell	Development of novel drugs targeting cancer stem cells	PF‐03446962 is designed to inhibit TGF‐β receptors resulting in the antagonization of CSC division^374^
Epithelial‐to‐mesenchymal transition	Development of novel drugs targeting the EMT process	Inhibitors targeting Notch, TGF‐β, and Wnt signaling pathways are promising candidates for inhibiting EMT^371^
Immunotherapy and immune responses	Combination with radiation, chemotherapy, targeted drug, or tumor vaccines	VEGF inhibitor bevacizumab can augment intratumoral CD8^+^ T cell infiltration and endothelial cell activation, thereby potentiating PD‐L1 checkpoint inhibition with PD‐L1 inhibitor atezolizumab^316^, ^317^

Abbreviations: 5‐FU, 5‐fluorouracil; ABC, ATP‐binding cassette; ATG, autophagy‐related gene; Bcl‐2, B‐cell lymphoma 2; CLL, chronic lymphocytic leukemia; CML, chronic myeloid leukemia; CSC, cancer stem cell; DCK, deoxycytidine kinase; EGFR, epidermal growth factor receptor; EMT, epithelial–mesenchymal transition; ERCC, excision repair cross‐complementing 1; ERK, extracellular signal‐regulated kinase; GIST, gastrointestinal stromal tumors; GSTP1, glutathione‐S‐transferase Pi 1; HER2, human epidermal growth factor receptor 2; MDR, multidrug resistance; PD‐L1, programmed cell death ligand 1; TGF‐β, transforming growth factor beta; TKI, tyrosine kinase inhibitor; TME, tumor microenvironment; VEGF, vascular endothelial growth factor.

## AUTHOR CONTRIBUTIONS

Zhe‐Sheng Chen, Yihang Pan, and Leli Zeng designed the review. Zi‐Ning Lei, Qin Tian, and Qiu‐Xu Teng did the literature search and wrote the manuscript. Zi‐Ning Lei and Qiu‐Xu Teng prepared the table and figures. Zhe‐Sheng Chen, Yihang Pan, and John N. D. Wurpel reviewed and revised the manuscript. All authors listed have made a substantial contribution to the work. All authors have read and approved the article.

## CONFLICT OF INTEREST STATEMENT

The authors declare no conflict of interest. Author Zhe‐Sheng Chen is the Editorial Board Member of MedComm. Author Zhe‐Sheng Chen was not involved in the journal's review of, or decisions related to, this manuscript.

## EtHICS STATEMENT

Not applicable.

## Data Availability

Not applicable.

## References

[1] Bray F , Laversanne M , Weiderpass E , Soerjomataram I . The ever‐increasing importance of cancer as a leading cause of premature death worldwide. Cancer. 2021;127(16):3029‐3030. 34086348 10.1002/cncr.33587

[2] Sung H , Ferlay J , Siegel RL , et al. Global cancer statistics 2020: GLOBOCAN estimates of incidence and mortality worldwide for 36 cancers in 185 countries. CA Cancer J Clin. 2021;71(3):209‐249. 33538338 10.3322/caac.21660

[3] Awan A , Esfahani K . Endocrine therapy for breast cancer in the primary care setting. Curr Oncol. 2018;25(4):285‐291. 30111969 10.3747/co.25.4139 PMC6092062

[4] Kruger S , Ilmer M , Kobold S , et al. Advances in cancer immunotherapy 2019 – latest trends. J Exp Clin Cancer Res. 2019;38(1):268. 31217020 10.1186/s13046-019-1266-0 PMC6585101

[5] Kareliotis G , Tremi I , Kaitatzi M , et al. Combined radiation strategies for novel and enhanced cancer treatment. Int J Radiat Biol. 2020;96(9):1087‐1103. 32602416 10.1080/09553002.2020.1787544

[6] Bedard PL , Hyman DM , Davids MS , Siu LL . Small molecules, big impact: 20 years of targeted therapy in oncology. Lancet. 2020;395(10229):1078‐1088. 32222192 10.1016/S0140-6736(20)30164-1

[7] Szostakowska M , Trebinska‐Stryjewska A , Grzybowska EA , Fabisiewicz A . Resistance to endocrine therapy in breast cancer: molecular mechanisms and future goals. Breast Cancer Res Treat. 2019;173(3):489‐497. 30382472 10.1007/s10549-018-5023-4 PMC6394602

[8] Bukowski K , Kciuk M , Kontek R . Mechanisms of multidrug resistance in cancer chemotherapy. Int J Mol Sci. 2020;21(9):3233. 32370233 10.3390/ijms21093233 PMC7247559

[9] Bueschbell B , Caniceiro AB , Suzano PMS , Machuqueiro M , Rosario‐Ferreira N , Moreira IS . Network biology and artificial intelligence drive the understanding of the multidrug resistance phenotype in cancer. Drug Resist Updat. 2022;60:100811. 35121338 10.1016/j.drup.2022.100811

[10] Turajlic S , Furney SJ , Stamp G , et al. Whole‐genome sequencing reveals complex mechanisms of intrinsic resistance to BRAF inhibition. Ann Oncol. 2014;25(5):959‐967. 24504448 10.1093/annonc/mdu049 PMC3999800

[11] Ma Y , Wang L , Neitzel LR , et al. The MAPK pathway regulates intrinsic resistance to BET inhibitors in colorectal cancer. Clin Cancer Res. 2017;23(8):2027‐2037. 27678457 10.1158/1078-0432.CCR-16-0453 PMC5368030

[12] Kalbasi A , Ribas A . Tumour‐intrinsic resistance to immune checkpoint blockade. Nat Rev Immunol. 2020;20(1):25‐39. 31570880 10.1038/s41577-019-0218-4 PMC8499690

[13] Wicki A , Mandala M , Massi D , et al. Acquired resistance to clinical cancer therapy: a twist in physiological signaling. Physiol Rev. 2016;96(3):805‐829. 27142452 10.1152/physrev.00024.2015

[14] Saleh R , Elkord E . Acquired Resistance to Cancer Immunotherapy: Role of Tumor‐Mediated Immunosuppression. Elsevier; 2020:13‐27. 10.1016/j.semcancer.2019.07.017 31362073

[15] Meador CB , Hata AN . Acquired resistance to targeted therapies in NSCLC: updates and evolving insights. Pharmacol Ther. 2020;210:107522. 32151666 10.1016/j.pharmthera.2020.107522 PMC8675642

[16] Hu X , Zhang Z . Understanding the genetic mechanisms of cancer drug resistance using genomic approaches. Trends Genet. 2016;32(2):127‐137. 26689126 10.1016/j.tig.2015.11.003

[17] Kara A , Ozgur A , Nalbantoglu S , Karadag A . DNA repair pathways and their roles in drug resistance for lung adenocarcinoma. Mol Biol Rep. 2021;48(4):3813‐3825. 33856604 10.1007/s11033-021-06314-z

[18] Damia G , Broggini M . Platinum resistance in ovarian cancer: role of DNA repair. Cancers (Basel). 2019;11(1):119. 30669514 10.3390/cancers11010119 PMC6357127

[19] Marine J‐C , Dawson S‐J , Dawson MA . Non‐genetic mechanisms of therapeutic resistance in cancer. Nat Rev Cancer. 2020;20(12):743‐756. 33033407 10.1038/s41568-020-00302-4

[20] Pathania S , Bhatia R , Baldi A , Singh R , Rawal RK . Drug metabolizing enzymes and their inhibitors’ role in cancer resistance. Biomed Pharmacother. 2018;105:53‐65. 29843045 10.1016/j.biopha.2018.05.117

[21] Allain EP , Rouleau M , Levesque E , Guillemette C . Emerging roles for UDP‐glucuronosyltransferases in drug resistance and cancer progression. Br J Cancer. 2020;122(9):1277‐1287. 32047295 10.1038/s41416-019-0722-0 PMC7188667

[22] Buss I , Hamacher A , Sarin N , Kassack MU , Kalayda GV . Relevance of copper transporter 1 and organic cation transporters 1–3 for oxaliplatin uptake and drug resistance in colorectal cancer cells. Metallomics. 2018;10(3):414‐425. 29417972 10.1039/c7mt00334j

[23] Giddings EL , Champagne DP , Wu M‐H , et al. Mitochondrial ATP fuels ABC transporter‐mediated drug efflux in cancer chemoresistance. Nat Commun. 2021;12(1):2804. 33990571 10.1038/s41467-021-23071-6 PMC8121950

[24] Wang J‐Q , Yang Y , Cai C‐Y , et al. Multidrug resistance proteins (MRPs): structure, function and the overcoming of cancer multidrug resistance. Drug Resist Updat. 2021;54:100743. 33513557 10.1016/j.drup.2021.100743

[25] Zhitomirsky B , Assaraf YG . Lysosomes as mediators of drug resistance in cancer. Drug Resist Updat. 2016;24:23‐33. 26830313 10.1016/j.drup.2015.11.004

[26] Wu P , Gao W , Su M , et al. Adaptive mechanisms of tumor therapy resistance driven by tumor microenvironment. Front Cell Dev Biol. 2021;9:641469. 33732706 10.3389/fcell.2021.641469 PMC7957022

[27] Verma H , Singh Bahia M , Choudhary S , Kumar Singh P , Silakari O . Drug metabolizing enzymes‐associated chemo resistance and strategies to overcome it. Drug Metab Rev. 2019;51(2):196‐223. 31203662 10.1080/03602532.2019.1632886

[28] Rahman M , Hasan MR . Cancer metabolism and drug resistance. Metabolites. 2015;5(4):571‐600. 26437434 10.3390/metabo5040571 PMC4693186

[29] Xu Y , Villalona‐Calero MA . Irinotecan: mechanisms of tumor resistance and novel strategies for modulating its activity. Ann Oncol. 2002;13(12):1841‐1851. 12453851 10.1093/annonc/mdf337

[30] Miura K , Kinouchi M , Ishida K , et al. 5‐FU metabolism in cancer and orally‐administrable 5‐FU drugs. Cancers (Basel). 2010;2(3):1717‐1730. 24281184 10.3390/cancers2031717 PMC3837334

[31] Galmarini CM , Mackey JR , Dumontet C . Nucleoside analogues and nucleobases in cancer treatment. Lancet Oncol. 2002;3(7):415‐424. 12142171 10.1016/s1470-2045(02)00788-x

[32] Medina‐Sanson A , Ramirez‐Pacheco A , Moreno‐Guerrero SS , Dorantes‐Acosta EM , Sanchez‐Preza M , Reyes‐Lopez A . Role of genetic polymorphisms of deoxycytidine kinase and cytidine deaminase to predict risk of death in children with acute myeloid leukemia. Biomed Res Int. 2015;2015:309491. 26090398 10.1155/2015/309491 PMC4450239

[33] Wu B , Mao ZJ , Wang Z , et al. Deoxycytidine kinase (DCK) mutations in human acute myeloid leukemia resistant to cytarabine. Acta Haematol. 2021;144(5):534‐541. 33626530 10.1159/000513696

[34] Song JH , Kim SH , Kweon SH , et al. Defective expression of deoxycytidine kinase in cytarabine‐resistant acute myeloid leukemia cells. Int J Oncol. 2009;34(4):1165‐1171. 19287976 10.3892/ijo_00000245

[35] Klanova M , Lorkova L , Vit O , et al. Downregulation of deoxycytidine kinase in cytarabine‐resistant mantle cell lymphoma cells confers cross‐resistance to nucleoside analogs gemcitabine, fludarabine and cladribine, but not to other classes of anti‐lymphoma agents. Mol Cancer. 2014;13:159. 24972933 10.1186/1476-4598-13-159 PMC4094598

[36] Cahard D , McGuigan C , Balzarini J . Aryloxy phosphoramidate triesters as pro‐tides. Mini Rev Med Chem. 2004;4(4):371‐381. 15134540 10.2174/1389557043403936

[37] Slusarczyk M , Lopez MH , Balzarini J , et al. Application of ProTide technology to gemcitabine: a successful approach to overcome the key cancer resistance mechanisms leads to a new agent (NUC‐1031) in clinical development. J Med Chem. 2014;57(4):1531‐1542. 24471998 10.1021/jm401853a

[38] Knox JJ , McNamara MG , Goyal L , et al. Phase III study of NUC‐1031 + cisplatin vs gemcitabine + cisplatin for first‐line treatment of patients with advanced biliary tract cancer (NuTide:121). J Clin Oncol. 2021;39(3 suppl):TPS351‐TPS351.

[39] Spasokoukotskaja T , Sasvari‐Szekely M , Keszler G , Albertioni F , Eriksson S , Staub M . Treatment of normal and malignant cells with nucleoside analogues and etoposide enhances deoxycytidine kinase activity. Eur J Cancer. 1999;35(13):1862‐1867. 10674004 10.1016/s0959-8049(99)00223-3

[40] Huang M , Inukai T , Miyake K , et al. Clofarabine exerts antileukemic activity against cytarabine‐resistant B‐cell precursor acute lymphoblastic leukemia with low deoxycytidine kinase expression. Cancer Med. 2018;7(4):1297‐1316. 29473342 10.1002/cam4.1323 PMC5911575

[41] Riddick DS , Lee C , Ramji S , et al. Cancer chemotherapy and drug metabolism. Drug Metab Dispos. 2005;33(8):1083‐1096. 16049130 10.1124/dmd.105.004374

[42] Kaur G , Gupta SK , Singh P , Ali V , Kumar V , Verma M . Drug‐metabolizing enzymes: role in drug resistance in cancer. Clin Transl Oncol. 2020;22(10):1667‐1680. 32170639 10.1007/s12094-020-02325-7

[43] Esteves F , Rueff J , Kranendonk M . The central role of cytochrome P450 in xenobiotic metabolism—a brief review on a fascinating enzyme family. J Xenobiot. 2021;11(3):94‐114. 34206277 10.3390/jox11030007 PMC8293344

[44] Hofman J , Vagiannis D , Chen S , Guo L . Roles of CYP3A4, CYP3A5 and CYP2C8 drug‐metabolizing enzymes in cellular cytostatic resistance. Chem Biol Interact. 2021;340:109448. 33775687 10.1016/j.cbi.2021.109448 PMC12376141

[45] Garcia‐Martin E , Pizarro RM , Martinez C , et al. Acquired resistance to the anticancer drug paclitaxel is associated with induction of cytochrome P450 2C8. Pharmacogenomics. 2006;7(4):575‐585. 16753005 10.2217/14622416.7.4.575

[46] Morvan VL , Richard E , Cadars M , et al. Cytochrome P450 1B1 polymorphism drives cancer cell stemness and patient outcome in head‐and‐neck carcinoma. Br J Cancer. 2020;123(5):772‐784. 32565541 10.1038/s41416-020-0932-5 PMC7462978

[47] Yada E , Kasajima R , Niida A , et al. Possible role of cytochrome P450 1B1 in the mechanism of gemcitabine resistance in pancreatic cancer. Biomedicines. 2021;9(10):1396. 34680513 10.3390/biomedicines9101396 PMC8533121

[48] McFadyen MCE , Murray GI . Cytochrome P450 1B1: a novel anticancer therapeutic target. Future Oncol. 2005;1(2):259‐263. 16555997 10.1517/14796694.1.2.259

[49] Pastina I , Giovannetti E , Chioni A , et al. Cytochrome 450 1B1 (CYP1B1) polymorphisms associated with response to docetaxel in castration‐resistant prostate cancer (CRPC) patients. BMC Cancer. 2010;10(1):511. 20875115 10.1186/1471-2407-10-511 PMC2955042

[50] Norouzi‐Barough L , Sarookhani MR , Sharifi M , Moghbelinejad S , Jangjoo S , Salehi R . Molecular mechanisms of drug resistance in ovarian cancer. J Cell Physiol. 2018;233(6):4546‐4562. 29152737 10.1002/jcp.26289

[51] McFadyen MC , McLeod HL , Jackson FC , Melvin WT , Doehmer J , Murray GI . Cytochrome P450 CYP1B1 protein expression: a novel mechanism of anticancer drug resistance. Biochem Pharmacol. 2001;62(2):207‐212. 11389879 10.1016/s0006-2952(01)00643-8

[52] Rochat B , Morsman JM , Murray GI , Figg WD , McLeod HL . Human CYP1B1 and anticancer agent metabolism: mechanism for tumor‐specific drug inactivation? J Pharmacol Exp Ther. 2001;296(2):537‐541. 11160641

[53] Martinez VG , O'Connor R , Liang Y , Clynes M . CYP1B1 expression is induced by docetaxel: effect on cell viability and drug resistance. Br J Cancer. 2008;98(3):564‐570. 18212750 10.1038/sj.bjc.6604195 PMC2243158

[54] Carrera AN , Grant MKO , Zordoky BN . CYP1B1 as a therapeutic target in cardio‐oncology. Clin Sci (Lond). 2020;134(21):2897‐2927. 33185690 10.1042/CS20200310 PMC7672255

[55] Dutour R , Poirier D . Inhibitors of cytochrome P450 (CYP) 1B1. Eur J Med Chem. 2017;135:296‐306. 28458135 10.1016/j.ejmech.2017.04.042

[56] Zhu Z , Mu Y , Qi C , et al. CYP1B1 enhances the resistance of epithelial ovarian cancer cells to paclitaxel in vivo and in vitro. Int J Mol Med. 2015;35(2):340‐348. 25516145 10.3892/ijmm.2014.2041 PMC4292762

[57] Mikstacka R , Dutkiewicz Z . New perspectives of CYP1B1 inhibitors in the light of molecular studies. Processes. 2021;9(5):817.

[58] Lin H , Hu B , He X , et al. Overcoming taxol‐resistance in A549 cells: a comprehensive strategy of targeting P‐gp transporter, AKT/ERK pathways, and cytochrome P450 enzyme CYP1B1 by 4‐hydroxyemodin. Biochem Pharmacol. 2020;171:113733. 31783010 10.1016/j.bcp.2019.113733

[59] Cui J , Meng Q , Zhang X , Cui Q , Zhou W , Li S . Design and synthesis of new alpha‐naphthoflavones as cytochrome P450 (CYP) 1B1 inhibitors to overcome docetaxel‐resistance associated with CYP1B1 overexpression. J Med Chem. 2015;58(8):3534‐3547. 25799264 10.1021/acs.jmedchem.5b00265

[60] Chatterjee A , Gupta S . The multifaceted role of glutathione S‐transferases in cancer. Cancer Lett. 2018;433:33‐42. 29959055 10.1016/j.canlet.2018.06.028

[61] Townsend DM , Tew KD . The role of glutathione‐S‐transferase in anti‐cancer drug resistance. Oncogene. 2003;22(47):7369‐7375. 14576844 10.1038/sj.onc.1206940 PMC6361125

[62] Smitherman PK , Townsend AJ , Kute TE , Morrow CS . Role of multidrug resistance protein 2 (MRP2, ABCC2) in alkylating agent detoxification: MRP2 potentiates glutathione S‐transferase A1‐1‐mediated resistance to chlorambucil cytotoxicity. J Pharmacol Exp Ther. 2004;308(1):260‐267. 14569069 10.1124/jpet.103.057729

[63] Cole SPC , Deeley RG . Transport of glutathione and glutathione conjugates by MRP1. Trends Pharmacol Sci. 2006;27(8):438‐446. 16820223 10.1016/j.tips.2006.06.008

[64] Brechbuhl HM , Gould N , Kachadourian R , Riekhof WR , Voelker DR , Day BJ . Glutathione transport is a unique function of the ATP‐binding cassette protein ABCG2. J Biol Chem. 2010;285(22):16582‐16587. 20332504 10.1074/jbc.M109.090506 PMC2878084

[65] Zou M , Hu X , Xu B , et al. Glutathione S‑transferase isozyme alpha 1 is predominantly involved in the cisplatin resistance of common types of solid cancer. Oncol Rep. 2019;41(2):989‐998. 30431119 10.3892/or.2018.6861

[66] Sawers L , Ferguson MJ , Ihrig BR , et al. Glutathione S‐transferase P1 (GSTP1) directly influences platinum drug chemosensitivity in ovarian tumour cell lines. Br J Cancer. 2014;111(6):1150‐1158. 25010864 10.1038/bjc.2014.386 PMC4453841

[67] Mathieu A , Remmelink M , D'Haene N , et al. Development of a chemoresistant orthotopic human nonsmall cell lung carcinoma model in nude mice: analyses of tumor heterogeneity in relation to the immunohistochemical levels of expression of cyclooxygenase‐2, ornithine decarboxylase, lung‐related resistance protein, prostaglandin E synthetase, and glutathione‐S‐transferase‐alpha (GST)‐alpha, GST‐mu, and GST‐pi. Cancer. 2004;101(8):1908‐1918. 15386340 10.1002/cncr.20571

[68] Li Q‐F , Yao R‐Y , Liu K‐w , Lv H‐Y , Jiang T , Liang J . Genetic polymorphism of GSTP1: prediction of clinical outcome to oxaliplatin/5‐FU‐based chemotherapy in advanced gastric cancer. J Korean Med Sci. 2010;25(6):846‐852. 20514304 10.3346/jkms.2010.25.6.846 PMC2877230

[69] Huang Z‐H , Hua D , Du X . Polymorphisms in p53, GSTP1 and XRCC1 predict relapse and survival of gastric cancer patients treated with oxaliplatin‐based adjuvant chemotherapy. Cancer Chemother Pharmacol. 2009;64(5):1001‐1007. 19247656 10.1007/s00280-009-0956-2

[70] Wang Z , Liang S , Lian X , et al. Identification of proteins responsible for adriamycin resistance in breast cancer cells using proteomics analysis. Sci Rep. 2015;5(1):9301. 25818003 10.1038/srep09301 PMC4377623

[71] Pljesa‐Ercegovac M , Savic‐Radojevic A , Matic M , et al. Glutathione transferases: potential targets to overcome chemoresistance in solid tumors. Int J Mol Sci. 2018;19(12):3785. 30487385 10.3390/ijms19123785 PMC6321424

[72] Saleh EM , El‐Awady RA , Abdel Alim MA , Abdel Wahab AHA . Altered expression of proliferation‐inducing and proliferation‐inhibiting genes might contribute to acquired doxorubicin resistance in breast cancer cells. Cell Biochem Biophys. 2009;55(2):95‐105. 19593673 10.1007/s12013-009-9058-3

[73] Tew KD , Townsend DM . Regulatory functions of glutathione S‐transferase P1‐1 unrelated to detoxification. Drug Metab Rev. 2011;43(2):179‐193. 21351850 10.3109/03602532.2011.552912 PMC4603649

[74] Attaoua C , Vincent L‐A , Abdel Jaoued A , et al. Differential involvement of glutathione S‐transferase mu 1 and multidrug resistance protein 1 in melanoma acquired resistance to vinca alkaloids. Fundam Clin Pharmacol. 2015;29(1):62‐71. 25283245 10.1111/fcp.12093

[75] Hansson J , Berhane K , Castro VM , Jungnelius U , Mannervik B , Ringborg U . Sensitization of human melanoma cells to the cytotoxic effect of melphalan by the glutathione transferase inhibitor ethacrynic acid. Cancer Res. 1991;51(1):94‐98. 1988111

[76] Tew KD , Bomber AM , Hoffman SJ . Ethacrynic acid and piriprost as enhancers of cytotoxicity in drug resistant and sensitive cell lines. Cancer Res. 1988;48(13):3622‐3625. 3288331

[77] Federici L , Lo Sterzo C , Pezzola S , et al. Structural basis for the binding of the anticancer compound 6‐(7‐nitro‐2,1,3‐benzoxadiazol‐4‐ylthio)hexanol to human glutathione s‐transferases. Cancer Res. 2009;69(20):8025‐8034. 19808963 10.1158/0008-5472.CAN-09-1314

[78] Pasello M , Michelacci F , Scionti I , et al. Overcoming glutathione S‐transferase P1‐related cisplatin resistance in osteosarcoma. Cancer Res. 2008;68(16):6661‐6668. 18701490 10.1158/0008-5472.CAN-07-5840

[79] Sau A , Filomeni G , Pezzola S , et al. Targeting GSTP1‐1 induces JNK activation and leads to apoptosis in cisplatin‐sensitive and ‐resistant human osteosarcoma cell lines. Mol Biosyst. 2012;8(4):994‐1006. 22068640 10.1039/c1mb05295k

[80] De Luca A , Carpanese D , Rapanotti MC , et al. The nitrobenzoxadiazole derivative MC3181 blocks melanoma invasion and metastasis. Oncotarget. 2017;8(9):15520‐15538. 28107182 10.18632/oncotarget.14690 PMC5362503

[81] Di Paolo V , Fulci C , Rotili D , et al. Characterization of water‐soluble esters of nitrobenzoxadiazole‐based GSTP1‐1 inhibitors for cancer treatment. Biochem Pharmacol. 2020;178:114060. 32473836 10.1016/j.bcp.2020.114060

[82] Dourado DFAR , Fernandes PA , Ramos MJ , Mannervik B . Mechanism of glutathione transferase P1‐1‐catalyzed activation of the prodrug canfosfamide (TLK286, TELCYTA). Biochemistry. 2013;52(45):8069‐8078. 24066958 10.1021/bi4005705

[83] Gelderblom H , Blay JY , Seddon BM , et al. Brostallicin versus doxorubicin as first‐line chemotherapy in patients with advanced or metastatic soft tissue sarcoma: an European organisation for research and treatment of cancer soft tissue and bone sarcoma group randomised phase II and pharmacogenetic study. Eur J Cancer. 2014;50(2):388‐396. 24215845 10.1016/j.ejca.2013.10.002

[84] Raynal C , Pascussi J‐M , Leguelinel G , et al. Pregnane X receptor (PXR) expression in colorectal cancer cells restricts irinotecan chemosensitivity through enhanced SN‐38 glucuronidation. Mol Cancer. 2010;9:46. 20196838 10.1186/1476-4598-9-46 PMC2838814

[85] Landmann H , Proia DA , He S , et al. UDP glucuronosyltransferase 1A expression levels determine the response of colorectal cancer cells to the heat shock protein 90 inhibitor ganetespib. Cell Death Dis. 2014;5(9):e1411. 25210794 10.1038/cddis.2014.378 PMC4540199

[86] López‐Ayllón BD , de Castro‐Carpeño J , Rodriguez C , et al. Biomarkers of erlotinib response in non‐small cell lung cancer tumors that do not harbor the more common epidermal growth factor receptor mutations. Int J Clin Exp Pathol. 2015;8(3):2888. 26045797 PMC4440106

[87] Zahreddine HA , Culjkovic‐Kraljacic B , Assouline S , et al. The sonic hedgehog factor GLI1 imparts drug resistance through inducible glucuronidation. Nature. 2014;511(7507):90‐93. 24870236 10.1038/nature13283 PMC4138053

[88] Ascierto ML , McMiller TL , Berger AE , et al. The intratumoral balance between metabolic and immunologic gene expression is associated with anti‐PD‐1 response in patients with renal cell carcinoma. Cancer Immunol Res. 2016;4(9):726‐733. 27491898 10.1158/2326-6066.CIR-16-0072 PMC5584610

[89] Miners JO , Chau N , Rowland A , et al. Inhibition of human UDP‐glucuronosyltransferase enzymes by lapatinib, pazopanib, regorafenib and sorafenib: implications for hyperbilirubinemia. Biochem Pharmacol. 2017;129:85‐95. 28065859 10.1016/j.bcp.2017.01.002

[90] Osborne MJ , Coutinho de Oliveira L , Volpon L , Zahreddine HA , Borden KLB . Overcoming drug resistance through the development of selective inhibitors of UDP‐glucuronosyltransferase enzymes. J Mol Biol. 2019;431(2):258‐272. 30428301 10.1016/j.jmb.2018.11.007 PMC6331242

[91] Peetla C , Vijayaraghavalu S , Labhasetwar V . Biophysics of cell membrane lipids in cancer drug resistance: implications for drug transport and drug delivery with nanoparticles. Adv Drug Deliv Rev. 2013;65(13‐14):1686‐1698. 24055719 10.1016/j.addr.2013.09.004 PMC3840112

[92] Zalba S , Ten Hagen TLM . Cell membrane modulation as adjuvant in cancer therapy. Cancer Treat Rev. 2017;52:48‐57. 27889637 10.1016/j.ctrv.2016.10.008 PMC5195909

[93] May GL , Wright LC , Dyne M , Mackinnon WB , Fox RM , Mountford CE . Plasma membrane lipid composition of vinblastine sensitive and resistant human leukaemic lymphoblasts. Int J Cancer. 1988;42(5):728‐733. 3263327 10.1002/ijc.2910420517

[94] Peetla C , Bhave R , Vijayaraghavalu S , Stine A , Kooijman E , Labhasetwar V . Drug resistance in breast cancer cells: biophysical characterization of and doxorubicin interactions with membrane lipids. Mol Pharm. 2010;7(6):2334‐2348. 20958074 10.1021/mp100308n PMC2997943

[95] Todor IN , Lukyanova NY , Chekhun VF . The lipid content of cisplatin‐ and doxorubicin‐resistant MCF‐7 human breast cancer cells. Exp Oncol. 2012;34(2):97‐100. 23013760

[96] Rivel T , Ramseyer C , Yesylevskyy S . The asymmetry of plasma membranes and their cholesterol content influence the uptake of cisplatin. Sci Rep. 2019;9(1):5627. 30948733 10.1038/s41598-019-41903-w PMC6449338

[97] van Lummel M , van Blitterswijk WJ , Vink SR , et al. Enriching lipid nanovesicles with short‐chain glucosylceramide improves doxorubicin delivery and efficacy in solid tumors. FASEB J. 2011;25(1):280‐289. 20876209 10.1096/fj.10-163709

[98] Pedrosa LRC , van Hell A , Suss R , et al. Improving intracellular doxorubicin delivery through nanoliposomes equipped with selective tumor cell membrane permeabilizing short‐chain sphingolipids. Pharm Res. 2013;30(7):1883‐1895. 23666266 10.1007/s11095-013-1031-6

[99] Adada M , Luberto C , Canals D . Inhibitors of the sphingomyelin cycle: sphingomyelin synthases and sphingomyelinases. Chem Phys Lipids. 2016;197:45‐59. 26200918 10.1016/j.chemphyslip.2015.07.008

[100] Vijayaraghavalu S , Peetla C , Lu S , Labhasetwar V . Epigenetic modulation of the biophysical properties of drug‐resistant cell lipids to restore drug transport and endocytic functions. Mol Pharm. 2012;9(9):2730‐2742. 22817326 10.1021/mp300281t PMC3433581

[101] Bose RJ , Paulmurugan R , Moon J , Lee S‐H , Park H . Cell membrane‐coated nanocarriers: the emerging targeted delivery system for cancer theranostics. Drug Discov Today. 2018;23(4):891‐899. 29426004 10.1016/j.drudis.2018.02.001

[102] Fang RH , Hu C‐MJ , Luk BT , et al. Cancer cell membrane‐coated nanoparticles for anticancer vaccination and drug delivery. Nano Lett. 2014;14(4):2181‐2188. 24673373 10.1021/nl500618u PMC3985711

[103] Wu P , Yin D , Liu J , et al. Cell membrane based biomimetic nanocomposites for targeted therapy of drug resistant EGFR‐mutated lung cancer. Nanoscale. 2019;11(41):19520‐19528. 31573595 10.1039/c9nr05791a

[104] Gao Y , Zhu Y , Xu X , et al. Surface PEGylated cancer cell membrane‐coated nanoparticles for codelivery of curcumin and doxorubicin for the treatment of multidrug resistant esophageal carcinoma. Front Cell Dev Biol. 2021;9:688070. 34386493 10.3389/fcell.2021.688070 PMC8353447

[105] Lu K , Li Z , Hu Q , Sun J , Chen M . CRPC membrane‐camouflaged, biomimetic nanosystem for overcoming castration‐resistant prostate cancer by cellular vehicle‐aided tumor targeting. Int J Mol Sci. 2022;23(7):3623. 35408983 10.3390/ijms23073623 PMC8998744

[106] Perland E , Fredriksson R . Classification systems of secondary active transporters. Trends Pharmacol Sci. 2017;38(3):305‐315. 27939446 10.1016/j.tips.2016.11.008

[107] Al‐Abdulla R , Perez‐Silva L , Abete L , Romero MR , Briz O , Marin JJG . Unraveling ‘The Cancer Genome Atlas’ information on the role of SLC transporters in anticancer drug uptake. Expert Rev Clin Pharmacol. 2019;12(4):329‐341. 30744443 10.1080/17512433.2019.1581605

[108] Wu Z , Xu J , Liang C , et al. Emerging roles of the solute carrier family in pancreatic cancer. Clin Transl Med. 2021;11(3):e356. 33783998 10.1002/ctm2.356 PMC7989705

[109] Buxhofer‐Ausch V , Secky L , Wlcek K , et al. Tumor‐specific expression of organic anion‐transporting polypeptides: transporters as novel targets for cancer therapy. J Drug Deliv. 2013;2013:863539. 23431456 10.1155/2013/863539 PMC3574750

[110] Sun R , Ying Y , Tang Z , et al. The emerging role of the SLCO1B3 protein in cancer resistance. Protein Pept Lett. 2020;27(1):17‐29. 31556849 10.2174/0929866526666190926154248 PMC6978646

[111] Zimmerman EI , Hu S , Roberts JL , et al. Contribution of OATP1B1 and OATP1B3 to the disposition of sorafenib and sorafenib‐glucuronide. Clin Cancer Res. 2013;19(6):1458‐1466. 23340295 10.1158/1078-0432.CCR-12-3306 PMC3602278

[112] de Morree ES , Bottcher R , van Soest RJ , et al. Loss of SLCO1B3 drives taxane resistance in prostate cancer. Br J Cancer. 2016;115(6):674‐681. 27537383 10.1038/bjc.2016.251 PMC5023781

[113] Sissung TM , Ley AM , Strope JD , et al. Differential expression of OATP1B3 mediates unconjugated testosterone influx. Mol Cancer Res. 2017;15(8):1096‐1105. 28389619 10.1158/1541-7786.MCR-16-0477 PMC5540879

[114] Alsinnawi M , Zhang A , Bianchi‐Frias D , et al. Association of prostate cancer SLCO gene expression with Gleason grade and alterations following androgen deprivation therapy. Prostate Cancer Prostatic Dis. 2019;22(4):560‐568. 30890759 10.1038/s41391-019-0141-6 PMC6752995

[115] Haberkorn B , Oswald S , Kehl N , et al. Cancer‐type organic anion transporting polypeptide 1B3 (Ct‐OATP1B3) is localized in lysosomes and mediates resistance against kinase inhibitors. Mol Pharmacol. 2022;102(6):248‐258. 10.1124/molpharm.122.000539 36167426

[116] Falguieres T . ABC transporters in human diseases: future directions and therapeutic perspectives. Int J Mol Sci. 2022;23(8):4250. 35457067 10.3390/ijms23084250 PMC9028344

[117] Juliano RL , Ling V . A surface glycoprotein modulating drug permeability in Chinese hamster ovary cell mutants. Biochim Biophys Acta. 1976;455(1):152‐162. 990323 10.1016/0005-2736(76)90160-7

[118] Wang J‐Q , Wu Z‐X , Yang Y , et al. ATP‐binding cassette (ABC) transporters in cancer: a review of recent updates. J Evid Based Med. 2021;14(3):232‐256. 34388310 10.1111/jebm.12434

[119] Thomas C , Tampe R . Structural and mechanistic principles of ABC transporters. Annu Rev Biochem. 2020;89:605‐636. 32569521 10.1146/annurev-biochem-011520-105201

[120] Chen XY . Natural product as substrates of ABC transporters: a review. Recent Pat Anti‐Cancer Drug Discov. 2021;16(2):222‐238. 10.2174/1574892816666210218220943 33602076

[121] Anreddy N , Gupta P , Kathawala RJ , Patel A , Wurpel JND , Chen Z‐S . Tyrosine kinase inhibitors as reversal agents for ABC transporter mediated drug resistance. Molecules. 2014;19(9):13848‐13877. 25191874 10.3390/molecules190913848 PMC6271846

[122] Dong X‐D , Zhang M , Cai C‐Y , et al. Overexpression of ABCB1 associated with the resistance to the KRAS‐G12C specific inhibitor ARS‐1620 in cancer cells. Front Pharmacol. 2022;13:843829. 35281897 10.3389/fphar.2022.843829 PMC8905313

[123] Yang Y , Teng Q‐X , Wu Z‐X , et al. PBK/TOPK inhibitor OTS964 resistance is mediated by ABCB1‐dependent transport function in cancer: in vitro and in vivo study. Mol Cancer. 2022;21(1):40. 35135547 10.1186/s12943-022-01512-0 PMC8822834

[124] Fu H , Wu Z‐X , Lei Z‐N , et al. The resistance of cancer cells to palbociclib, a cyclin‐dependent kinase 4/6 inhibitor, is mediated by the ABCB1 transporter. Front Pharmacol. 2022;13:861642. 35350768 10.3389/fphar.2022.861642 PMC8957877

[125] Parrish KE , Pokorny J , Mittapalli RK , Bakken K , Sarkaria JN , Elmquist WF . Efflux transporters at the blood‐brain barrier limit delivery and efficacy of cyclin‐dependent kinase 4/6 inhibitor palbociclib (PD‐0332991) in an orthotopic brain tumor model. J Pharmacol Exp Ther. 2015;355(2):264‐271. 26354993 10.1124/jpet.115.228213 PMC4613960

[126] Mittapalli RK , Chung AH , Parrish KE , et al. ABCG2 and ABCB1 limit the efficacy of dasatinib in a PDGF‐B‐driven brainstem glioma model. Mol Cancer Ther. 2016;15(5):819‐829. 26883271 10.1158/1535-7163.MCT-15-0093 PMC4873451

[127] Engle K , Kumar G . Cancer multidrug‐resistance reversal by ABCB1 inhibition: a recent update. Eur J Med Chem. 2022;239:114542. 35751979 10.1016/j.ejmech.2022.114542

[128] Ji N , Yang Y , Cai C‐Y , et al. Selonsertib (GS‐4997), an ASK1 inhibitor, antagonizes multidrug resistance in ABCB1‐ and ABCG2‐overexpressing cancer cells. Cancer Lett. 2019;440‐441:82‐93. 10.1016/j.canlet.2018.10.007 PMC8132112 30315846

[129] Wu Z‐X , Teng Q‐X , Yang Y , et al. MET inhibitor tepotinib antagonizes multidrug resistance mediated by ABCG2 transporter: in vitro and in vivo study. Acta Pharm Sin B. 2022;12(5):2609‐2618. 35646541 10.1016/j.apsb.2021.12.018 PMC9136566

[130] Zhang Y , Wu Z‐X , Yang Y , et al. Poziotinib inhibits the efflux activity of the ABCB1 and ABCG2 transporters and the expression of the ABCG2 transporter protein in multidrug resistant colon cancer cells. Cancers (Basel). 2020;12(11):3249. 33158067 10.3390/cancers12113249 PMC7694178

[131] Fanelli M , Hattinger CM , Vella S , et al. Targeting ABCB1 and ABCC1 with their specific inhibitor CBT‐1(R) can overcome drug resistance in osteosarcoma. Curr Cancer Drug Targets. 2016;16(3):261‐274. 26548759 10.2174/1568009616666151106120434

[132] Tao L‐Y , Liang Y‐J , Wang F , et al. Cediranib (recentin, AZD2171) reverses ABCB1‐ and ABCC1‐mediated multidrug resistance by inhibition of their transport function. Cancer Chemother Pharmacol. 2009;64(5):961‐969. 19255759 10.1007/s00280-009-0949-1

[133] Stefan SM . Multi‐target ABC transporter modulators: what next and where to go? Future Med Chem. 2019;11(18):2353‐2358. 31516029 10.4155/fmc-2019-0185

[134] Tiash S , Chowdhury EH . siRNAs targeting multidrug transporter genes sensitise breast tumour to doxorubicin in a syngeneic mouse model. J Drug Target. 2019;27(3):325‐337. 30221549 10.1080/1061186X.2018.1525388

[135] Wang Y , Wang Y , Qin Z , et al. The role of non‐coding RNAs in ABC transporters regulation and their clinical implications of multidrug resistance in cancer. Expert Opin Drug Metab Toxicol. 2021;17(3):291‐306. 33544643 10.1080/17425255.2021.1887139

[136] Yi H , Liu L , Sheng N , et al. Synergistic therapy of doxorubicin and miR‐129‐5p with self‐cross‐linked bioreducible polypeptide nanoparticles reverses multidrug resistance in cancer cells. Biomacromolecules. 2016;17(5):1737‐1747. 27029378 10.1021/acs.biomac.6b00141

[137] Yan L , Ding B , Liu H , et al. Inhibition of SMYD2 suppresses tumor progression by down‐regulating microRNA‐125b and attenuates multi‐drug resistance in renal cell carcinoma. Theranostics. 2019;9(26):8377‐8391. 31754403 10.7150/thno.37628 PMC6857066

[138] X‐k Wang , J‐h He , J‐h Xu , et al. Afatinib enhances the efficacy of conventional chemotherapeutic agents by eradicating cancer stem‐like cells. Cancer Res. 2014;74(16):4431‐4445. 24972892 10.1158/0008-5472.CAN-13-3553

[139] Martin V , Sanchez‐Sanchez AM , Herrera F , et al. Melatonin‐induced methylation of the ABCG2/BCRP promoter as a novel mechanism to overcome multidrug resistance in brain tumour stem cells. Br J Cancer. 2013;108(10):2005‐2012. 23632480 10.1038/bjc.2013.188 PMC3670480

[140] Fultang N , Illendula A , Lin J , Pandey MK , Klase Z , Peethambaran B . ROR1 regulates chemoresistance in breast cancer via modulation of drug efflux pump ABCB1. Sci Rep. 2020;10(1):1821. 32020017 10.1038/s41598-020-58864-0 PMC7000766

[141] Liu W‐J , Du Y , Wen R , Yang M , Xu J . Drug resistance to targeted therapeutic strategies in non‐small cell lung cancer. Pharmacol Ther. 2020;206:107438. 31715289 10.1016/j.pharmthera.2019.107438

[142] Sarmento‐Ribeiro AB , Scorilas A , Goncalves AC , Efferth T , Trougakos IP . The emergence of drug resistance to targeted cancer therapies: clinical evidence. Drug Resist Updat. 2019;47:100646. 31733611 10.1016/j.drup.2019.100646

[143] Di Noia V , D'Aveni A , D'Argento E , et al. Treating disease progression with osimertinib in EGFR‐mutated non‐small‐cell lung cancer: novel targeted agents and combination strategies. ESMO Open. 2021;6(6):100280. 34634633 10.1016/j.esmoop.2021.100280 PMC8506968

[144] Leonetti A , Sharma S , Minari R , Perego P , Giovannetti E , Tiseo M . Resistance mechanisms to osimertinib in EGFR‐mutated non‐small cell lung cancer. Br J Cancer. 2019;121(9):725‐737. 31564718 10.1038/s41416-019-0573-8 PMC6889286

[145] Piotrowska Z , Niederst MJ , Karlovich CA , et al. Heterogeneity underlies the emergence of EGFRT790 wild‐type clones following treatment of T790M‐positive cancers with a third‐generation EGFR inhibitor. Cancer Discov. 2015;5(7):713‐722. 25934077 10.1158/2159-8290.CD-15-0399 PMC4497836

[146] Thress KS , Paweletz CP , Felip E , et al. Acquired EGFR C797S mutation mediates resistance to AZD9291 in non‐small cell lung cancer harboring EGFR T790M. Nat Med. 2015;21(6):560‐562. 25939061 10.1038/nm.3854 PMC4771182

[147] Jia Y , Yun C‐H , Park E , et al. Overcoming EGFR(T790M) and EGFR(C797S) resistance with mutant‐selective allosteric inhibitors. Nature. 2016;534(7605):129‐132. 27251290 10.1038/nature17960 PMC4929832

[148] Lu X , Zhang T , Zhu S‐J , et al. Discovery of JND3229 as a new EGFR(C797S) mutant inhibitor with in vivo monodrug efficacy. ACS Med Chem Lett. 2018;9(11):1123‐1127. 30429956 10.1021/acsmedchemlett.8b00373 PMC6231186

[149] To C , Jang J , Chen T , et al. Single and dual targeting of mutant EGFR with an allosteric inhibitor. Cancer Discov. 2019;9(7):926‐943. 31092401 10.1158/2159-8290.CD-18-0903 PMC6664433

[150] Drilon A , Laetsch TW , Kummar S , et al. Efficacy of larotrectinib in TRK fusion‐positive cancers in adults and children. N Engl J Med. 2018;378(8):731‐739. 29466156 10.1056/NEJMoa1714448 PMC5857389

[151] Drilon A , Li G , Dogan S , et al. What hides behind the MASC: clinical response and acquired resistance to entrectinib after ETV6‐NTRK3 identification in a mammary analogue secretory carcinoma (MASC). Ann Oncol. 2016;27(5):920‐926. 26884591 10.1093/annonc/mdw042 PMC4843186

[152] Kwak EL , Bang Y‐J , Camidge DR , et al. Anaplastic lymphoma kinase inhibition in non‐small‐cell lung cancer. N Engl J Med. 2010;363(18):1693‐1703. 20979469 10.1056/NEJMoa1006448 PMC3014291

[153] Katayama R , Shaw AT , Khan TM , et al. Mechanisms of acquired crizotinib resistance in ALK‐rearranged lung cancers. Sci Transl Med. 2012;4(120):120ra17. 10.1126/scitranslmed.3003316 PMC3385512 22277784

[154] Gainor JF , Varghese AM , Ou S‐HI , et al. ALK rearrangements are mutually exclusive with mutations in EGFR or KRAS: an analysis of 1,683 patients with non‐small cell lung cancer. Clin Cancer Res. 2013;19(15):4273‐4281. 23729361 10.1158/1078-0432.CCR-13-0318 PMC3874127

[155] Doebele RC , Pilling AB , Aisner DL , et al. Mechanisms of resistance to crizotinib in patients with ALK gene rearranged non‐small cell lung cancer. Clin Cancer Res. 2012;18(5):1472‐1482. 22235099 10.1158/1078-0432.CCR-11-2906 PMC3311875

[156] Sasaki T , Okuda K , Zheng W , et al. The neuroblastoma‐associated F1174L ALK mutation causes resistance to an ALK kinase inhibitor in ALK‐translocated cancers. Cancer Res. 2010;70(24):10038‐10043. 21030459 10.1158/0008-5472.CAN-10-2956 PMC3045808

[157] Sasaki T , Koivunen J , Ogino A , et al. A novel ALK secondary mutation and EGFR signaling cause resistance to ALK kinase inhibitors. Cancer Res. 2011;71(18):6051‐6060. 21791641 10.1158/0008-5472.CAN-11-1340 PMC3278914

[158] Katayama R , Khan TM , Benes C , et al. Therapeutic strategies to overcome crizotinib resistance in non‐small cell lung cancers harboring the fusion oncogene EML4‐ALK. Proc Natl Acad Sci USA. 2011;108(18):7535‐7540. 21502504 10.1073/pnas.1019559108 PMC3088626

[159] Marsilje TH , Pei W , Chen B , et al. Synthesis, structure‐activity relationships, and in vivo efficacy of the novel potent and selective anaplastic lymphoma kinase (ALK) inhibitor 5‐chloro‐N2‐(2‐isopropoxy‐5‐methyl‐4‐(piperidin‐4‐yl)phenyl)‐N4‐(2‐(isopropylsulfonyl)phenyl)pyrimidine‐2,4‐diamine (LDK378) currently in phase 1 and phase 2 clinical trials. J Med Chem. 2013;56(14):5675‐5690. 23742252 10.1021/jm400402q

[160] Friboulet L , Li N , Katayama R , et al. The ALK inhibitor ceritinib overcomes crizotinib resistance in non‐small cell lung cancer. Cancer Discov. 2014;4(6):662‐673. 24675041 10.1158/2159-8290.CD-13-0846 PMC4068971

[161] Shaw AT , Kim D‐W , Mehra R , et al. Ceritinib in ALK‐rearranged non‐small‐cell lung cancer. N Engl J Med. 2014;370(13):1189‐1197. 24670165 10.1056/NEJMoa1311107 PMC4079055

[162] Ostrem JML , Shokat KM . Direct small‐molecule inhibitors of KRAS: from structural insights to mechanism‐based design. Nat Rev Drug Discov. 2016;15(11):771‐785. 27469033 10.1038/nrd.2016.139

[163] Papke B , Der CJ . Drugging RAS: know the enemy. Science. 2017;355(6330):1158‐1163. 28302824 10.1126/science.aam7622

[164] Karoulia Z , Gavathiotis E , Poulikakos PI . New perspectives for targeting RAF kinase in human cancer. Nat Rev Cancer. 2017;17(11):676‐691. 28984291 10.1038/nrc.2017.79 PMC6000833

[165] Lito P , Rosen N , Solit DB . Tumor adaptation and resistance to RAF inhibitors. Nat Med. 2013;19(11):1401‐1409. 24202393 10.1038/nm.3392

[166] Caunt CJ , Sale MJ , Smith PD , Cook SJ . MEK1 and MEK2 inhibitors and cancer therapy: the long and winding road. Nat Rev Cancer. 2015;15(10):577‐592. 26399658 10.1038/nrc4000

[167] Ryan MB , Fece de la Cruz F , Phat S , et al. Vertical pathway inhibition overcomes adaptive feedback resistance to KRAS(G12C) inhibition. Clin Cancer Res. 2020;26(7):1633‐1643. 31776128 10.1158/1078-0432.CCR-19-3523 PMC7124991

[168] Xue JY , Zhao Y , Aronowitz J , et al. Rapid non‐uniform adaptation to conformation‐specific KRAS(G12C) inhibition. Nature. 2020;577(7790):421‐425. 31915379 10.1038/s41586-019-1884-x PMC7308074

[169] Poulikakos PI , Zhang C , Bollag G , Shokat KM , Rosen N . RAF inhibitors transactivate RAF dimers and ERK signalling in cells with wild‐type BRAF. Nature. 2010;464(7287):427‐430. 20179705 10.1038/nature08902 PMC3178447

[170] Lito P , Saborowski A , Yue J , et al. Disruption of CRAF‐mediated MEK activation is required for effective MEK inhibition in KRAS mutant tumors. Cancer Cell. 2014;25(5):697‐710. 24746704 10.1016/j.ccr.2014.03.011 PMC4049532

[171] Little AS , Balmanno K , Sale MJ , et al. Amplification of the driving oncogene, KRAS or BRAF, underpins acquired resistance to MEK1/2 inhibitors in colorectal cancer cells. Sci Signal. 2011;4(166):ra17. 21447798 10.1126/scisignal.2001752

[172] Sale MJ , Balmanno K , Saxena J , et al. MEK1/2 inhibitor withdrawal reverses acquired resistance driven by BRAF(V600E) amplification whereas KRAS(G13D) amplification promotes EMT‐chemoresistance. Nat Commun. 2019;10(1):2030. 31048689 10.1038/s41467-019-09438-w PMC6497655

[173] Juric D , Castel P , Griffith M , et al. Convergent loss of PTEN leads to clinical resistance to a PI(3)Kalpha inhibitor. Nature. 2015;518(7538):240‐244. 25409150 10.1038/nature13948 PMC4326538

[174] Patch A‐M , Christie EL , Etemadmoghadam D , et al. Corrigendum: whole‐genome characterization of chemoresistant ovarian cancer. Nature. 2015;527(7578):398. 10.1038/nature15716 26503049

[175] Lee CK , Wu Y‐L , Ding PN , et al. Impact of specific epidermal growth factor receptor (EGFR) mutations and clinical characteristics on outcomes after treatment with EGFR tyrosine kinase inhibitors versus chemotherapy in EGFR‐mutant lung cancer: a meta‐analysis. J Clin Oncol. 2015;33(17):1958‐1965. 25897154 10.1200/JCO.2014.58.1736

[176] Kancha RK , von Bubnoff N , Peschel C , Duyster J . Functional analysis of epidermal growth factor receptor (EGFR) mutations and potential implications for EGFR targeted therapy. Clin Cancer Res. 2009;15(2):460‐467. 19147750 10.1158/1078-0432.CCR-08-1757

[177] Yarden Y , Sliwkowski MX . Untangling the ErbB signalling network. Nat Rev Mol Cell Biol. 2001;2(2):127‐137. 11252954 10.1038/35052073

[178] Ohashi K , Sequist LV , Arcila ME , et al. Lung cancers with acquired resistance to EGFR inhibitors occasionally harbor BRAF gene mutations but lack mutations in KRAS, NRAS, or MEK1. Proc Natl Acad Sci USA. 2012;109(31):E2127‐E2133. 22773810 10.1073/pnas.1203530109 PMC3411967

[179] Karachaliou N , Codony‐Servat J , Teixido C , et al. BIM and mTOR expression levels predict outcome to erlotinib in EGFR‐mutant non‐small‐cell lung cancer. Sci Rep. 2015;5(1):17499. 26639561 10.1038/srep17499 PMC4671004

[180] Kawabata S , Mercado‐Matos JR , Hollander MC , et al. Rapamycin prevents the development and progression of mutant epidermal growth factor receptor lung tumors with the acquired resistance mutation T790M. Cell Rep. 2014;7(6):1824‐1832. 24931608 10.1016/j.celrep.2014.05.039 PMC4110638

[181] Engelman JA , Zejnullahu K , Mitsudomi T , et al. MET amplification leads to gefitinib resistance in lung cancer by activating ERBB3 signaling. Science. 2007;316(5827):1039‐1043. 17463250 10.1126/science.1141478

[182] Shaw AT , Ou S‐HI , Bang Y‐J , et al. Crizotinib in ROS1‐rearranged non‐small‐cell lung cancer. N Engl J Med. 2014;371(21):1963‐1971. 25264305 10.1056/NEJMoa1406766 PMC4264527

[183] Drilon A , Ou S‐HI , Cho BC , et al. Repotrectinib (TPX‐0005) is a next‐generation ROS1/TRK/ALK inhibitor that potently inhibits ROS1/TRK/ALK solvent‐ front mutations. Cancer Discov. 2018;8(10):1227‐1236. 30093503 10.1158/2159-8290.CD-18-0484

[184] Katayama R , Gong B , Togashi N , et al. The new‐generation selective ROS1/NTRK inhibitor DS‐6051b overcomes crizotinib resistant ROS1‐G2032R mutation in preclinical models. Nat Commun. 2019;10(1):3604. 31399568 10.1038/s41467-019-11496-z PMC6688997

[185] Drilon A , Siena S , Ou S‐HI , et al. Safety and antitumor activity of the multitargeted pan‐TRK, ROS1, and ALK inhibitor entrectinib: combined results from two phase I trials (ALKA‐372‐001 and STARTRK‐1). Cancer Discov. 2017;7(4):400‐409. 28183697 10.1158/2159-8290.CD-16-1237 PMC5380583

[186] Watanabe J , Furuya N , Fujiwara Y . Appearance of a BRAF mutation conferring resistance to crizotinib in non‐small cell lung cancer harboring oncogenic ROS1 fusion. J Thorac Oncol. 2018;13(4):e66‐e69. 29576302 10.1016/j.jtho.2017.11.125

[187] Zhu Y‐C , Lin X‐P , Li X‐F , et al. Concurrent ROS1 gene rearrangement and KRAS mutation in lung adenocarcinoma: a case report and literature review. Thorac Cancer. 2018;9(1):159‐163. 28971587 10.1111/1759-7714.12518 PMC5754306

[188] McCoach CE , Le AT , Gowan K , et al. Resistance mechanisms to targeted therapies in ROS1(+) and ALK(+) non‐small cell lung cancer. Clin Cancer Res. 2018;24(14):3334‐3347. 29636358 10.1158/1078-0432.CCR-17-2452 PMC6050099

[189] He Y , Sheng W , Hu W , et al. Different types of ROS1 fusion partners yield comparable efficacy to crizotinib. Oncol Res. 2019;27(8):901‐910. 30940295 10.3727/096504019X15509372008132 PMC7848361

[190] Zhu VW , Klempner SJ , Ou S‐HI . Receptor tyrosine kinase fusions as an actionable resistance mechanism to EGFR TKIs in EGFR‐mutant non‐small‐cell lung cancer. Trends Cancer. 2019;5(11):677‐692. 31735287 10.1016/j.trecan.2019.09.008

[191] Dagogo‐Jack I , Rooney M , Nagy RJ , et al. Molecular analysis of plasma from patients with ROS1‐positive NSCLC. J Thorac Oncol. 2019;14(5):816‐824. 30664990 10.1016/j.jtho.2019.01.009 PMC6486857

[192] Sato H , Schoenfeld AJ , Siau E , et al. MAPK pathway alterations correlate with poor survival and drive resistance to therapy in patients with lung cancers driven by ROS1 fusions. Clin Cancer Res. 2020;26(12):2932‐2945. 32122926 10.1158/1078-0432.CCR-19-3321 PMC8034819

[193] Cocco E , Scaltriti M , Drilon A . NTRK fusion‐positive cancers and TRK inhibitor therapy. Nat Rev Clin Oncol. 2018;15(12):731‐747. 30333516 10.1038/s41571-018-0113-0 PMC6419506

[194] Gainor JF , Tseng D , Yoda S , et al. Patterns of metastatic spread and mechanisms of resistance to crizotinib in ROS1‐positive non‐small‐cell lung cancer. JCO Precis Oncol. 2017;2017:1‐13. 10.1200/PO.17.00063 PMC5766287 29333528

[195] Sehgal K , Piper‐Vallillo AJ , Viray H , Khan AM , Rangachari D , Costa DB . Cases of ROS1‐rearranged lung cancer: when to use crizotinib, entrectinib, lorlatinib, and beyond? Precis Cancer Med. 2020;3:17‐17. 32776005 10.21037/pcm-2020-potb-02 PMC7410006

[196] Li L‐Y , Guan Y‐d , Chen X‐S , Yang J‐M , Cheng Y . DNA repair pathways in cancer therapy and resistance. Front Pharmacol. 2020;11:629266. 33628188 10.3389/fphar.2020.629266 PMC7898236

[197] Sethy C , Kundu CN . 5‐Fluorouracil (5‐FU) resistance and the new strategy to enhance the sensitivity against cancer: implication of DNA repair inhibition. Biomed Pharmacother. 2021;137:111285. 33485118 10.1016/j.biopha.2021.111285

[198] Liu J‐L , Huang W‐S , Lee K‐C , Tung S‐Y , Chen C‐N , Chang S‐F . Effect of 5‐fluorouracil on excision repair cross‐complementing 1 expression and consequent cytotoxicity regulation in human gastric cancer cells. J Cell Biochem. 2018;119(10):8472‐8480. 30011079 10.1002/jcb.27073

[199] Oliver JA , Ortiz R , Jimenez‐Luna C , et al. MMR‐proficient and MMR‐deficient colorectal cancer cells: 5‐fluorouracil treatment response and correlation to CD133 and MGMT expression. J Biosci. 2020;45(1):121. 33097678

[200] Honecker F , Wermann H , Mayer F , et al. Microsatellite instability, mismatch repair deficiency, and BRAF mutation in treatment‐resistant germ cell tumors. J Clin Oncol. 2009;27(13):2129‐2136. 19289622 10.1200/JCO.2008.18.8623

[201] Song M , Cui M , Liu K . Therapeutic strategies to overcome cisplatin resistance in ovarian cancer. Eur J Med Chem. 2022;232:114205. 35217497 10.1016/j.ejmech.2022.114205

[202] Bouwman P , Jonkers J . Molecular pathways: how can BRCA‐mutated tumors become resistant to PARP inhibitors? Clin Cancer Res. 2014;20(3):540‐547. 24270682 10.1158/1078-0432.CCR-13-0225

[203] Xiao Y , Lin F‐T , Lin W‐C . ACTL6A promotes repair of cisplatin‐induced DNA damage, a new mechanism of platinum resistance in cancer. Proc Natl Acad Sci USA. 2021;118(3):e2015808118. 33408251 10.1073/pnas.2015808118 PMC7826354

[204] Weller M , Tabatabai G , Kastner B , et al. MGMT promoter methylation is a strong prognostic biomarker for benefit from dose‐intensified temozolomide rechallenge in progressive glioblastoma: the DIRECTOR trial. Clin Cancer Res. 2015;21(9):2057‐2064. 25655102 10.1158/1078-0432.CCR-14-2737

[205] Abe H , Natsumeda M , Okada M , et al. MGMT expression contributes to temozolomide resistance in H3K27M‐mutant diffuse midline gliomas. Front Oncol. 2019;9:1568. 32039031 10.3389/fonc.2019.01568 PMC6985080

[206] Brandner S , McAleenan A , Kelly C , et al. MGMT promoter methylation testing to predict overall survival in people with glioblastoma treated with temozolomide: a comprehensive meta‐analysis based on a cochrane systematic review. Neuro Oncol. 2021;23(9):1457‐1469. 34467991 10.1093/neuonc/noab105 PMC8408882

[207] Jacot W , Lopez‐Crapez E , Mollevi C , et al. BRCA1 promoter hypermethylation is associated with good prognosis and chemosensitivity in triple‐negative breast cancer. Cancers (Basel). 2020;12(4):828. 32235500 10.3390/cancers12040828 PMC7225997

[208] Mazloumi Z , Farahzadi R , Rafat A , et al. Effect of aberrant DNA methylation on cancer stem cell properties. Exp Mol Pathol. 2022;125:104757. 35339454 10.1016/j.yexmp.2022.104757

[209] Watson ZL , Yamamoto TM , McMellen A , et al. Histone methyltransferases EHMT1 and EHMT2 (GLP/G9A) maintain PARP inhibitor resistance in high‐grade serous ovarian carcinoma. Clin Epigenetics. 2019;11(1):165. 31775874 10.1186/s13148-019-0758-2 PMC6882350

[210] Singh R , Fazal Z , Corbet AK , et al. Epigenetic remodeling through downregulation of polycomb repressive complex 2 mediates chemotherapy resistance in testicular germ cell tumors. Cancers (Basel). 2019;11(6):796. 31181810 10.3390/cancers11060796 PMC6627640

[211] Patel N , Garikapati KR , Pandita RK , et al. Erratum: miR‐15a/miR‐16 down‐regulates BMI1, impacting Ub‐H2A mediated DNA repair and breast cancer cell sensitivity to doxorubicin. Sci Rep. 2017;7(1):12932. 29018209 10.1038/s41598-017-12314-6 PMC5635132

[212] Qian L , Fei Q , Zhang H , et al. lncRNA HOTAIR promotes DNA repair and radioresistance of breast cancer via EZH2. DNA Cell Biol. 2020;39(12):2166‐2173. 10.1089/dna.2020.5771 33136445

[213] Edinger AL , Thompson CB . Death by design: apoptosis, necrosis and autophagy. Curr Opin Cell Biol. 2004;16(6):663‐669. 15530778 10.1016/j.ceb.2004.09.011

[214] Li M , Le W , Zhang X‐M , Zhang Y‐J , Jiang J , Liu P‐Y . The M476W/Q482H mutation of procaspase‐8 restored caspase‐8‐mediated apoptosis. Biochem Biophys Res Commun. 2019;514(3):653‐658. 31078261 10.1016/j.bbrc.2019.05.023

[215] Roos WP , Thomas AD , Kaina B . DNA damage and the balance between survival and death in cancer biology. Nat Rev Cancer. 2016;16(1):20‐33. 26678314 10.1038/nrc.2015.2

[216] Zhang W , Konopleva M , Ruvolo VR , et al. Sorafenib induces apoptosis of AML cells via Bim‐mediated activation of the intrinsic apoptotic pathway. Leukemia. 2008;22(4):808‐818. 18200035 10.1038/sj.leu.2405098

[217] Zuo J , Ishikawa T , Boutros S , Xiao Z , Humtsoe JO , Kramer RH . Bcl‐2 overexpression induces a partial epithelial to mesenchymal transition and promotes squamous carcinoma cell invasion and metastasis. Mol Cancer Res. 2010;8(2):170‐182. 20145039 10.1158/1541-7786.MCR-09-0354

[218] Real PJ , Sierra A , De Juan A , Segovia JC , Lopez‐Vega JM , Fernandez‐Luna JL . Resistance to chemotherapy via Stat3‐dependent overexpression of Bcl‐2 in metastatic breast cancer cells. Oncogene. 2002;21(50):7611‐7618. 12400004 10.1038/sj.onc.1206004

[219] Ritter V , Krautter F , Klein D , Jendrossek V , Rudner J . Bcl‐2/Bcl‐xL inhibitor ABT‐263 overcomes hypoxia‐driven radioresistence and improves radiotherapy. Cell Death Dis. 2021;12(7):694. 34257274 10.1038/s41419-021-03971-7 PMC8277842

[220] Roberts AW , Seymour JF , Brown JR , et al. Substantial susceptibility of chronic lymphocytic leukemia to BCL2 inhibition: results of a phase I study of navitoclax in patients with relapsed or refractory disease. J Clin Oncol. 2012;30(5):488‐496. 22184378 10.1200/JCO.2011.34.7898 PMC4979082

[221] Kipps TJ , Eradat H , Grosicki S , et al. A phase 2 study of the BH3 mimetic BCL2 inhibitor navitoclax (ABT‐263) with or without rituximab, in previously untreated B‐cell chronic lymphocytic leukemia. Leuk Lymphoma. 2015;56(10):2826‐2833. 25797560 10.3109/10428194.2015.1030638 PMC4643417

[222] Bose P , Gandhi V , Konopleva M . Pathways and mechanisms of venetoclax resistance. Leuk Lymphoma. 2017;58(9):1‐17. 10.1080/10428194.2017.1283032 PMC5478500 28140720

[223] Salimi‐Jeda A , Ghabeshi S , Gol Mohammad Pour Z , et al. Autophagy modulation and cancer combination therapy: a smart approach in cancer therapy. Cancer Treat Res Commun. 2022;30:100512. 35026533 10.1016/j.ctarc.2022.100512

[224] Liu X‐L , Ding J , Meng L‐H . Oncogene‐induced senescence: a double edged sword in cancer. Acta Pharmacol Sin. 2018;39(10):1553‐1558. 29620049 10.1038/aps.2017.198 PMC6289471

[225] Lorente J , Velandia C , Leal JA , et al. The interplay between autophagy and tumorigenesis: exploiting autophagy as a means of anticancer therapy. Biol Rev Camb Philos Soc. 2018;93(1):152‐165. 28464404 10.1111/brv.12337

[226] Yang ZJ , Chee CE , Huang S , Sinicrope FA . The role of autophagy in cancer: therapeutic implications. Mol Cancer Ther. 2011;10(9):1533‐1541. 21878654 10.1158/1535-7163.MCT-11-0047 PMC3170456

[227] Wang J , Wu GS . Role of autophagy in cisplatin resistance in ovarian cancer cells. J Biol Chem. 2014;289(24):17163‐17173. 24794870 10.1074/jbc.M114.558288 PMC4059157

[228] Castro I , Sampaio‐Marques B , Ludovico P . Targeting metabolic reprogramming in acute myeloid leukemia. Cells. 2019;8(9):967. 31450562 10.3390/cells8090967 PMC6770240

[229] Wong MM‐T , Chan H‐Y , Aziz NA , et al. Interplay of autophagy and cancer stem cells in hepatocellular carcinoma. Mol Biol Rep. 2021;48(4):3695‐3717. 33893928 10.1007/s11033-021-06334-9

[230] Wen Y‐A , Xing X , Harris JW , et al. Adipocytes activate mitochondrial fatty acid oxidation and autophagy to promote tumor growth in colon cancer. Cell Death Dis. 2017;8(2):e2593. 28151470 10.1038/cddis.2017.21 PMC5386470

[231] Amaravadi RK , Yu D , Lum JJ , et al. Autophagy inhibition enhances therapy‐induced apoptosis in a Myc‐induced model of lymphoma. J Clin Invest. 2007;117(2):326‐336. 17235397 10.1172/JCI28833 PMC1765515

[232] Han W , Pan H , Chen Y , et al. EGFR tyrosine kinase inhibitors activate autophagy as a cytoprotective response in human lung cancer cells. PLoS One. 2011;6(6):e18691. 21655094 10.1371/journal.pone.0018691 PMC3107207

[233] Gupta A , Roy S , Lazar AJF , et al. Autophagy inhibition and antimalarials promote cell death in gastrointestinal stromal tumor (GIST). Proc Natl Acad Sci USA. 2010;107(32):14333‐14338. 20660757 10.1073/pnas.1000248107 PMC2922542

[234] Bellodi C , Lidonnici MR , Hamilton A , et al. Targeting autophagy potentiates tyrosine kinase inhibitor‐induced cell death in Philadelphia chromosome‐positive cells, including primary CML stem cells. J Clin Invest. 2009;119(5):1109‐1123. 19363292 10.1172/JCI35660 PMC2673867

[235] Dixon SJ , Lemberg KM , Lamprecht MR , et al. Ferroptosis: an iron‐dependent form of nonapoptotic cell death. Cell. 2012;149(5):1060‐1072. 22632970 10.1016/j.cell.2012.03.042 PMC3367386

[236] Zhang C , Liu X , Jin S , Chen Y , Guo R . Ferroptosis in cancer therapy: a novel approach to reversing drug resistance. Mol Cancer. 2022;21(1):47. 35151318 10.1186/s12943-022-01530-y PMC8840702

[237] Yang WS , SriRamaratnam R , Welsch ME , et al. Regulation of ferroptotic cancer cell death by GPX4. Cell. 2014;156(1‐2):317‐331. 24439385 10.1016/j.cell.2013.12.010 PMC4076414

[238] Forcina GC , Dixon SJ . GPX4 at the crossroads of lipid homeostasis and ferroptosis. Proteomics. 2019;19(18):e1800311. 30888116 10.1002/pmic.201800311

[239] Chen T‐C , Chuang J‐Y , Ko C‐Y , et al. AR ubiquitination induced by the curcumin analog suppresses growth of temozolomide‐resistant glioblastoma through disrupting GPX4‐Mediated redox homeostasis. Redox Biol. 2020;30:101413. 31896509 10.1016/j.redox.2019.101413 PMC6940696

[240] Yang C , Zhang Y , Lin S , Liu Y , Li W . Suppressing the KIF20A/NUAK1/Nrf2/GPX4 signaling pathway induces ferroptosis and enhances the sensitivity of colorectal cancer to oxaliplatin. Aging (Albany NY). 2021;13(10):13515‐13534. 33819186 10.18632/aging.202774 PMC8202845

[241] Roh J‐L , Kim EH , Jang HJ , Park JY , Shin D . Induction of ferroptotic cell death for overcoming cisplatin resistance of head and neck cancer. Cancer Lett. 2016;381(1):96‐103. 27477897 10.1016/j.canlet.2016.07.035

[242] Fu D , Wang C , Yu L , Yu R . Induction of ferroptosis by ATF3 elevation alleviates cisplatin resistance in gastric cancer by restraining Nrf2/Keap1/xCT signaling. Cell Mol Biol Lett. 2021;26(1):26. 34098867 10.1186/s11658-021-00271-y PMC8186082

[243] Du J , Wang X , Li Y , et al. DHA exhibits synergistic therapeutic efficacy with cisplatin to induce ferroptosis in pancreatic ductal adenocarcinoma via modulation of iron metabolism. Cell Death Dis. 2021;12(7):705. 34262021 10.1038/s41419-021-03996-y PMC8280115

[244] Turcu AL , Versini A , Khene N , et al. DMT1 inhibitors kill cancer stem cells by blocking lysosomal iron translocation. Chemistry. 2020;26(33):7369‐7373. 32083771 10.1002/chem.202000159

[245] Chaudhary N , Choudhary BS , Shah SG , et al. Lipocalin 2 expression promotes tumor progression and therapy resistance by inhibiting ferroptosis in colorectal cancer. Int J Cancer. 2021;149(7):1495‐1511. 34146401 10.1002/ijc.33711

[246] Doll S , Proneth B , Tyurina YY , et al. ACSL4 dictates ferroptosis sensitivity by shaping cellular lipid composition. Nat Chem Biol. 2017;13(1):91‐98. 27842070 10.1038/nchembio.2239 PMC5610546

[247] Ye Z , Hu Q , Zhuo Q , et al. Abrogation of ARF6 promotes RSL3‐induced ferroptosis and mitigates gemcitabine resistance in pancreatic cancer cells. Am J Cancer Res. 2020;10(4):1182‐1193. 32368394 PMC7191101

[248] Tsvetkov P , Coy S , Petrova B , et al. Copper induces cell death by targeting lipoylated TCA cycle proteins. Science. 2022;375(6586):1254‐1261. 35298263 10.1126/science.abf0529 PMC9273333

[249] Mayr JA , Feichtinger RG , Tort F , Ribes A , Sperl W . Lipoic acid biosynthesis defects. J Inherit Metab Dis. 2014;37(4):553‐563. 24777537 10.1007/s10545-014-9705-8

[250] Solmonson A , DeBerardinis RJ . Lipoic acid metabolism and mitochondrial redox regulation. J Biol Chem. 2018;293(20):7522‐7530. 29191830 10.1074/jbc.TM117.000259 PMC5961061

[251] Gaggelli E , Kozlowski H , Valensin D , Valensin G . Copper homeostasis and neurodegenerative disorders (Alzheimer's, prion, and Parkinson's diseases and amyotrophic lateral sclerosis). Chem Rev. 2006;106(6):1995‐2044. 16771441 10.1021/cr040410w

[252] Bandmann O , Weiss KH , Kaler SG . Wilson's disease and other neurological copper disorders. Lancet Neurol. 2015;14(1):103‐113. 25496901 10.1016/S1474-4422(14)70190-5 PMC4336199

[253] Tsang T , Posimo JM , Gudiel AA , Cicchini M , Feldser DM , Brady DC . Copper is an essential regulator of the autophagic kinases ULK1/2 to drive lung adenocarcinoma. Nat Cell Biol. 2020;22(4):412‐424. 32203415 10.1038/s41556-020-0481-4 PMC7610258

[254] Cui L , Gouw AM , LaGory EL , et al. Mitochondrial copper depletion suppresses triple‐negative breast cancer in mice. Nat Biotechnol. 2021;39(3):357‐367. 33077961 10.1038/s41587-020-0707-9 PMC7956242

[255] Aggarwal A , Bhatt M . Advances in treatment of Wilson disease. Tremor Other Hyperkinet Mov (NY). 2018;8:525. 10.7916/D841881D PMC5840318 29520330

[256] O'Day SJ , Eggermont AMM , Chiarion‐Sileni V , et al. Final results of phase III SYMMETRY study: randomized, double‐blind trial of elesclomol plus paclitaxel versus paclitaxel alone as treatment for chemotherapy‐naive patients with advanced melanoma. J Clin Oncol. 2013;31(9):1211‐1218. 23401447 10.1200/JCO.2012.44.5585

[257] Tang D , Chen X , Kroemer G . Cuproptosis: a copper‐triggered modality of mitochondrial cell death. Cell Res. 2022;32(5):417‐418. 35354936 10.1038/s41422-022-00653-7 PMC9061796

[258] Vasan N , Baselga J , Hyman DM . A view on drug resistance in cancer. Nature. 2019;575(7782):299‐309. 31723286 10.1038/s41586-019-1730-1 PMC8008476

[259] Lambrechts D , Wauters E , Boeckx B , et al. Phenotype molding of stromal cells in the lung tumor microenvironment. Nat Med. 2018;24(8):1277‐1289. 29988129 10.1038/s41591-018-0096-5

[260] Burgos‐Panadero R , Lucantoni F , Gamero‐Sandemetrio E , de la Cruz‐Merino L , Alvaro T , Noguera R . The tumour microenvironment as an integrated framework to understand cancer biology. Cancer Lett. 2019;461:112‐122. 31325528 10.1016/j.canlet.2019.07.010

[261] Leroy J , Jamali F , Forbes L , et al. Laparoscopic total mesorectal excision (TME) for rectal cancer surgery: long‐term outcomes. Surg Endosc. 2004;18(2):281‐289. 14691716 10.1007/s00464-002-8877-8

[262] Henke E , Nandigama R , Ergun S . Extracellular matrix in the tumor microenvironment and its impact on cancer therapy. Front Mol Biosci. 2019;6:160. 32118030 10.3389/fmolb.2019.00160 PMC7025524

[263] Yu Q , Xiao W , Sun S , Sohrabi A , Liang J , Seidlits SK . Extracellular matrix proteins confer cell adhesion‐mediated drug resistance through integrin α v in glioblastoma cells. Front Cell Dev Biol. 2021;9:616580. 33834020 10.3389/fcell.2021.616580 PMC8021872

[264] Misra S , Ghatak S , Zoltan‐Jones A , Toole BP . Regulation of multidrug resistance in cancer cells by hyaluronan. J Biol Chem. 2003;278(28):25285‐25288. 12738783 10.1074/jbc.C300173200

[265] Park CC , Zhang HJ , Yao ES , Park CJ , Bissell MJ . Beta1 integrin inhibition dramatically enhances radiotherapy efficacy in human breast cancer xenografts. Cancer Res. 2008;68(11):4398‐4405. 18519702 10.1158/0008-5472.CAN-07-6390 PMC3719863

[266] Weigelt B , Lo AT , Park CC , Gray JW , Bissell MJ . HER2 signaling pathway activation and response of breast cancer cells to HER2‐targeting agents is dependent strongly on the 3D microenvironment. Breast Cancer Res Treat. 2010;122(1):35‐43. 19701706 10.1007/s10549-009-0502-2 PMC2935800

[267] Zigrino P , Nischt R , Mauch C . The disintegrin‐like and cysteine‐rich domains of ADAM‐9 mediate interactions between melanoma cells and fibroblasts. J Biol Chem. 2011;286(8):6801‐6807. 21135106 10.1074/jbc.M110.168617 PMC3057808

[268] de la Fuente MT , Casanova B , Moyano JV , et al. Engagement of alpha4beta1 integrin by fibronectin induces in vitro resistance of B chronic lymphocytic leukemia cells to fludarabine. J Leukoc Biol. 2002;71(3):495‐502. 11867687

[269] Matsunaga T , Takemoto N , Sato T , et al. CORRIGENDUM: interaction between leukemic‐cell VLA‐4 and stromal fibronectin is a decisive factor for minimal residual disease of acute myelogenous leukemia. Nat Med. 2005;11(5):578. 10.1038/nm909 12897778

[270] Nishio M , Endo T , Tsukada N , et al. Nurselike cells express BAFF and APRIL, which can promote survival of chronic lymphocytic leukemia cells via a paracrine pathway distinct from that of SDF‐1alpha. Blood. 2005;106(3):1012‐1020. 15860672 10.1182/blood-2004-03-0889 PMC1895149

[271] Chen Y , Jacamo R , Konopleva M , Garzon R , Croce C , Andreeff M . CXCR4 downregulation of let‐7a drives chemoresistance in acute myeloid leukemia. J Clin Invest. 2013;123(6):2395‐2407. 23676502 10.1172/JCI66553 PMC3668829

[272] Margolin DA , Silinsky J , Grimes C , et al. Lymph node stromal cells enhance drug‐resistant colon cancer cell tumor formation through SDF‐1alpha/CXCR4 paracrine signaling. Neoplasia. 2011;13(9):874‐886. 21969820 10.1593/neo.11324 PMC3182279

[273] Azmi AS , Bao B , Sarkar FH . Exosomes in cancer development, metastasis, and drug resistance: a comprehensive review. Cancer Metastasis Rev. 2013;32(3‐4):623‐642. 23709120 10.1007/s10555-013-9441-9 PMC3843988

[274] Colombo M , Raposo G , Thery C . Biogenesis, secretion, and intercellular interactions of exosomes and other extracellular vesicles. Annu Rev Cell Dev Biol. 2014;30:255‐289. 25288114 10.1146/annurev-cellbio-101512-122326

[275] Valadi H , Ekstrom K , Bossios A , Sjostrand M , Lee JJ , Lotvall JO . Exosome‐mediated transfer of mRNAs and microRNAs is a novel mechanism of genetic exchange between cells. Nat Cell Biol. 2007;9(6):654‐659. 17486113 10.1038/ncb1596

[276] Mathivanan S , Ji H , Simpson RJ . Exosomes: extracellular organelles important in intercellular communication. J Proteomics. 2010;73(10):1907‐1920. 20601276 10.1016/j.jprot.2010.06.006

[277] D'Asti E , Garnier D , Lee TH , Montermini L , Meehan B , Rak J . Oncogenic extracellular vesicles in brain tumor progression. Front Physiol. 2012;3:294. 22934045 10.3389/fphys.2012.00294 PMC3429065

[278] Zhang H‐G , Grizzle WE . Exosomes: a novel pathway of local and distant intercellular communication that facilitates the growth and metastasis of neoplastic lesions. Am J Pathol. 2014;184(1):28‐41. 24269592 10.1016/j.ajpath.2013.09.027 PMC3873490

[279] Quail DF , Joyce JA . Microenvironmental regulation of tumor progression and metastasis. Nat Med. 2013;19(11):1423‐1437. 24202395 10.1038/nm.3394 PMC3954707

[280] Deep G , Panigrahi GK . Hypoxia‐induced signaling promotes prostate cancer progression: exosomes role as messenger of hypoxic response in tumor microenvironment. Crit Rev Oncog. 2015;20(5‐6):419‐434. 27279239 10.1615/CritRevOncog.v20.i5-6.130 PMC5308872

[281] Webber J , Steadman R , Mason MD , Tabi Z , Clayton A . Cancer exosomes trigger fibroblast to myofibroblast differentiation. Cancer Res. 2010;70(23):9621‐9630. 21098712 10.1158/0008-5472.CAN-10-1722

[282] Zhang Q , Peng C . Cancer‐associated fibroblasts regulate the biological behavior of cancer cells and stroma in gastric cancer. Oncol Lett. 2018;15(1):691‐698. 29399141 10.3892/ol.2017.7385 PMC5772670

[283] Richards KE , Zeleniak AE , Fishel ML , Wu J , Littlepage LE , Hill R . Cancer‐associated fibroblast exosomes regulate survival and proliferation of pancreatic cancer cells. Oncogene. 2017;36(13):1770‐1778. 27669441 10.1038/onc.2016.353 PMC5366272

[284] Shedden K , Xie XT , Chandaroy P , Chang YT , Rosania GR . Expulsion of small molecules in vesicles shed by cancer cells: association with gene expression and chemosensitivity profiles. Cancer Res. 2003;63(15):4331‐4337. 12907600

[285] Corrado C , Raimondo S , Chiesi A , Ciccia F , De Leo G , Alessandro R . Exosomes as intercellular signaling organelles involved in health and disease: basic science and clinical applications. Int J Mol Sci. 2013;14(3):5338‐5366. 23466882 10.3390/ijms14035338 PMC3634447

[286] Maleki S , Jabalee J , Garnis C . The role of extracellular vesicles in mediating resistance to anticancer therapies. Int J Mol Sci. 2021;22(8):4166. 33920605 10.3390/ijms22084166 PMC8073860

[287] Muralidharan‐Chari V , Kohan HG , Asimakopoulos AG , et al. Microvesicle removal of anticancer drugs contributes to drug resistance in human pancreatic cancer cells. Oncotarget. 2016;7(31):50365‐50379. 27391262 10.18632/oncotarget.10395 PMC5226588

[288] Ning K , Wang T , Sun X , et al. UCH‐L1‐containing exosomes mediate chemotherapeutic resistance transfer in breast cancer. J Surg Oncol. 2017;115(8):932‐940. 28334432 10.1002/jso.24614

[289] Crow J , Atay S , Banskota S , Artale B , Schmitt S , Godwin AK . Exosomes as mediators of platinum resistance in ovarian cancer. Oncotarget. 2017;8(7):11917‐11936. 28060758 10.18632/oncotarget.14440 PMC5355315

[290] Han M , Hu J , Lu P , et al. Exosome‐transmitted miR‐567 reverses trastuzumab resistance by inhibiting ATG5 in breast cancer. Cell Death Dis. 2020;11(1):43. 31969559 10.1038/s41419-020-2250-5 PMC6976584

[291] Santos JC , Lima NdS , Sarian LO , Matheu A , Ribeiro ML , Derchain SFM . Exosome‐mediated breast cancer chemoresistance via miR‐155 transfer. Sci Rep. 2018;8(1):829. 29339789 10.1038/s41598-018-19339-5 PMC5770414

[292] Dong H , Wang W , Chen R , et al. Exosome‐mediated transfer of lncRNA‑SNHG14 promotes trastuzumab chemoresistance in breast cancer. Int J Oncol. 2018;53(3):1013‐1026. 30015837 10.3892/ijo.2018.4467 PMC6065402

[293] Jing C , Cao H , Qin X , et al. Exosome‐mediated gefitinib resistance in lung cancer HCC827 cells via delivery of miR‐21. Oncol Lett. 2018;15(6):9811‐9817. 29928355 10.3892/ol.2018.8604 PMC6004702

[294] Fu X , Liu M , Qu S , et al. Exosomal microRNA‐32‐5p induces multidrug resistance in hepatocellular carcinoma via the PI3K/Akt pathway. J Exp Clin Cancer Res. 2018;37(1):52. 29530052 10.1186/s13046-018-0677-7 PMC5846230

[295] Fornari F , Pollutri D , Patrizi C , et al. In hepatocellular carcinoma miR‐221 modulates sorafenib resistance through inhibition of caspase‐3‐mediated apoptosis. Clin Cancer Res. 2017;23(14):3953‐3965. 28096271 10.1158/1078-0432.CCR-16-1464

[296] Hicklin DJ , Ellis LM . Role of the vascular endothelial growth factor pathway in tumor growth and angiogenesis. J Clin Oncol. 2005;23(5):1011‐1027. 15585754 10.1200/JCO.2005.06.081

[297] Folkman J . Tumor angiogenesis: therapeutic implications. N Engl J Med. 1971;285(21):1182‐1186. 4938153 10.1056/NEJM197111182852108

[298] Liang H , Wang M . Prospect of immunotherapy combined with anti‐angiogenic agents in patients with advanced non‐small cell lung cancer. Cancer Manag Res. 2019;11:7707‐7719. 31616186 10.2147/CMAR.S212238 PMC6699593

[299] Gacche RN . Compensatory angiogenesis and tumor refractoriness. Oncogenesis. 2015;4(6):e153. 26029827 10.1038/oncsis.2015.14 PMC4753522

[300] Jayson GC , Kerbel R , Ellis LM , Harris AL . Antiangiogenic therapy in oncology: current status and future directions. Lancet. 2016;388(10043):518‐529. 26853587 10.1016/S0140-6736(15)01088-0

[301] Gacche RN , Assaraf YG . Redundant angiogenic signaling and tumor drug resistance. Drug Resist Updat. 2018;36:47‐76. 29499837 10.1016/j.drup.2018.01.002

[302] Ribatti D . The chick embryo chorioallantoic membrane (CAM). A multifaceted experimental model. Mech Dev. 2016;141:70‐77. 27178379 10.1016/j.mod.2016.05.003

[303] Lohela M , Bry M , Tammela T , Alitalo K . VEGFs and receptors involved in angiogenesis versus lymphangiogenesis. Curr Opin Cell Biol. 2009;21(2):154‐165. 19230644 10.1016/j.ceb.2008.12.012

[304] Ellis LM , Hicklin DJ . Pathways mediating resistance to vascular endothelial growth factor‐targeted therapy. Clin Cancer Res. 2008;14(20):6371‐6375. 18927275 10.1158/1078-0432.CCR-07-5287

[305] Crawford Y , Kasman I , Yu L , et al. PDGF‐C mediates the angiogenic and tumorigenic properties of fibroblasts associated with tumors refractory to anti‐VEGF treatment. Cancer Cell. 2009;15(1):21‐34. 19111878 10.1016/j.ccr.2008.12.004

[306] Vasudev NS , Reynolds AR . Anti‐angiogenic therapy for cancer: current progress, unresolved questions and future directions. Angiogenesis. 2014;17(3):471‐494. 24482243 10.1007/s10456-014-9420-y PMC4061466

[307] Bergers G , Song S . The role of pericytes in blood‐vessel formation and maintenance. Neuro Oncol. 2005;7(4):452‐464. 16212810 10.1215/S1152851705000232 PMC1871727

[308] Aldea M , Andre F , Marabelle A , Dogan S , Barlesi F , Soria J‐C . Overcoming resistance to tumor‐targeted and immune‐targeted therapies. Cancer Discov. 2021;11(4):874‐899. 33811122 10.1158/2159-8290.CD-20-1638

[309] Koyama S , Akbay EA , Li YY , et al. Adaptive resistance to therapeutic PD‐1 blockade is associated with upregulation of alternative immune checkpoints. Nat Commun. 2016;7(1):10501. 26883990 10.1038/ncomms10501 PMC4757784

[310] Saleh R , Elkord E . Treg‐mediated acquired resistance to immune checkpoint inhibitors. Cancer Lett. 2019;457:168‐179. 31078738 10.1016/j.canlet.2019.05.003

[311] Jenkins L , Jungwirth U , Avgustinova A , et al. Cancer‐associated fibroblasts suppress CD8+ T‐cell infiltration and confer resistance to immune‐checkpoint blockade. Cancer Res. 2022;82(16):2904‐2917. 35749591 10.1158/0008-5472.CAN-21-4141 PMC9379365

[312] Deng L , Liang H , Burnette B , et al. Irradiation and anti‐PD‐L1 treatment synergistically promote antitumor immunity in mice. J Clin Invest. 2014;124(2):687‐695. 24382348 10.1172/JCI67313 PMC3904601

[313] Twyman‐Saint Victor C , Rech AJ , Maity A , et al. Radiation and dual checkpoint blockade activate non‐redundant immune mechanisms in cancer. Nature. 2015;520(7547):373‐377. 25754329 10.1038/nature14292 PMC4401634

[314] Galluzzi L , Humeau J , Buque A , Zitvogel L , Kroemer G . Immunostimulation with chemotherapy in the era of immune checkpoint inhibitors. Nat Rev Clin Oncol. 2020;17(12):725‐741. 32760014 10.1038/s41571-020-0413-z

[315] Tang T , Huang X , Zhang G , Hong Z , Bai X , Liang T . Advantages of targeting the tumor immune microenvironment over blocking immune checkpoint in cancer immunotherapy. Signal Transduct Target Ther. 2021;6(1):72. 33608497 10.1038/s41392-020-00449-4 PMC7896069

[316] Wallin JJ , Bendell JC , Funke R , et al. Atezolizumab in combination with bevacizumab enhances antigen‐specific T‐cell migration in metastatic renal cell carcinoma. Nat Commun. 2016;7(1):12624. 27571927 10.1038/ncomms12624 PMC5013615

[317] Casak SJ , Donoghue M , Fashoyin‐Aje L , et al. FDA approval summary: atezolizumab plus bevacizumab for the treatment of patients with advanced unresectable or metastatic hepatocellular carcinoma. Clin Cancer Res. 2021;27(7):1836‐1841. 33139264 10.1158/1078-0432.CCR-20-3407

[318] Harrington K , Freeman DJ , Kelly B , Harper J , Soria J‐C . Optimizing oncolytic virotherapy in cancer treatment. Nat Rev Drug Discov. 2019;18(9):689‐706. 31292532 10.1038/s41573-019-0029-0

[319] Martin NT , Bell JC . Oncolytic virus combination therapy: killing one bird with two stones. Mol Ther. 2018;26(6):1414‐1422. 29703699 10.1016/j.ymthe.2018.04.001 PMC5986726

[320] Bourgeois‐Daigneault M‐C , Roy DG , Aitken AS , et al. Neoadjuvant oncolytic virotherapy before surgery sensitizes triple‐negative breast cancer to immune checkpoint therapy. Sci Transl Med. 2018;10(422):eaao1641. 29298865 10.1126/scitranslmed.aao1641

[321] Li R , Zhang J , Gilbert SM , Conejo‐Garcia J , Mule JJ . Using oncolytic viruses to ignite the tumour immune microenvironment in bladder cancer. Nat Rev Urol. 2021;18(9):543‐555. 34183833 10.1038/s41585-021-00483-z

[322] Ribas A , Dummer R , Puzanov I , et al. Oncolytic virotherapy promotes intratumoral t cell infiltration and improves anti‐PD‐1 immunotherapy. Cell. 2017;170(6):1109‐1119.e10. 28886381 10.1016/j.cell.2017.08.027 PMC8034392

[323] Zhang X , Chen X , Guo Y , et al. Dual gate‐controlled therapeutics for overcoming bacterium‐induced drug resistance and potentiating cancer immunotherapy. Angew Chem Int Ed Engl. 2021;60(25):14013‐14021. 33768682 10.1002/anie.202102059

[324] Arthur JC , Perez‐Chanona E , Muhlbauer M , et al. Intestinal inflammation targets cancer‐inducing activity of the microbiota. Science. 2012;338(6103):120‐123. 22903521 10.1126/science.1224820 PMC3645302

[325] Dejea CM , Fathi P , Craig JM , et al. Patients with familial adenomatous polyposis harbor colonic biofilms containing tumorigenic bacteria. Science. 2018;359(6375):592‐597. 29420293 10.1126/science.aah3648 PMC5881113

[326] Zheng D‐W , Dong X , Pan P , et al. Phage‐guided modulation of the gut microbiota of mouse models of colorectal cancer augments their responses to chemotherapy. Nat Biomed Eng. 2019;3(9):717‐728. 31332342 10.1038/s41551-019-0423-2

[327] Han H , Hou Y , Chen X , et al. Metformin‐induced stromal depletion to enhance the penetration of gemcitabine‐loaded magnetic nanoparticles for pancreatic cancer targeted therapy. J Am Chem Soc. 2020;142(10):4944‐4954. 32069041 10.1021/jacs.0c00650

[328] Geller LT , Barzily‐Rokni M , Danino T , et al. Potential role of intratumor bacteria in mediating tumor resistance to the chemotherapeutic drug gemcitabine. Science. 2017;357(6356):1156‐1160. 28912244 10.1126/science.aah5043 PMC5727343

[329] Dong X , Pan P , Zheng D‐W , Bao P , Zeng X , Zhang X‐Z . Bioinorganic hybrid bacteriophage for modulation of intestinal microbiota to remodel tumor‐immune microenvironment against colorectal cancer. Sci Adv. 2020;6(20):eaba1590. 32440552 10.1126/sciadv.aba1590 PMC7228756

[330] Iida N , Dzutsev A , Stewart CA , et al. Commensal bacteria control cancer response to therapy by modulating the tumor microenvironment. Science. 2013;342(6161):967‐970. 24264989 10.1126/science.1240527 PMC6709532

[331] Pernigoni N , Zagato E , Calcinotto A , et al. Commensal bacteria promote endocrine resistance in prostate cancer through androgen biosynthesis. Science. 2021;374(6564):216‐224. 34618582 10.1126/science.abf8403

[332] Zhu Y‐X , Jia H‐R , Duan Q‐Y , Wu F‐G . Nanomedicines for combating multidrug resistance of cancer. Wiley Interdiscip Rev Nanomed Nanobiotechnol. 2021;13(5):e1715. 33860622 10.1002/wnan.1715

[333] Xiong M‐H , Bao Y , Du X‐J , et al. Differential anticancer drug delivery with a nanogel sensitive to bacteria‐accumulated tumor artificial environment. ACS Nano. 2013;7(12):10636‐10645. 24200225 10.1021/nn403146t

[334] Lawson DA , Bhakta NR , Kessenbrock K , et al. Single‐cell analysis reveals a stem‐cell program in human metastatic breast cancer cells. Nature. 2015;526(7571):131‐135. 26416748 10.1038/nature15260 PMC4648562

[335] Mani SA , Guo W , Liao M‐J , et al. The epithelial‐mesenchymal transition generates cells with properties of stem cells. Cell. 2008;133(4):704‐715. 18485877 10.1016/j.cell.2008.03.027 PMC2728032

[336] Shimokawa M , Ohta Y , Nishikori S , et al. Visualization and targeting of LGR5(+) human colon cancer stem cells. Nature. 2017;545(7653):187‐192. 28355176 10.1038/nature22081

[337] Soliman AA , Elzarkaa AA , Malik E . Epithelial ovarian cancer and cancer stem cells. Adv Exp Med Biol. 2021;1330:21‐32. 34339028 10.1007/978-3-030-73359-9_2

[338] Zhou H‐M , Zhang J‐G , Zhang X , Li Q . Targeting cancer stem cells for reversing therapy resistance: mechanism, signaling, and prospective agents. Signal Transduct Target Ther. 2021;6(1):62. 33589595 10.1038/s41392-020-00430-1 PMC7884707

[339] Wang H , Mei Y , Luo C , et al. Single‐cell analyses reveal mechanisms of cancer stem cell maintenance and epithelial‐mesenchymal transition in recurrent bladder cancer. Clin Cancer Res. 2021;27(22):6265‐6278. 34526362 10.1158/1078-0432.CCR-20-4796

[340] Shi Z‐D , Hao L , Han X‐X , et al. Targeting HNRNPU to overcome cisplatin resistance in bladder cancer. Mol Cancer. 2022;21(1):37. 35130920 10.1186/s12943-022-01517-9 PMC8819945

[341] He X , Smith SE , Chen S , et al. Tumor‐initiating stem cell shapes its microenvironment into an immunosuppressive barrier and pro‐tumorigenic niche. Cell Rep. 2021;36(10):109674. 34496236 10.1016/j.celrep.2021.109674 PMC8451448

[342] Wang D , Fu L , Sun H , Guo L , DuBois RN . Prostaglandin E2 promotes colorectal cancer stem cell expansion and metastasis in mice. Gastroenterology. 2015;149(7):1884‐1895.e4. 26261008 10.1053/j.gastro.2015.07.064 PMC4762503

[343] Sun L , Huang C , Zhu M , et al. Gastric cancer mesenchymal stem cells regulate PD‐L1‐CTCF enhancing cancer stem cell‐like properties and tumorigenesis. Theranostics. 2020;10(26):11950‐11962. 33204322 10.7150/thno.49717 PMC7667687

[344] Wei F , Zhang T , Deng S‐C , et al. PD‐L1 promotes colorectal cancer stem cell expansion by activating HMGA1‐dependent signaling pathways. Cancer Lett. 2019;450:1‐13. 30776481 10.1016/j.canlet.2019.02.022

[345] Ouadah Y , Rojas ER , Riordan DP , Capostagno S , Kuo CS , Krasnow MA . Rare pulmonary neuroendocrine cells are stem cells regulated by Rb, p53, and notch. Cell. 2019;179(2):403‐416.e23. 31585080 10.1016/j.cell.2019.09.010 PMC6782070

[346] Novellasdemunt L , Kucharska A , Jamieson C , et al. NEDD4 and NEDD4L regulate Wnt signalling and intestinal stem cell priming by degrading LGR5 receptor. EMBO J. 2020;39(3):e102771. 31867777 10.15252/embj.2019102771 PMC6996568

[347] Zhang L , He X , Liu X , et al. Single‐cell transcriptomics in medulloblastoma reveals tumor‐initiating progenitors and oncogenic cascades during tumorigenesis and relapse. Cancer Cell. 2019;36(3):302‐318.e7. 31474569 10.1016/j.ccell.2019.07.009 PMC6760242

[348] Deslauriers AG , Kotini AG , Papapetrou EP . Modeling leukemia stem cells with patient‐derived induced pluripotent stem cells. Methods Mol Biol. 2021;2185:411‐422. 33165864 10.1007/978-1-0716-0810-4_26 PMC7821974

[349] Chaffer CL , San Juan BP , Lim E , Weinberg RA . EMT, cell plasticity and metastasis. Cancer Metastasis Rev. 2016;35(4):645‐654. 27878502 10.1007/s10555-016-9648-7

[350] Na T‐Y , Schecterson L , Mendonsa AM , Gumbiner BM . The functional activity of E‐cadherin controls tumor cell metastasis at multiple steps. Proc Natl Acad Sci USA. 2020;117(11):5931‐5937. 32127478 10.1073/pnas.1918167117 PMC7084067

[351] Han LL , Jia L , Wu F , Huang C . Sirtuin6 (SIRT6) promotes the EMT of hepatocellular carcinoma by stimulating autophagic degradation of E‐cadherin. Mol Cancer Res. 2019;17(11):2267‐2280. 31551254 10.1158/1541-7786.MCR-19-0321

[352] Zhang J , Cai H , Sun L , et al. LGR5, a novel functional glioma stem cell marker, promotes EMT by activating the Wnt/beta‐catenin pathway and predicts poor survival of glioma patients. J Exp Clin Cancer Res. 2018;37(1):225. 30208924 10.1186/s13046-018-0864-6 PMC6136228

[353] Tang Q , Chen J , Di Z , et al. TM4SF1 promotes EMT and cancer stemness via the Wnt/beta‐catenin/SOX2 pathway in colorectal cancer. J Exp Clin Cancer Res. 2020;39(1):232. 33153498 10.1186/s13046-020-01690-z PMC7643364

[354] Zhang N , Hua X , Tu H , Li J , Zhang Z , Max C . Isorhapontigenin (ISO) inhibits EMT through FOXO3A/METTL14/VIMENTIN pathway in bladder cancer cells. Cancer Lett. 2021;520:400‐408. 34332039 10.1016/j.canlet.2021.07.041 PMC9161647

[355] Tian H , Lian R , Li Y , et al. AKT‐induced lncRNA VAL promotes EMT‐independent metastasis through diminishing Trim16‐dependent Vimentin degradation. Nat Commun. 2020;11(1):5127. 33046716 10.1038/s41467-020-18929-0 PMC7550350

[356] Luo J , Yao J‐F , Deng X‐F , et al. 14, 15‐EET induces breast cancer cell EMT and cisplatin resistance by up‐regulating integrin alphavbeta3 and activating FAK/PI3K/AKT signaling. J Exp Clin Cancer Res. 2018;37(1):23. 29426357 10.1186/s13046-018-0694-6 PMC5807756

[357] Kim TW , Lee YS , Yun NH , et al. MicroRNA‐17‐5p regulates EMT by targeting Vimentin in colorectal cancer. Br J Cancer. 2020;123(7):1123‐1130. 32546833 10.1038/s41416-020-0940-5 PMC7524803

[358] Sinha S , Pattnaik S , Aradhyam GK . Molecular evolution guided functional analyses reveals nucleobindin‐1 as a canonical E‐box binding protein promoting epithelial‐to‐mesenchymal transition (EMT). Biochim Biophys Acta: Proteins Proteomics. 2019;1867(9):765‐775. 31176806 10.1016/j.bbapap.2019.05.009

[359] Liang S‐Q , Marti TM , Dorn P , et al. Blocking the epithelial‐to‐mesenchymal transition pathway abrogates resistance to anti‐folate chemotherapy in lung cancer. Cell Death Dis. 2015;6(7):e1824. 26181204 10.1038/cddis.2015.195 PMC4650740

[360] Torzilli PA , Bourne JW , Cigler T , Vincent CT . A new paradigm for mechanobiological mechanisms in tumor metastasis. Semin Cancer Biol. 2012;22(5‐6):385‐395. 22613484 10.1016/j.semcancer.2012.05.002 PMC3445741

[361] Zhu Y , Tan J , Xie H , Wang J , Meng X , Wang R . HIF‐1alpha regulates EMT via the Snail and beta‐catenin pathways in paraquat poisoning‐induced early pulmonary fibrosis. J Cell Mol Med. 2016;20(4):688‐697. 26781174 10.1111/jcmm.12769 PMC5126389

[362] Zhang P , Sun Y , Ma L . ZEB1: at the crossroads of epithelial‐mesenchymal transition, metastasis and therapy resistance. Cell Cycle. 2015;14(4):481‐487. 25607528 10.1080/15384101.2015.1006048 PMC4614883

[363] Xu Y , Lee D‐K , Feng Z , et al. Breast tumor cell‐specific knockout of Twist1 inhibits cancer cell plasticity, dissemination, and lung metastasis in mice. Proc Natl Acad Sci USA. 2017;114(43):11494‐11499. 29073077 10.1073/pnas.1618091114 PMC5664488

[364] Zhang W , Feng M , Zheng G , et al. Chemoresistance to 5‐fluorouracil induces epithelial‐mesenchymal transition via up‐regulation of Snail in MCF7 human breast cancer cells. Biochem Biophys Res Commun. 2012;417(2):679‐685. 22166209 10.1016/j.bbrc.2011.11.142

[365] Wang H , Zhang G , Zhang H , et al. Acquisition of epithelial‐mesenchymal transition phenotype and cancer stem cell‐like properties in cisplatin‐resistant lung cancer cells through AKT/beta‐catenin/Snail signaling pathway. Eur J Pharmacol. 2014;723:156‐166. 24333218 10.1016/j.ejphar.2013.12.004

[366] Namba T , Kodama R , Moritomo S , Hoshino T , Mizushima T . Zidovudine, an anti‐viral drug, resensitizes gemcitabine‐resistant pancreatic cancer cells to gemcitabine by inhibition of the Akt‐GSK3beta‐Snail pathway. Cell Death Dis. 2015;6(6):e1795. 26111057 10.1038/cddis.2015.172 PMC4669843

[367] Fischer KR , Durrans A , Lee S , et al. Epithelial‐to‐mesenchymal transition is not required for lung metastasis but contributes to chemoresistance. Nature. 2015;527(7579):472‐476. 26560033 10.1038/nature15748 PMC4662610

[368] Jin H , He Y , Zhao P , et al. Targeting lipid metabolism to overcome EMT‐associated drug resistance via integrin beta3/FAK pathway and tumor‐associated macrophage repolarization using legumain‐activatable delivery. Theranostics. 2019;9(1):265‐278. 30662566 10.7150/thno.27246 PMC6332796

[369] Raoof S , Mulford IJ , Frisco‐Cabanos H , et al. Targeting FGFR overcomes EMT‐mediated resistance in EGFR mutant non‐small cell lung cancer. Oncogene. 2019;38(37):6399‐6413. 31324888 10.1038/s41388-019-0887-2 PMC6742540

[370] Sheng W , Chen C , Dong M , et al. Calreticulin promotes EGF‐induced EMT in pancreatic cancer cells via Integrin/EGFR‐ERK/MAPK signaling pathway. Cell Death Dis. 2017;8(10):e3147. 29072694 10.1038/cddis.2017.547 PMC5680916

[371] Wu Q , Ma J , Wei J , Meng W , Wang Y , Shi M . lncRNA SNHG11 promotes gastric cancer progression by activating the Wnt/beta‐catenin pathway and oncogenic autophagy. Mol Ther. 2021;29(3):1258‐1278. 33068778 10.1016/j.ymthe.2020.10.011 PMC7934455

[372] Cheng F , Shen Y , Mohanasundaram P , et al. Vimentin coordinates fibroblast proliferation and keratinocyte differentiation in wound healing via TGF‐beta‐Slug signaling. Proc Natl Acad Sci USA. 2016;113(30):E4320‐E4327. 27466403 10.1073/pnas.1519197113 PMC4968728

[373] Kariya Y , Oyama M , Suzuki T , Kariya Y . Alphavbeta3 integrin induces partial EMT independent of TGF‐beta signaling. Commun Biol. 2021;4(1):490. 33883697 10.1038/s42003-021-02003-6 PMC8060333

[374] Gachpazan M , Kashani H , Hassanian SM , et al. Therapeutic potential of targeting transforming growth factor‐beta in colorectal cancer: rational and progress. Curr Pharm Des. 2019;25(38):4085‐4089. 31692434 10.2174/1381612825666191105114539

[375] Katoh M , Katoh M . Molecular genetics and targeted therapy of WNT‐related human diseases (Review). Int J Mol Med. 2017;40(3):587‐606. 28731148 10.3892/ijmm.2017.3071 PMC5547940

[376] Alavijeh MS , Palmer AM . The pivotal role of drug metabolism and pharmacokinetics in the discovery and development of new medicines. Curr Opin Investig Drugs. 2004;7(8):755‐763. 15334309

[377] Palmer AM . New horizons in drug metabolism, pharmacokinetics and drug discovery. Drug News Perspect. 2003;16(1):57‐62. 12682673

[378] Sacks LV , Shamsuddin HH , Yasinskaya YI , Bouri K , Lanthier ML , Sherman RE . Scientific and regulatory reasons for delay and denial of FDA approval of initial applications for new drugs, 2000–2012. JAMA. 2014;311(4):378‐384. 24449316 10.1001/jama.2013.282542

[379] Massard C , Michiels S , Ferte C , et al. High‐throughput genomics and clinical outcome in hard‐to‐treat advanced cancers: results of the MOSCATO 01 trial. Cancer Discov. 2017;7(6):586‐595. 28365644 10.1158/2159-8290.CD-16-1396

[380] Bertucci F , Goncalves A , Guille A , et al. Prospective high‐throughput genome profiling of advanced cancers: results of the PERMED‐01 clinical trial. Genome Med. 2021;13(1):87. 34006291 10.1186/s13073-021-00897-9 PMC8132379

[381] Dellett M , O'Hagan KA , Colyer HAA , Mills KI . Identification of gene networks associated with acute myeloid leukemia by comparative molecular methylation and expression profiling. Biomarkers Cancer. 2010;2:43‐55. 10.4137/BIC.S3185 PMC3783331 24179384

[382] Qu X , Davison J , Du L , et al. Identification of differentially methylated markers among cytogenetic risk groups of acute myeloid leukemia. Epigenetics. 2015;10(6):526‐535. 25996682 10.1080/15592294.2015.1048060 PMC4623036

[383] Valerio LG Jr , Choudhuri S . Chemoinformatics and chemical genomics: potential utility of in silico methods. J Appl Toxicol. 2012;32(11):880‐889. 22886396 10.1002/jat.2804

[384] Tratnyek PG , Bylaska EJ , Weber EJ . In silico environmental chemical science: properties and processes from statistical and computational modelling. Environ Sci Process Impacts. 2017;19(3):188‐202. 28262894 10.1039/c7em00053g

[385] Wolfram J , Ferrari M . Clinical cancer nanomedicine. Nano Today. 2019;25:85‐98. 31360214 10.1016/j.nantod.2019.02.005 PMC6662733

[386] Deirram N , Zhang C , Kermaniyan SS , Johnston APR , Such GK . pH‐responsive polymer nanoparticles for drug delivery. Macromol Rapid Commun. 2019;40(10):e1800917. 30835923 10.1002/marc.201800917

[387] Qiao Y , Wan J , Zhou L , et al. Stimuli‐responsive nanotherapeutics for precision drug delivery and cancer therapy. Wiley Interdiscip Rev Nanomed Nanobiotechnol. 2019;11(1):e1527. 29726115 10.1002/wnan.1527

[388] Xiao X , Wang K , Zong Q , Tu Y , Dong Y , Yuan Y . Polyprodrug with glutathione depletion and cascade drug activation for multi‐drug resistance reversal. Biomaterials. 2021;270:120649. 33588139 10.1016/j.biomaterials.2020.120649

[389] Chen Z , Wang Z , Gu Z . Bioinspired and biomimetic nanomedicines. ACC Chem Res. 2019;52(5):1255‐1264. 30977635 10.1021/acs.accounts.9b00079 PMC7293770

[390] Shen S , Xu X , Lin S , et al. A nanotherapeutic strategy to overcome chemotherapeutic resistance of cancer stem‐like cells. Nat Nanotechnol. 2021;16(1):104‐113. 33437035 10.1038/s41565-020-00793-0

[391] Guo Y , Zhang X , Sun W , et al. Metal–phenolic network‐based nanocomplexes that evoke ferroptosis by apoptosis: promoted nuclear drug influx and reversed drug resistance of cancer. Chem Mater. 2019;31(24):10071‐10084.

[392] Toki T , Iwasaki T , Ishii M . Topiramate blood levels during polytherapy for epilepsy in children. Am J Ther. 2019;26(1):e18‐e24. 27875371 10.1097/MJT.0000000000000529

[393] Lyons TG . Targeted therapies for triple‐negative breast cancer. Curr Treat Options Oncol. 2019;20(11):82. 31754897 10.1007/s11864-019-0682-x

[394] Owen CN , Shoushtari AN , Chauhan D , et al. Management of early melanoma recurrence despite adjuvant anti‐PD‐1 antibody therapy. Ann Oncol. 2020;31(8):1075‐1082. 32387454 10.1016/j.annonc.2020.04.471 PMC9211001

[395] Allen TM , Cullis PR . Liposomal drug delivery systems: from concept to clinical applications. Adv Drug Deliv Rev. 2013;65(1):36‐48. 23036225 10.1016/j.addr.2012.09.037

[396] Murakami T . Absorption sites of orally administered drugs in the small intestine. Expert Opin Drug Discov. 2017;12(12):1219‐1232. 28920464 10.1080/17460441.2017.1378176

[397] Meira Menezes T , Assis C , Lacerda Cintra AJ , et al. Binding mechanism between acetylcholinesterase and drugs pazopanib and lapatinib: biochemical and biophysical studies. ACS Chem Neurosci. 2021;12(24):4500‐4511. 34808043 10.1021/acschemneuro.1c00521

[398] de Jonge MJA , Hamberg P , Verweij J , et al. Phase I and pharmacokinetic study of pazopanib and lapatinib combination therapy in patients with advanced solid tumors. Invest New Drugs. 2013;31(3):751‐759. 23054212 10.1007/s10637-012-9885-8

[399] Monk BJ , Mas Lopez L , Zarba JJ , et al. Phase II, open‐label study of pazopanib or lapatinib monotherapy compared with pazopanib plus lapatinib combination therapy in patients with advanced and recurrent cervical cancer. J Clin Oncol. 2010;28(22):3562‐3569. 20606083 10.1200/JCO.2009.26.9571

[400] Olaussen KA , Commo F , Tailler M , et al. Synergistic proapoptotic effects of the two tyrosine kinase inhibitors pazopanib and lapatinib on multiple carcinoma cell lines. Oncogene. 2009;28(48):4249‐4260. 19749798 10.1038/onc.2009.277

[401] Tavallai S , Hamed HA , Grant S , Poklepovic A , Dent P . Pazopanib and HDAC inhibitors interact to kill sarcoma cells. Cancer Biol Ther. 2014;15(5):578‐585. 24556916 10.4161/cbt.28163 PMC4026080

[402] Dean AQ , Luo S , Twomey JD , Zhang B . Targeting cancer with antibody‐drug conjugates: promises and challenges. mAbs. 2021;13(1):1951427. 34291723 10.1080/19420862.2021.1951427 PMC8300931

[403] Tsujii S , Serada S , Fujimoto M , et al. Glypican‐1 is a novel target for stroma and tumor cell dual‐targeting antibody‐drug conjugates in pancreatic cancer. Mol Cancer Ther. 2021;20(12):2495‐2505. 34583978 10.1158/1535-7163.MCT-21-0335

[404] Salomon PL , Singh R . Sensitive ELISA method for the measurement of catabolites of antibody‐drug conjugates (ADCs) in target cancer cells. Mol Pharm. 2015;12(6):1752‐1761. 25738394 10.1021/acs.molpharmaceut.5b00028

[405] Glatt DM , Beckford Vera DR , Prabhu SS , et al. Synthesis and characterization of cetuximab‐docetaxel and panitumumab‐docetaxel antibody‐drug conjugates for EGFR‐overexpressing cancer therapy. Mol Pharm. 2018;15(11):5089‐5102. 30226780 10.1021/acs.molpharmaceut.8b00672

[406] Theunissen J‐W , Cai AG , Bhatti MM , et al. Treating tissue factor‐positive cancers with antibody‐drug conjugates that do not affect blood clotting. Mol Cancer Ther. 2018;17(11):2412‐2426. 30126944 10.1158/1535-7163.MCT-18-0471

[407] Chudasama V , Maruani A , Caddick S . Recent advances in the construction of antibody‐drug conjugates. Nat Chem. 2016;8(2):114‐119. 26791893 10.1038/nchem.2415

[408] O'Malley DM , Matulonis UA , Birrer MJ , et al. Phase Ib study of mirvetuximab soravtansine, a folate receptor alpha (FRalpha)‐targeting antibody‐drug conjugate (ADC), in combination with bevacizumab in patients with platinum‐resistant ovarian cancer. Gynecol Oncol. 2020;157(2):379‐385. 32081463 10.1016/j.ygyno.2020.01.037

[409] Rathore R , McCallum JE , Varghese E , Florea A‐M , Busselberg D . Overcoming chemotherapy drug resistance by targeting inhibitors of apoptosis proteins (IAPs). Apoptosis. 2017;22(7):898‐919. 28424988 10.1007/s10495-017-1375-1 PMC5486846

[410] Hunter AM , LaCasse EC , Korneluk RG . The inhibitors of apoptosis (IAPs) as cancer targets. Apoptosis. 2007;12(9):1543‐1568. 17573556 10.1007/s10495-007-0087-3

[411] Brunckhorst MK , Lerner D , Wang S , Yu Q . AT‐406, an orally active antagonist of multiple inhibitor of apoptosis proteins, inhibits progression of human ovarian cancer. Cancer Biol Ther. 2012;13(9):804‐811. 22669575 10.4161/cbt.20563 PMC3679100

[412] van Roosmalen IAM , Quax WJ , Kruyt FAE . Two death‐inducing human TRAIL receptors to target in cancer: similar or distinct regulation and function? Biochem Pharmacol. 2014;91(4):447‐456. 25150214 10.1016/j.bcp.2014.08.010

[413] Obeid MA , Aljabali AAA , Rezigue M , et al. Use of nanoparticles in delivery of nucleic acids for melanoma treatment. Methods Mol Biol. 2021;2265:591‐620. 33704742 10.1007/978-1-0716-1205-7_41

[414] Majidinia M , Mirza‐Aghazadeh‐Attari M , Rahimi M , et al. Overcoming multidrug resistance in cancer: recent progress in nanotechnology and new horizons. IUBMB Life. 2020;72(5):855‐871. 31913572 10.1002/iub.2215

[415] Ye DM , Ye SC , Yu SQ , et al. Drug‐resistance reversal in colorectal cancer cells by destruction of flotillins, the key lipid rafts proteins. Neoplasma. 2019;66(4):576‐583. 30943747 10.4149/neo_2018_180820N633

[416] Wu S , Wen F , Li Y , et al. PIK3CA and PIK3CB silencing by RNAi reverse MDR and inhibit tumorigenic properties in human colorectal carcinoma. Tumour Biol. 2016;37(7):8799‐8809. 26747178 10.1007/s13277-015-4691-5

[417] Koshkin PA , Chistiakov DA , Nikitin AG , et al. Analysis of expression of microRNAs and genes involved in the control of key signaling mechanisms that support or inhibit development of brain tumors of different grades. Clin Chim Acta. 2014;430:55‐62. 24412320 10.1016/j.cca.2014.01.001

[418] Piedra‐Carrasco N , Miguel L , Fabrega A , et al. Effectiveness of a double‐carbapenem regimen in a KPC‐producing Klebsiella pneumoniae infection in an immunocompromised patient. Microb Drug Resist. 2018;24(2):199‐202. 28708458 10.1089/mdr.2017.0129

[419] Neufeld L , Yeini E , Reisman N , et al. Microengineered perfusable 3D‐bioprinted glioblastoma model for in vivo mimicry of tumor microenvironment. Sci Adv. 2021;7(34):eabi9119. 34407932 10.1126/sciadv.abi9119 PMC8373143

[420] Aghamiri S , Mehrjardi KF , Shabani S , Keshavarz‐Fathi M , Kargar S , Rezaei N . Nanoparticle‐siRNA: a potential strategy for ovarian cancer therapy? Nanomedicine (Lond). 2019;14(15):2083‐2100. 31368405 10.2217/nnm-2018-0379

[421] Jia L , Li Z , Shen J , et al. Multifunctional mesoporous silica nanoparticles mediated co‐delivery of paclitaxel and tetrandrine for overcoming multidrug resistance. Int J Pharm. 2015;489(1‐2):318‐330. 25956050 10.1016/j.ijpharm.2015.05.010

[422] Markman JL , Rekechenetskiy A , Holler E , Ljubimova JY . Nanomedicine therapeutic approaches to overcome cancer drug resistance. Adv Drug Deliv Rev. 2013;65(13‐14):1866‐1879. 24120656 10.1016/j.addr.2013.09.019 PMC5812459

[423] Saraswathy M , Gong S . Different strategies to overcome multidrug resistance in cancer. Biotechnol Adv. 2013;31(8):1397‐1407. 23800690 10.1016/j.biotechadv.2013.06.004

[424] Hu C‐MJ , Zhang L . Nanoparticle‐based combination therapy toward overcoming drug resistance in cancer. Biochem Pharmacol. 2012;83(8):1104‐1111. 22285912 10.1016/j.bcp.2012.01.008

[425] Tang Y , Soroush F , Tong Z , Kiani MF , Wang B . Targeted multidrug delivery system to overcome chemoresistance in breast cancer. Int J Nanomedicine. 2017;12:671‐681. 28176940 10.2147/IJN.S124770 PMC5268372

[426] Liu Q , Li J , Pu G , Zhang F , Liu H , Zhang Y . Co‐delivery of baicalein and doxorubicin by hyaluronic acid decorated nanostructured lipid carriers for breast cancer therapy. Drug Deliv. 2016;23(4):1364‐1368. 25874959 10.3109/10717544.2015.1031295

[427] Zou W , Sarisozen C , Torchilin VP . The reversal of multidrug resistance in ovarian carcinoma cells by co‐application of tariquidar and paclitaxel in transferrin‐targeted polymeric micelles. J Drug Target. 2017;25(3):225‐234. 27616277 10.1080/1061186X.2016.1236113

[428] Chen Y , Zhang W , Huang Y , Gao F , Sha X , Fang X . Pluronic‐based functional polymeric mixed micelles for co‐delivery of doxorubicin and paclitaxel to multidrug resistant tumor. Int J Pharm. 2015;488(1‐2):44‐58. 25899286 10.1016/j.ijpharm.2015.04.048

[429] Akbar MU , Zia KM , Nazir A , Iqbal J , Ejaz SA , Akash MSH . Pluronic‐based mixed polymeric micelles enhance the therapeutic potential of curcumin. AAPS PharmSciTech. 2018;19(6):2719‐2739. 29978290 10.1208/s12249-018-1098-9

[430] Sivak L , Subr V , Tomala J , et al. Overcoming multidrug resistance via simultaneous delivery of cytostatic drug and P‐glycoprotein inhibitor to cancer cells by HPMA copolymer conjugate. Biomaterials. 2017;115:65‐80. 27886555 10.1016/j.biomaterials.2016.11.013

[431] Meng L , Xia X , Yang Y , et al. Co‐encapsulation of paclitaxel and baicalein in nanoemulsions to overcome multidrug resistance via oxidative stress augmentation and P‐glycoprotein inhibition. Int J Pharm. 2016;513(1‐2):8‐16. 27596118 10.1016/j.ijpharm.2016.09.001

[432] Xiao B , Ma L , Merlin D . Nanoparticle‐mediated co‐delivery of chemotherapeutic agent and siRNA for combination cancer therapy. Expert Opin Drug Deliv. 2017;14(1):65‐73. 27337289 10.1080/17425247.2016.1205583 PMC5531052

[433] Zhang C‐G , Zhu W‐J , Liu Y , et al. Novel polymer micelle mediated co‐delivery of doxorubicin and P‐glycoprotein siRNA for reversal of multidrug resistance and synergistic tumor therapy. Sci Rep. 2016;6:23859. 27030638 10.1038/srep23859 PMC4814909

[434] Reddy TL , Garikapati KR , Reddy SG , et al. Simultaneous delivery of paclitaxel and Bcl‐2 siRNA via pH‐sensitive liposomal nanocarrier for the synergistic treatment of melanoma. Sci Rep. 2016;6:35223. 27786239 10.1038/srep35223 PMC5081533

[435] Wang H , Li F , Du C , Wang H , Mahato RI , Huang Y . Doxorubicin and lapatinib combination nanomedicine for treating resistant breast cancer. Mol Pharm. 2014;11(8):2600‐2611. 24405470 10.1021/mp400687w

[436] Liu X , Wu Z , Guo C , et al. Hypoxia responsive nano‐drug delivery system based on angelica polysaccharide for liver cancer therapy. Drug Deliv. 2022;29(1):138‐148. 34967268 10.1080/10717544.2021.2021324 PMC8725898

[437] Deb B , George IA , Sharma J , Kumar P . Phosphoproteomics profiling to identify altered signaling pathways and kinase‐targeted cancer therapies. Methods Mol Biol. 2020;2051:241‐264. 31552632 10.1007/978-1-4939-9744-2_10

[438] Lim YP . Mining the tumor phosphoproteome for cancer markers. Clin Cancer Res. 2005;11(9):3163‐3169. 15867208 10.1158/1078-0432.CCR-04-2243

[439] Miryala SK , Anbarasu A , Ramaiah S . Systems biology studies in *Pseudomonas aeruginosa* PA01 to understand their role in biofilm formation and multidrug efflux pumps. Microb Pathog. 2019;136:103668. 31419460 10.1016/j.micpath.2019.103668

